# A realist review of medication optimisation of community dwelling service users with serious mental illness

**DOI:** 10.1136/bmjqs-2023-016615

**Published:** 2023-12-07

**Authors:** Jo Howe, Maura MacPhee, Claire Duddy, Hafsah Habib, Geoff Wong, Simon Jacklin, Sheri Oduola, Rachel Upthegrove, Max Carlish, Katherine Allen, Emma Patterson, Ian Maidment

**Affiliations:** 1 Pharmacy School, College of Health and Life Sciences, Aston University, Birmingham, UK; 2 School of Nursing, The University of British Columbia, Vancouver, British Columbia, Canada; 3 Primary Care Health Sciences, University of Oxford, Oxford, UK; 4 Pharmacy School, Aston University College of Health and Life Sciences, Birmingham, UK; 5 School of Pharmacy and Bioengineering, Keele University, Keele, UK; 6 School of Health Sciences, University of East Anglia, Norwich, UK; 7 Institute for Mental Health, University of Birmingham, Birmingham, UK; 8 Birmingham Early Intervention Service, Birmingham Women's and Children's NHS Foundation Trust, Birmingham, UK; 9 Birmingham and Solihull Mental Health NHS Foundation Trust, Birmingham, UK

**Keywords:** shared decision making, compliance, medication safety, mental health, patient-centred care

## Abstract

**Background:**

Severe mental illness (SMI) incorporates schizophrenia, bipolar disorder, non-organic psychosis, personality disorder or any other severe and enduring mental health illness. Medication, particularly antipsychotics and mood stabilisers are the main treatment options. Medication optimisation is a hallmark of medication safety, characterised by the use of collaborative, person-centred approaches. There is very little published research describing medication optimisation with people living with SMI.

**Objective:**

Published literature and two stakeholder groups were employed to answer: What works for whom and in what circumstances to optimise medication use with people living with SMI in the community?

**Methods:**

A five-stage realist review was co-conducted with a lived experience group of individuals living with SMI and a practitioner group caring for individuals with SMI. An initial programme theory was developed. A formal literature search was conducted across eight bibliographic databases, and literature were screened for relevance to programme theory refinement. In total 60 papers contributed to the review. 42 papers were from the original database search with 18 papers identified from additional database searches and citation searches conducted based on stakeholder recommendations.

**Results:**

Our programme theory represents a continuum from a service user’s initial diagnosis of SMI to therapeutic alliance development with practitioners, followed by mutual exchange of information, shared decision-making and medication optimisation. Accompanying the programme theory are 11 context-mechanism-outcome configurations that propose evidence-informed contextual factors and mechanisms that either facilitate or impede medication optimisation. Two mid-range theories highlighted in this review are supported decision-making and trust formation.

**Conclusions:**

Supported decision-making and trust are foundational to overcoming stigma and establishing ‘safety’ and comfort between service users and practitioners. Avenues for future research include the influence of stigma and equity across cultural and ethnic groups with individuals with SMI; and use of trained supports, such as peer support workers.

**PROSPERO registration number:**

CRD42021280980.

**WHAT IS ALREADY KNOWN ON THIS TOPIC**

- Medication optimisation is challenging for both people living with severe mental illness and their prescribing clinicians; medication non-adherence is common.

**WHAT THIS STUDY ADDS**

- Effective optimisation of medication requires a person-centred approach embedded throughout a service user’s journey from initial diagnosis to effective medication co-management with practitioners.

**HOW THIS STUDY MIGHT AFFECT RESEARCH, PRACTICE OR POLICY**

- Research is needed in multiple aspects of medication optimisation, including transition from acute care to community, the role of trained peer support workers and practitioner awareness of unique needs for individuals from ethnic and cultural minority groups.

## Introduction

### Medication optimisation

 Severe mental illness (SMI) is a significant global healthcare burden with rates increasing throughout the world.^1^ The term SMI includes diagnoses of schizophrenia, non-organic psychosis, bipolar disorder, personality disorder and any other severe and enduring mental illness.^2^ Medications are a key treatment for SMI, but medication side effects can contribute to chronic physical illness (eg, diabetes, cardiovascular disease), a diminished quality of life and a decreased lifespan.^3 4^ Complex medication regimens are often used to treat SMI; dosing can be a delicate balance between overprescribing and underprescribing, based on individual service user’s (SUs) unique needs.^5^

Given the complex nature of SMI medication management and the need to consider issues such as risk of relapse, serious side-effect profiles and potential drug-drug interactions, medication safety is of paramount importance to SUs and practitioners.^6^,^8^ Psychotropics have many safety challenges. Metabolic adverse events including weight gain, changes in cholesterol and triglyceride levels and increases in blood sugar are a particular concern.^9^ Many psychotropics have anticholinergic effects; these include constipation, falls and confusion.^10 11^ This confusion could potentially worsen the negative and cognitive symptoms of schizophrenia and increase the risk of medication errors.^7^

Since 2008, the global Institute for Healthcare Improvement (IHI) and its country affiliates, such as the UK’s Health Foundation, have advocated for inclusion of SUs and person-centred care approaches when identifying best practices, strategies pertaining to patient safety and quality of care delivery^12^ as well as promoting shared decision-making (SDM) in healthcare systems.^13^ The original Triple Aim IHI framework consisted of three pillars for advancing quality and safety: enhanced population health, positive SU experiences and cost-effectiveness.^12^ The original framework has expanded to Quadruple Aim, including staff experience.^12^ These IHI frameworks highlight how the SU voice is an integral component of all healthcare quality and safety initiatives. Medication optimisation, a hallmark of medication safety, is defined as ‘a person-centred approach to safe and effective medicines use, to ensure people obtain the best possible outcomes from their medicines’.^14^ Effective medication optimisation involves the perspectives of SUs with lived experience of taking medications.^15^ Multidisciplinary care delivery for SMI is more effective when SUs play a central role in medication decision-making.^16^

Failure to optimise medication is often attributed to SU non-adherence, practitioner underprescribing or overprescribing or overtreatment, including polypharmacy.^216^,^18^ Management of SMI is particularly challenging with reported non-adherence rates as high as 50%.^19 20^ Non-adherence^21^ and overprescribing occur more frequently in ethnic minority communities, as do physical illnesses, such as diabetes and cardiovascular disease.^14 22^ In general, the lowest possible medication dose is recommended to control SMI symptoms,^23^ however higher doses are often prescribed by practitioners concerned about relapse.^24^ Poorly managed SMI increases relapse rates, hospitalisation and is associated with unemployment, homelessness, disrupted education, substance misuse, physical health problems, self-harm and excess mortality.^2 17 18 25^ A systematic review and meta-analysis of studies from Asia, Europe and North America found that non-adherence within the SMI population is the strongest predictor of relapse.^26^

### Shared decision-making

Person-centred approaches, such as SDM between key practitioner groups (eg, pharmacy, medicine, nursing), SUs with SMI and family carers, are associated with increased SU medication adherence and improved practitioner prescribing practices.^7 17 19 20^ There is, however, limited research on what needs to happen, how and when in the SU-practitioner relationship to promote person-centred SDM and ultimately, medication optimisation for SUs with SMI.^4 17 27^ Assumptions are often made about intervention effectiveness only from practitioner’s viewpoints.^28^ The implementation of SDM can be hindered by practitioners’ beliefs about SDM. A Netherlands-based study compared practitioner reports of SDM use with direct observations of their SU interactions.^29^ Practitioners reported using SDM as their usual decision-making style, but in observations, there was low engagement with SUs. The authors described practitioners as ‘unconsciously incompetent in SDM’. Therefore, developing knowledge on how to implement effective person-centred approaches that promote medication optimisation is needed.

### Present research

We conducted a realist review on medication optimisation with community dwelling SUs living with SMI. We focused on community dwelling as most SMI SUs live in the community, where there are greater opportunities for them to exercise control over their medication regime (eg, by omitting doses or via non-adherence).

We synthesised data from academic literature and drew on perspectives of community-based stakeholders with lived experience of SMI, informal (family) carers and mental health practitioners caring for SUs with SMI. A realist review can uncover important contextual factors affecting outcomes.^30^ We constructed a programme theory comprising a series of testable hypotheses, known as context-mechanism-outcome configurations (CMOCs), to explain a potential SU-practitioner journey from initial diagnosis to trusting therapeutic alliance, SDM and medication optimisation.

Realist reviews have become increasingly popular within the quality and safety literature to explain how and why interventions work. Realist reviews have been used to investigate junior doctors’ antimicrobial prescribing^31^; safety-netting practices in primary care^32^ and medication management for community dwelling seniors on complex medication regimens.^33^ Realist reviews address research questions about what works, for whom, under what circumstances and how, and are a valuable methodological alternative or complement to other forms of evidence synthesis, such as systematic reviews.^30 34^

### Research objectives

The overall objective for this realist review was to use published literature, alongside lived experience and practitioner stakeholder groups to understand: What works for whom and in what circumstances to optimise medication for community dwelling SUs with SMI.

## Methods

We conducted a five-stage realist review. Our review protocol was published^35^ and registered a priori with PROSPERO (CRD42021280980). We used academic literature as well as feedback and advice from our stakeholder groups (lived experience group (LEG) and practitioner group (PG) to refine our programme theory, and create a series of testable CMOCs.^36^ The LEG comprised six lived experience stakeholders from Birmingham and Solihull Mental Health NHS Foundation Trust (BSMHFT) Lived Experience Advisory Research (LEAR) Group and two additional individuals with lived experience from outside LEAR (who were recruited to facilitate discussions). The PG comprised healthcare practitioners from the UK caring for SUs with SMI. The practitioners were recruited from personal networks and via social media advertisements.

Our CMOCs describe specific contexts associated with important outcomes related to medication optimisation, such as therapeutic alliance formation, and they articulate the mechanisms that trigger these outcomes. In this project, we have defined the following:

*Setting*: adults with SMI taking medication living in the community.

*Intervention*: any intervention to optimise medication usage for SUs living with SMI.

*Context*: situational factors that create conditions necessary to trigger underlying mechanisms.

*Mechanisms*: hidden, psychological processes that link specific contexts to intended outcomes.

*Outcomes*: quality of life, adherence, adverse events, disease symptoms, economic.

### Stage 1: objectives, initial programme theory

#### Objectives

To conduct a realist review using published literature alongside lived experience and practitioner stakeholder input to understand: What works for whom and in what circumstances to optimise medication use for community dwelling SUs with SMI.

#### Development of initial programme theory

We developed an initial programme theory (IPT), a testable explanation of how and why medication optimisation is supposed to work for people living with SMI. Prior to the application for funding, IM initially consulted with stakeholders with lived experience from BSMHFT. These conversations helped shape the initial funding application and formed the tentative foundations for the IPT. Through retelling accounts of their own experiences of SDM within mental health services, stakeholders stressed the importance of the therapeutic relationship in generating positive outcomes. Once the project started, JH liaised with subject matter experts known to the project team to gain a wider understanding of SMI, medication utilisation and SU-practitioner SDM. Additionally, an informal literature search primarily using Google Scholar was conducted. This step was used to gain an understanding of the factors influencing SDM within SMI. Additional publications recommended by the project team were also considered. The IPT was developed from these sources.

### Stage 2: literature search

A formal literature search was conducted in January 2022 by CD across eight bibliographic databases (MEDLINE, Embase, PsycINFO, CINAHL, Cochrane Library, Scopus, Web of Science Core Citation Indexes (Science Citation Index Expanded, Social Sciences Citation Index, Arts & Humanities Citation Index, Emerging Sources Citation Index, Conference Proceedings Citation Index, Book Citation Index) and Sociological Abstracts). Our searches combined free text and subject heading terms for SMI, with terms describing medication or medication optimisation, and a comprehensive list of terms reflecting our project focus on SDM and SU-practitioner relationships. The LEG and PG helped to identify key concepts used in our search strategy. Our original protocol indicated that we would run searches in Google Scholar, but this was deemed unnecessary following screening, in light of the volume of literature already retrieved. In response to PG and LEG feedback, additional targeted searches were conducted in June 2022 to identify material relating to internet use, peer support and tapering medication.

Full details of our search strategies are available in online supplemental file 1.

### Stage 3: screening and inclusion

Inclusion criteria focused on community dwelling adults (aged 18+ years) living with SMI and taking antipsychotic medication. Studies limited to inpatient settings or focused on diagnoses outside of SMI were excluded. Full details of the inclusion/exclusion criteria can be found in table 1.

**Table 1:** Review: inclusion and exclusion criteria

Inclusion criteria	Exclusion criteria
Community dwelling adults living with SMI, using medicationTheir family or carersPractitioners involved in their care	Inpatient settings only
Interventions to optimise medication usageOrExperience of medication management and use	SDM tools
All study designs	No focus on the SDM process
All countries	Not SMI
English language	Eating disorders

SDMshared decision-makingSMIsevere mental illness

### Screening

The results of the main search were screened by title and abstract by JH using RAYYAN (a web-based tool designed to assist with screening of title and abstract). In line with realist methodology, titles and abstracts were screened to determine relevance to the developing programme theory. A random 10% sample was screened in duplicate by MMP. Uncertainties were resolved via discussions with JH and MMP. Full-text screening was initially completed in EndNote X9 by JH on documents published from 2014 onwards. The decision to focus on this timepoint was based on a significant increase in documents with SDM content during this time period. For full-text articles to be included at this stage, they had to contain data relevant to the developing programme theory with details on the SDM process in relation to prescribing, switching, tapering and taking antipsychotic medication. All full-text documents were assigned a star rating of one to five in EndNote by JH, based on a global judgement of each document’s likely relevance, richness and rigour to confirm or dispute the developing programme theory. One-star documents were deemed irrelevant and rejected. Two-star documents were deemed ‘unsure’, and these were subsequently discussed with MMP and reallocated. Three-star documents were deemed irrelevant for programme theory development but potentially useful for background material. Four-star documents were deemed relevant to CMOC development and programme theory refinement. Five-star documents were deemed the most conceptually rich and relevant to CMOC development and programme theory refinement.

Pre-2014 documents and documents obtained via citation searching and personal networks were purposively screened and analysed by CD, JH, MMP and HH but were not categorised with a star rating as they were chosen due to perceived high relevance, richness and rigour.

### Stage 4: data extraction, analysis

#### Data extraction

Document characteristics were extracted to an Excel spreadsheet by HH (online supplemental file 2).

#### Data analysis

A coding framework was iteratively and inductively developed and tested by MMP, JH and HH to organise relevant data. The coding framework was developed in conjunction with the LEG and PG. The framework was tested using a subset of papers from the review. MMP, HH and JH tested each iteration of the framework, and codes were added to ensure the framework encapsulated all relevant data. The framework comprised three conceptual buckets that reflected a more abstracted level of data, for example, decision-making, medication management interventions and therapeutic alliance. In total, there were nine revisions to the framework. The final framework in online supplemental file 3 demonstrates the different levels of abstraction.

Coding of post-2014 five-star full-text documents was completed in NVivo by MMP and HH with a 10% check in duplicate by JH. Extracts of data were coded to nodes (termed parent nodes in NVivo) reflecting conceptual buckets. Extracts of data were coded against subnodes (called child nodes in NVivo) and multiple nodes if appropriate.

Once all five-star papers were coded and discussed with the PG and LEG, a pragmatic decision was made to focus the review on decision-making and therapeutic alliance. Coded data based on the coding framework were initially extracted and imported in Microsoft Word by MMP and HH. Tentative CMOCs were developed and refined through ongoing discussions with JH, MMP and HH. Approximately 30 hours of collaborative discussion took place over MS Teams.

### Stage 5: data synthesis, CMOC development and programme theory refinement

Each tentative CMOC was individually reviewed, verified against the underpinning evidence and refined. Similar CMOCs were amalgamated where appropriate. These CMOCs were iteratively refined by checking remaining data from NVivo nodes and extracting relevant examples. Further refinement of the programme theory and CMOCs occurred using data from relevant four-star papers, pre-2014 papers, papers from additional searches and discussions with the project team, LEG and PG. The finalised set of CMOCs and a refined programme theory were discussed and validated with the PG, the LEG and the wider project team. The refined CMOCs, supporting evidence and document origin (eg, post-2014, via citation search or personal networks) can be found in online supplemental file 4.

## Results

**Figure 1:**
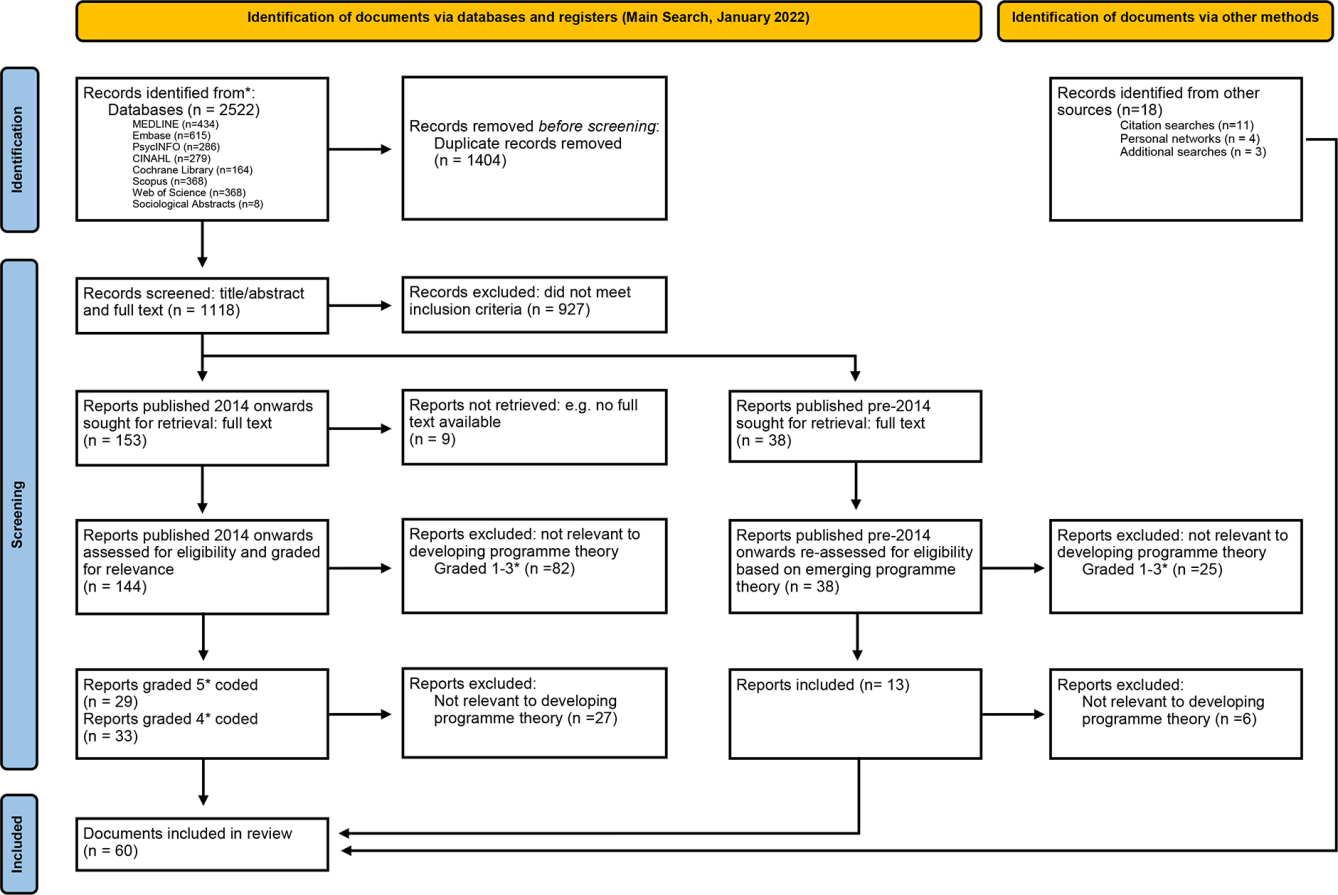
Preferred Reporting Items for Systematic Reviews and Meta-Analyses 2020 flow diagram for new systematic reviews which included searches of databases, registers and other sources. Adapted from Page *et al*.^114^

Our main search identified 1118 unique results. After title and abstract screening, 144 documents published from 2014 onwards were screened in full text. Twenty-nine papers were assigned a five-star rating and coded in NVivo. Thirty-three papers were assigned a four-star rating. Following the decision to narrow the focus, of these 62 papers, 27 were rejected leaving a total of 35 four-star and five-star papers in the review. Eighteen papers were identified via additional searches, citation searches and personal contacts. Seven of the pre-2014 papers were deemed relevant taking the total number of papers contributing to the review to 60 (35+18+7). The inclusion criteria associated with SMI varied between the included papers. Twenty-eight papers included schizophrenia or schizoaffective disorder,^37^,^64^ 3 papers included borderline personality disorder,^38 58 59^ 9 papers included bipolar disorder,^4349 51 54 59^,^61 65 66^ 19 papers included psychosis/psychotic illness,^3840 43^,^45 51 59 61 67^ 20 papers had a broad definition, for example, SMI or people taking antipsychotic/psychotropic medication,^3738 66 75 78^,^93^ 3 papers included less severe forms of mental illness alongside SMI in their population samples,^51 60 66^ 2 papers did not have a patient population and focused on consultant psychiatrists^94^ and secondary care mental health pharmacists.^95^ Several papers included more than one type of SMI. Our searching and screening processes are summarised in figure 1.

Table 2 includes our refined programme theory and 11 CMOCs underpinning the theory. Our refined programme theory describes a journey of medication optimisation for SUs with SMI that begins with initial diagnosis and culminates in a therapeutic alliance characterised by underlying trust, mutual information exchange and SDM. There are potential barriers and facilitators along the way, represented by positive and negative CMOCs. The journey includes practitioners, SUs with their family and social network and other information sources (eg, internet, peer support workers).

**Table 2:** Refined programme theory and underpinning context-mechanism-outcome (CMOC) configurations

*Refined programme theory‘When service users (SU) are first diagnosed with serious mental illness (SMI), a diagnosis which is frightening to them, they seek out information about their illness*.*SUs omedications want practitioner support and practitioner advice they can understand and apply to their current and ongoing needs*.*SUs may seek out individuals with lived experience to validate the experiences they are having, and to learn how others effectively manage living with SMI. As SUs gather information from diverse sources (practitioners, social supports, internet), they are constantly weighing up the pros and cons of medication decisions*.*It is important to SUs to forge positive working relationships with practitioners who will listen to them, respectfully consider their needs and support their medication decisions whenever possible. SUs are regularly facing lifestyle challenges, some with high stakes, such as pregnancy or serious health side effects. If and when SUs have established therapeutic relationships with practitioners who have their best interests at heart and are competent in their field of expertise, SUs are more apt to seek them out for shared information exchange and decision-making*.*Regardless of the strength of the SU-practitioner relationship, in high stakes situations, trust is fragile; trust is based on ongoing evidence of practitioners’ motivations to support them. Similarly, SUs need ongoing and non-judgemental support from family members and their social network, including peer support workers’.*	
CMOC	Evidence sources
CMOC 1: *first contact*When an SU with SMI is first diagnosed, is medicated and has coercive, dehumanising* experiences with practitioners (C), this often derails the development of trusting therapeutic alliances (O) because of feelings of powerlessness (M) and stigmatisation (M).	38 44 45 68 74 77 79 81 88 95
CMOC 2: *relief*When an SU with SMI is first diagnosed and is medicated, validation and normalisation of their condition by a respectful, supportive practitioner (C) results in increased relief, hope and optimism (O) due to decreased stigmatisation of living with SMI (M) and increased reassurance (M) that they have a treatable condition.	43 62 68 71 74 88
CMOC 3: *dismissal*When an SU with SMI on medications realises practitioners are withholding medication information, and/or excluding, ignoring or dismissing them from medication decisions (C), they are apt to withdraw from the practitioner relationship and make their own medication decisions (O), due to mistrust (M) in the practitioners’ interest in them and their need for more control (M) over decisions affecting their lives.	4043 45 59 62 67 68 71 76 77 79 81 83
CMOC 4: *being heard*From the start of their relationship onwards, when an SU with SMI on medications is actively engaged by a respectful, supportive practitioner who takes an interest in them and their issues and concerns about their illness, medication and side effects (C), they are more apt to forge a therapeutic alliance with their practitioner (O), because they feel heard and listened to (M) and they trust (M) in the practitioner’s motivations to help them better manage their medications and illness.	3943 46 47 53 57 60 62 68 70 78 81
CMOC 5: *practitioner information exchange*From the start of the therapeutic relationship onwards, when an SU with SMI feels comfortable accessing their practitioner for honest, easy-to-understand and personalised information about their medications (C), they are apt to use the information to prepare for and to cope better with medications and side effects (O), due to development of mutual trust (M) and respect (M) in each other and in the information being exchanged.	43 4550 52 56 62 67 70 75
CMOC 6: *seeking more information*Whenever an SU with SMI on medications desires additional information about their illness, medications and potential side effects (C), they will often seek out accessible, easy-to-understand information from a variety of non-practitioner sources (eg, peers, internet) they perceive to be trustworthy and credible (O), due to need for increased knowledge (M), increased reassurance (M) and greater control (M) with respect to medication and life decisions.	37 56 67 68 71 77 78 80 85 86
CMOC 7: *confiding and negotiating in a safe way*When an SU with SMI on medications has continuity over time in a respectful, trusting therapeutic alliance with practitioners who openly discuss and make collaborative medication decisions with them, even when there are disagreements (C), they are more apt to confide in and to negotiate with their practitioners about their medication issues and management plans (O), due to a sense of safety with their practitioners (M), and increased belief (M) in themselves to manage their lives.	38 4042
CMOC 8: *perceived risks*When SUs with SMI desire to taper, change or discontinue their medication regimen (C), their clinicians may resist sharing information with them and may not support them (O) because they judge that doing so may put themselves, the patient and others at risk (M) if adverse outcomes occur (eg, harm to self or others).	40 42 45 55 76 83 86 88 90 93 94
CMOC 9: *family and social supports*When an SU with SMI has support from family and social network members who believe in them and want the best for them (C), they are apt to feel more confident in following through with prescribed medication plans (O) due to increased belief (M) in their capacity to handle ongoing challenges and a sense of safety (M) among people looking after their well-being.	54 57 61 64 66 72 73 84 86
CMOC 10: *fear and guilt*When an SU with SMI is aware that their family members are fearful about the consequences from medication changes and want them to maintain medications as prescribed (C), they may continue on the medications against their will or secretly discontinue/change their medications (O) to avoid conflict (M) and/or withdrawal of their family’s support (M) for them.	51 54 57 61 63 68 73 80 84 85 88
CMOC 11: *peer support workers*When SUs with SMI have access to peer support workers with shared lived experiences who talk with them about SMI and life skills management, including medications and side effects (C), they are apt to experience a positive impact on their mental, physical and social-emotional health (O) because they feel validated (M) less stigmatised (M) and reassured (M) that they can have a productive, fulfilling lives with SMI.	39 41 65 69 81 86

* Our lived experience group asked us to include ‘dehumanising’, based on their initial experiences with SMI diagnosis and management.

In table 2, CMOCs 1 (first contact) and 2 (relief) are associated with initial diagnosis. The literature highlights the importance of positive first encounters with healthcare services. Negative, coercive experiences can derail practitioner-SU trust formation, while positive experiences can decrease internalised stigma and reassure SUs that their condition is treatable.

CMOC 3 (dismissal) depicts how dismissal and devaluing of SUs by practitioners impedes the establishment of trust, which is a foundational component to therapeutic relationships.

CMOC 4 (being heard) illustrates how development of the therapeutic alliance is dependent on respectful, supportive practitioners willing to listen to and seriously consider SUs’ needs and concerns.

CMOCs 5 (practitioner information exchange) and 6 (seeking more information) represent SUs’ desire for credible, trustworthy information about diagnosis and medication that is personalised to them and the role of medication in treating their illness, including possible side effects. Regardless of information obtained from practitioners, SUs typically seek out additional information to obtain new knowledge, a greater sense of reassurance and more control over medication and life decisions.

As described in CMOC 7 (confiding and negotiating in a safe way), a hallmark of strong and effective therapeutic alliances is the ability of practitioners to support SUs, even if they disagree with their medication decisions. SUs feel safe in this type of alliance and are more apt to collaboratively plan their care with a trusted practitioner.

In contrast, CMOC 8 (perceived risks) illustrates why some practitioners might find it challenging to meet SUs’ wishes to taper their medication due to risk about potential adverse outcomes, such as relapse and its consequences.

CMOCs 9 (family and social supports), 10 (fear and guilt) and 11 (peer supports) are related to non-practitioner sources of support for individuals with SMI. The family can be a safety net and positive support for SUs (CMOC 9), or the family can be fearful of making medication changes (CMOC 10), resulting in negative SU emotions, such as fear and guilt. In these situations, SUs may feel pressured to conform to family wishes to avoid conflict and ensure that family support is not withdrawn. Peer support workers (CMOC 11) are a promising source of support to SUs, because they validate SUs’ feelings, given their lived experience with SMI. However, research on peer support worker roles in medication optimisation was lacking.

## Discussion

The patient safety literature has demonstrated that higher levels of safety are achievable by ensuring the voice of SUs and other stakeholders are part of quality improvement efforts.^96^ Medication optimisation is an important component of patient safety, especially for SUs with SMI, where medication is a key treatment strategy.^16^ Our programme theory and CMOCs highlight how person-centred care approaches such as providing relevant, useful information and support through practitioners and others can lead to safe medication use (ie, medication optimisation). The CMOCs outlined above provide testable, causal explanations for outcomes, detailing by whom, when and how these happen.

### Comparisons with existing literature and theory

A valuable aspect of the realist approach is the potential to use formal or substantive theories to further explain and buttress inferences about underlying mechanisms or drivers for individuals’ actions.^30^ Based on our reading of the included documents and recommendations from our stakeholder groups, we discuss below two theories of particular importance to the development of a therapeutic alliance, which is a necessary condition for effective optimisation of medication.

### Supported decision-making theory

Supported decision-making theory emphasises practitioners’ roles in assisting SUs with decision-making based on their needs and preferences. Supported decision-making theory has legal and ethical associations with the 2006 United Nations Convention of Individuals’ Rights and Disabilities, which stipulated that no person should be appointed as a decision-maker for an individual who has the capacity to make their own decisions with appropriate supports.^97^ Supported decision-making encompasses person-centred planning, advocacy, communications, interpretive supports and representational supports (eg, peer support workers, family and social networks). The central question practitioners should ask themselves is: ‘What supports are needed to ensure this person can best exercise their rights?’^97^

Although research into supported decision-making and SMI is rare, qualitative researchers found that the timing and types of practitioner supports made a difference to individuals with SMI, particularly with respect to confidence and self-control.^98 99^ Researchers from one study created different thematic labels for SUs: SUs with capacity to make their own cogent decisions were ‘inward experts’, while SUs during periods of acute unwellness were ‘outward entrustors’, entrusting practitioners to guide their care management. SUs’ variable needs for supported decision-making required different practitioner roles, such as practitioners as facilitators (eg, sharing information openly and honestly) and as collaborators (eg, promoting SDM).^98^

In a recent systematic review of supported decision-making for SUs with SMI in clinical practice settings,^100^ a limited number of papers included in the review examined stakeholders’ perspectives of supported decision-making. Stakeholders, including SUs, family members and practitioners, all agreed to the importance of supported decision-making. Practitioner misconceptions about differences between SUs’ rights and preferences, however, were barriers to implementation success. If supported decision-making is a necessary condition for SU-practitioner SDM and medication optimisation, practitioners will need to understand SUs’ legal rights, and to engage in roles (eg, facilitator, collaborator) that promote SUs’ decision-making autonomy.^98^

### Trust formation theory

Our findings make clear that, for SUs with SMI, ongoing alliance-building and confidence in their capacity to share in decisions and manage their lives depends on trust: trust in practitioners and trust/belief in themselves. Trust formation theory defines trust as ‘a psychological state comprising the intention to accept vulnerability based on positive expectations of the intentions or behaviour of another’.^101^ Our programme theory proposes that trust between SUs and practitioners evolves with the development of the therapeutic alliance. An exploratory study of the role of trust in medication management within mental health services^102^ supported our realist review findings that practitioners’ reluctance to share useful information in an open and honest way can create mistrust and worsen medication adherence. Ultimately, mistrust obstructs collaborative medication management.^102 103^

The wider literature provides some evidence of how mutual trust formation enables engagement, disclosure and collaboration in mental healthcare. Corroborating our findings, a qualitative study from the UK found that practitioners’ open communications and therapeutic listening promoted and sustained the development of mutual trust over time.^104^ More recent literature suggests that the development of a trusting therapeutic alliance is enhanced by practitioners’ awareness and respect for SU preferences, such as types of treatment options (eg, medications or psychotherapy), and influenced by the characteristics of practitioners they work with (eg, professional background, gender, age and ethnicity).^105^ Even when all preferences cannot be accommodated, eliciting, discussing and acknowledging SU preferences is associated with stronger alliances.^105^

### The relationship between SDM, trust and information

CMOCs (4, 5, 7) associate SDM with active engagement of SUs and practitioners in open, transparent discussions and collaborative treatment planning based on mutual trust. CMOC 8, however, addresses the negative emotions and risks associated with the SU-practitioner relationship. The literature in our review focused predominantly on practitioner risks, such as concern for SU medication non-adherence and adverse SU outcomes. A recent review of qualitative studies^106^ discussed risk-taking from the perspectives of SUs and practitioners. With shared risk-taking, both parties jointly reflect and address inherent risks to any decision, particularly from a safety perspective. Some evidence suggests that mutually identifying and preventing or mitigating risks can actually strengthen the alliance and deepen trust.^107^ In the UK, the National Health Service recommends that risk assessment and management should be explained to SUs with SMI as soon as possible as part of SDM.^108^

CMOCs (6, 9, 11) pertain to non-practitioner sources of information and support, including the internet and social media, family and friends and peer supports with similar lived experience, although we identified very limited academic literature on how peer support workers can be used to optimise medication management.^39 41 65 69 81 86^ Questions exist with respect to peer support workers’ capacity to give accurate medication advice.^109^

### Future research directions

Future research needs to address non-practitioner sources of information and supports, (eg, peer support workers, families and friends, internet and social media). Our LEG and PG stakeholders both endorsed the importance of peer support workers and their potential roles in medication optimisation.

An area of burgeoning interest is online decision support tools to improve information sharing and communication between SUs and practitioners. A recent systematic review found mixed evidence for the effectiveness of decision support tools with SUs with SMI.^110^ This review included tools to assist with prioritising treatment preferences, crisis planning and advanced directives. More conclusive research is needed to evaluate the efficacy of online support tools, especially how they help or hinder therapeutic alliance development, SDM and medication optimisation for SUs with SMI.

Our review has highlighted an important evidence gap relating to equity, diversity and inclusivity (EDI) for SUs with SMI from racial and ethnic minority groups, seniors and other vulnerable populations. These groups were rarely mentioned in our review’s included papers. When SUs with SMI are members of minority groups and/or vulnerable populations, stigma can be compounded.^27 111^ Ultimately, the success of SU-practitioner relationships depends on reducing mistrust among SUs who have been stigmatised by SMI and by race/ethnicity, while enhancing practitioners’ awareness and commitment to EDI. In England, the Race Equality Foundation is a national charity that tracks and reports racial inequality in public services (https://raceequalityfoundation.org.uk/). Researchers working with this charity identified persistent healthcare inequalities for English minority groups. ‘Traumatic, inappropriate and discriminatory experiences of services can have a detrimental impact on chances for recovery, particularly if the same risk factors of bereavement, family breakdown, incarceration, poverty and exposure to racism continue to be present’.^27^

Community-based care models may be a more cost-effective way of caring for complex and vulnerable patients. Only a limited number of new care models (eg, team-based care, integrated care) have been well-described or evaluated for SUs with SMI.^112^ In one mapping review of integrated physical-mental care models for SUs with SMI, a number of concerns were identified, including practitioners’ negative biases and stigma towards SMI.^113^ As our review illustrates, the initial context, particularly the presence of any negative biases towards SMI, can derail the development of a therapeutic alliance between SUs and practitioners.

Another important consideration for future research centres around the skill and knowledge of individual practitioners to be able to fully engage in SDM. Our practitioner stakeholders informed us that they are often not taught how to have difficult conversations with SUs, leaving them underconfident with some of the more challenging aspects associated with engaging in SDM with this population. However, none of the papers included within this review discussed these issues.

### Strengths and limitations

We conducted our realist review within a 1-year timeline. To be as efficient as possible, we focused on the largest body of relevant literature published from 2014 onwards. We returned to pre-2014 literature after developing our CMOCs from the more recent literature to identify earlier data relating to the CMOCs.

Our research team were English speakers only, therefore we excluded any non-English documents from our searches. We adopted an inclusive approach to the population and used a broad definition of SMI. However, there may be differences in the approach to medication optimisation in the different diagnoses (eg, bipolar, psychosis, personality disorder). Future research should consider a more granular approach and compare and contrast across different types of SMI. We focused on SDM between SUs and practitioners. As such, documents pertaining solely to practitioner clinical decision-making were excluded from the review. We accept that we excluded papers pertaining to the dilemmas practitioners face when making unilateral medication decisions due to situations, such as SU acute unwellness.

A strength of this review was the engagement with PG and LEG stakeholders throughout the review process. Their engagement supported our interpretations of data, ensured our findings reflected their real-world experiences and highlighted gaps in the literature. Our review identified evidence gaps in relation to the relationship between race, ethnicity, vulnerable groups and medication optimisation in SMI and in the role of peer support workers. Realist reviews are an iterative process of developing theory and CMOCs, which may then be confirmed, refuted or refined by future research, including, for example, realist evaluation. This review’s programme theory and CMOCs produced testable hypotheses for future research with SUs with SMI and community-based practitioners who serve them. In the papers included in this review, there was a lack of information on the contextual factors and mechanisms influencing clinical decision-making in challenging circumstances, such as SU acute unwellness. This was also highlighted by Martínez-Hernáez *et al*^88^ who stated that are still differences between models of concordance, described as therapeutic alliance building, and real-life practice situations.

## Conclusions

Medication optimisation is a person-centred approach that begins at time of initial diagnosis and ensures optimal information and supports are accessible to SUs, based on their needs and preferences. At the beginning of the journey and during periods of relapse, supported decision-making is necessary to overcome stigma and establish trust between SUs and practitioners. Practitioners engaging in SDM must ensure that the voice of the SU is present throughout the recovery journey in order to optimise medication.

## supplementary material

10.1136/bmjqs-2023-016615 online supplemental file 1

10.1136/bmjqs-2023-016615 online supplemental file 2

10.1136/bmjqs-2023-016615 online supplemental file 3

10.1136/bmjqs-2023-016615 online supplemental file 4

## Data Availability

All data relevant to the study are included in the article or uploaded as supplementary information.
