# Supplementary material for: A realist review of medication optimisation of community dwelling service users with serious mental illness
Source: BMJ Qual Saf. 2023 Dec 7;34(1):e016615. doi: 10.1136/bmjqs-2023-016615 (PMC11671929; doi:10.1136/bmjqs-2023-016615)
Supplement: online supplemental file 1 [file bmjqs-34-1-s001.pdf]

Supplementary File 1: MEDIATE search strategies

Main database search

MEDLINE

Host: Ovid

Data parameters: Ovid MEDLINE® ALL

Date range searched: 1946 to present (Daily update)

Date searched: 12/01/2022

Searcher: CD

Hits: n=434

1	(serious mental illness* or serious mental disorder*).ti,ab,kw.	4786
2	(severe mental illness* or severe mental disorder*).ti,ab,kw.	6762
3	SMI.ti,ab,kw.	5722
4	(schizophren* or schizoaffective or psychosis or psychotic or bipolar disorder* or personality disorder*).ti,ab,kw.	214792
5	exp *"bipolar and related disorders"/ or exp *"schizophrenia spectrum and other psychotic disorders"/ or exp *"personality disorders"/	183862
6	or/1-5	274623
7	(antipsychotic* or anti-psychotic* or psychotropic* or neuroleptic*).ti,ab,kw.	76358
8	(clozapine or olanzapine or quetiapine or risperidone or aripiprazole or haloperidol).ti,ab,kw.	43567
9	exp *Antipsychotic Agents/	79470
10	((medication or medicine*) adj1 (optimi* or manag*)).ti,ab,kw.	5678
11	((appropriate* or inappropriate*) adj2 prescri*).ti,ab,kw.	5669
12	(deprescri* OR de-prescri*).ti,ab,kw.	351
13	(polypharmacy or poly-pharmacy).ti,ab,kw.	9476
14	*Medication Therapy Management/	1596
15	*deprescriptions/	571
16	Inappropriate Prescribing/	4146
17	or/7-16	158205
18	((shared or joint or collab*) adj1 decision*).ti,ab,kw.	11704
19	*Decision Making, Shared/	764
20	((decision or communication) adj1 (aid* or support* or tool*)).ti,ab,kw.	27810
21	*decision support techniques/	12771
22	or/18-21	47905
23	((patient* or client* or service user* or person or people) adj1 (centred or centered or participation or involvement or engagement or preferen* or empower* or self-determin*)).ti,ab,kw.	60459
24	*Patient Participation/	16117
25	exp *Patient-Centered Care/	14278
26	or/23-25	79410
27	((clinician* or doctor* or physician* or psychiatrist* or psychologist or nurse* or pharmacist* or health care professional* or healthcare professional* or social care professional* or prescriber*) adj1 (patient* or	14629

	client* or service user* or family or carer*) adj1 (relations* or communic* or cooperat* or partnership* or collab*)).ti,ab,kw.	
28	therapeutic alliance.ti,ab,kw.	2919
29	*professional-family relations/ or *professional-patient relations/ or *nurse-patient relations/ or *physician-patient relations/ or *therapeutic alliance/	73250
30	or/27-29	83679
31	6 and 17 and (22 or 26 or 30)	475
32	limit 31 to english language	434

Embase

Host: Ovid

Data parameters: Embase 1974 to present

Date range searched: 1974 to present (Daily update)

Date searched: 12/01/2022

Searcher: CD

Hits: n=615

1	(serious mental illness* or serious mental disorder*).ti,ab,kw.	5844
2	(severe mental illness* or severe mental disorder*).ti,ab,kw.	9002
3	SMI.ti,ab,kw.	8497
4	(schizophren* or schizoaffective or psychosis or psychotic or bipolar disorder* or personality disorder*).ti,ab,kw.	288867
5	exp *bipolar disorder/ or exp *schizophrenia/ or exp *psychosis/ or exp *personality disorder/	232560
6	or/1-5	347444
7	(antipsychotic* or anti-psychotic* or psychotropic* or neuroleptic*).ti,ab,kw.	111542
8	(clozapine or olanzapine or quetiapine or risperidone or aripiprazole or haloperidol).ti,ab,kw.	61242
9	exp *neuroleptic agent/	129929
10	((medication or medicine*) adj1 (optimi* or manag*)).ti,ab,kw.	9631
11	((appropriate* or inappropriate*) adj2 prescri*).ti,ab,kw.	9644
12	(deprescri* OR de-prescri*).ti,ab,kw.	2065
13	(polypharmacy or poly-pharmacy).ti,ab,kw.	16197
14	*Medication Therapy Management/	4868
15	*deprescription/ or *polypharmacy/	5340
16	exp *inappropriate prescribing/	2850
17	or/7-16	238346
18	((shared or joint or collab*) adj1 decision*).ti,ab,kw.	16561
19	*shared decision making/	2939
20	((decision or communication) adj1 (aid* or support* or tool*)).ti,ab,kw.	37538
21	*decision support system/	11336
22	or/18-21	57329
23	((patient* or client* or service user* or person or people) adj1 (centred or centered or participation or involvement or engagement or preferen* or empower* or self-determin*)).ti,ab,kw.	86326
24	*Patient Participation/	10500

25	or/23-24	94070
26	((clinician* or doctor* or physician* or psychiatrist* or psychologist or nurse* or pharmacist* or health care professional* or healthcare professional* or social care professional* or prescriber*) adj1 (patient* or client* or service user* or family or carer*) adj1 (relations* or communic* or cooperat* or partnership* or collab*)).ti,ab,kw.	18113
27	therapeutic alliance.ti,ab,kw.	4206
28	exp *professional-patient relationship/	20671
29	or/26-28	41129
30	6 and 17 and (22 or 25 or 29)	668
31	limit 30 to english language	615

PsycINFO

Host: Ovid

Data parameters: PsycINFO 1806 to present

Date range searched: 1806 to present (Weekly update)

Date searched: 12/01/2022

Searcher: CD

Hits: n=286

1	(serious mental illness* or serious mental disorder*).ti,ab.	5512
2	(severe mental illness* or severe mental disorder*).ti,ab.	6876
3	SMI.ti,ab.	2493
4	(schizophren* or schizoaffective or psychosis or psychotic or bipolar disorder* or personality disorder*).ti,ab.	215283
5	exp *psychosis/ or exp *acute psychosis/ or exp *chronic psychosis/ or exp *"paranoia (psychosis)"/ or exp *schizophrenia/ or exp *personality disorders/ or exp *bipolar disorder/	159102
6	or/1-5	241238
7	(antipsychotic* or anti-psychotic* or psychotropic* or neuroleptic*).ti,ab.	51054
8	(clozapine or olanzapine or quetiapine or risperidone or aripiprazole or haloperidol).ti,ab.	25798
9	exp *neuroleptic drugs/	28609
10	((medication or medicine*) adj1 (optimi* or manag*)).ti,ab.	1893
11	((appropriate* or inappropriate*) adj2 prescri*).ti,ab.	735
12	(deprescrib* or de-prescrib*).ti,ab.	126
13	(polypharmacy or poly-pharmacy).ti,ab.	2180
14	*polypharmacy/	1005
15	or/7-14	67584
16	((shared or joint or collab*) adj1 decision*).ti,ab.	3880
17	((decision or communication) adj1 (aid* or support* or tool*)).ti,ab.	8348
18	*decision support systems/	3185
19	or/16-18	13413
20	((patient* or client* or service user* or person or people) adj1 (centred or centered or participation or involvement or engagement or preferen* or empower* or self-determin*)).ti,ab.	24559

21	*client participation/	2033
22	*patient centered care/	242
23	or/20-22	25793
24	((clinician* or doctor* or physician* or psychiatrist* or psychologist or nurse* or pharmacist* or health care professional* or healthcare professional* or social care professional* or prescriber*) adj1 (patient* or client* or service user* or family or carer*) adj1 (relations* or communic* or cooperat* or partnership* or collab*)).ti,ab.	6086
25	therapeutic alliance.ti,ab.	5475
26	*therapeutic alliance/	4507
27	or/24-26	13757
28	6 and 15 and (19 or 23 or 27)	325
29	limit 28 to english language	286

CINAHL (Cumulative Index to Nursing and Allied Health Literature)

Host: EbscoHOST

Data parameters: CINAHL 1981 onwards

Date range searched: 1981 to present (Update unknown)

Date searched: 12/01/2022

Searcher: CD

Hits: n=279

S32	S6 AND S17 AND S30	279
S31	S6 AND S17 AND S30	282
S30	S21 OR S25 OR S29	147,278
S29	S26 OR S27 OR S28	61,351
S28	(MM "Professional-Client Relations+") OR (MM "Professional-Patient Relations+") OR (MM "Professional-Family Relations")	54,797

S27	TI "therapeutic alliance" OR AB "therapeutic alliance"	1,531
S26	TI ((clinician* or doctor* or physician* or psychiatrist* or psychologist or nurse* or pharmacist* or "health care professional*" or "healthcare professional*" or "social care professional*" or prescriber*) N1 (patient* or client* or "service user*" or family or carer*) N1 (relations* or communic* or cooperat* or partnership* or collab*)) OR AB ((clinician* or doctor* or physician* or psychiatrist* or psychologist or nurse* or pharmacist* or "health care professional*" or "healthcare professional*" or "social care professional*" or prescriber*) N1 (patient* or client* or "service user*" or family or carer*) N1 (relations* or communic* or cooperat* or partnership* or collab*))	9,412
S25	S22 OR S23 OR S24	67,636
S24	(MM "Patient Centered Care")	15,994
S23	(MM "Consumer Participation")	12,845
S22	TI ((patient* or client* or "service user*" or person or people) N1 (centred or centered or participation or involvement or engagement or preferen* or empower* or self-determin*)) OR AB ((patient* or client* or "service user*" or person or people) N1 (centred or centered or participation or involvement or engagement or preferen* or empower* or self-determin*))	49,364
S21	S18 OR S19 OR S20	28,396
S20	TI ((decision or communication) N1 (aid* or support* or tool*)) OR AB ((decision or communication) N1 (aid* or support* or tool*))	18,961
S19	(MM "Decision Making, Shared") OR (MM "Decision Support Techniques")	5,267

S18	TI ((shared or joint or collab*) N1 decision*) OR AB ((shared or joint or collab*) N1 decision*)	7,568
S17	S7 OR S8 OR S9 OR S10 OR S11 OR S12 OR S13 OR S14 OR S15 OR S16	42,399
S16	(MM "Inappropriate Prescribing")	2,032
S15	(MM "Polypharmacy+")	2,311
S14	(MM "Medication Management")	642
S13	TI (polypharmacy or poly-pharmacy) OR AB (polypharmacy or poly-pharmacy)	4,234
S12	TI (deprescri* OR de-prescri*) OR AB (deprescri* OR de-prescri*)	1,054
S11	TI ((appropriate* or inappropriate*) N2 prescri*) OR AB ((appropriate* or inappropriate*) N2 prescri*)	3,228
S10	TI ((medication or medicine*) N1 (optimi* or manag*)) OR AB ((medication or medicine*) N1 (optimi* or manag*))	6,678
S9	(MM "Antipsychotic Agents+")	14,028

S8	TI (clozapine or olanzapine or quetiapine or risperidone or aripiprazole or haloperidol) OR AB (clozapine or olanzapine or quetiapine or risperidone or aripiprazole or haloperidol)	8,735
S7	TI (antipsychotic* OR anti-psychotic* OR psychotropic* OR neuroleptic*) OR AB (antipsychotic* OR anti-psychotic* OR psychotropic* OR neuroleptic*)	18,990
S6	S1 OR S2 OR S3 OR S4 OR S5	141,494
S5	(MM "Psychotic Disorders+") OR (MM "Personality Disorders+")	117,057
S4	TI (schizophren* OR schizoaffective OR psychosis OR psychotic OR "bipolar disorder*" OR "personality disorder*") OR AB (schizophren* OR schizoaffective OR psychosis OR psychotic OR "bipolar disorder*" OR "personality disorder*")	57,164
S3	TI SMI OR AB SMI	2,222
S2	TI ("severe mental illness*" OR "severe mental disorder*") OR AB ("severe mental illness*" OR "severe mental disorder*")	3,684
S1	TI ("serious mental illness*" OR "serious mental disorder*") OR AB ("serious mental illness*" OR "serious mental disorder*")	3,376

Cochrane Library

Host: Cochrane Library

Data parameters: CDSR, Protocols, CENTRAL (trials), Editorials, Special Collections, Clinical Answers

Date range searched: No limit

Date searched: 12/01/2022

Searcher: CD

Hits: n=164

#1	("serious mental illness*" or "serious mental disorder*"):ti,ab,kw	739
#2	("severe mental illness*" or "severe mental disorder*"):ti,ab,kw	1005
#3	(SMI):ti,ab,kw	839
#4	(schizophren* or schizoaffective or psychosis or psychotic or "bipolar disorder*" or "personality disorder*"):ti,ab,kw	30237
#5	MeSH descriptor: [Bipolar and Related Disorders] explode all trees	2813
#6	MeSH descriptor: [Schizophrenia Spectrum and Other Psychotic Disorders] explode all trees	9647
#7	MeSH descriptor: [Personality Disorders] explode all trees	1458
#8	#1 OR #2 OR #3 OR #4 OR #5 OR #6 OR #7	31816
#9	*(antipsychotic* or anti-psychotic* or psychotropic* or neuroleptic*):ti,ab,kw*	14078
#10	(clozapine or olanzapine or quetiapine or risperidone or aripiprazole or haloperidol):ti,ab,kw	10945
#11	MeSH descriptor: [Antipsychotic Agents] explode all trees	4774
#12	(((medication or medicine*) near/1 (optimi* or manag*))):ti,ab,kw	1166
#13	(((appropriate* or inappropriate*) near/2 prescri*)):ti,ab,kw	842
#14	((deprescri* OR de-prescri*)):ti,ab,kw	301
#15	((polypharmacy or poly-pharmacy)):ti,ab,kw	1151
#16	MeSH descriptor: [undefined] explode all trees	0
#17	MeSH descriptor: [Deprescriptions] this term only	43
#18	MeSH descriptor: [Inappropriate Prescribing] this term only	169
#19	#9 OR #10 OR #11 OR #12 OR #13 OR #14 OR #15 OR #16 OR #17 OR #18	22129
#20	(((shared or joint or collab*) near/1 decision*)):ti,ab,kw	1730
#21	MeSH descriptor: [Decision Making, Shared] this term only	65
#22	(((decision or communication) near/1 (aid* or support* or tool*))):ti,ab,kw	5107
#23	MeSH descriptor: [Decision Support Techniques] this term only	881
#24	#20 OR #21 OR #22 OR #23	6275
#25	(((patient* or client* or "service user*" or person or people) near/1 (centred or centered or participation or involvement or engagement or preferen* or empower* or self-determin*))):ti,ab,kw	14309
#26	MeSH descriptor: [Patient Participation] this term only	1489
#27	MeSH descriptor: [Patient-Centered Care] explode all trees	813
#28	#25 OR #26 OR #27	14429
#29	(((clinician* or doctor* or physician* or psychiatrist* or psychologist or nurse* or pharmacist* or "health care professional*" or "healthcare professional*" or "social care professional*" or prescriber*) near/1 (patient* or client* or "service user*" or family or carer*) near/1 (relations* or communic* or cooperat* or partnership* or collab*))):ti,ab,kw	2791
#30	("therapeutic alliance"):ti,ab,kw	787
#31	MeSH descriptor: [undefined] explode all trees	0
#32	MeSH descriptor: [Professional-Patient Relations] this term only	792
#33	MeSH descriptor: [Nurse-Patient Relations] this term only	397
#34	MeSH descriptor: [Physician-Patient Relations] this term only	1456
#35	MeSH descriptor: [Therapeutic Alliance] this term only	53
#36	#29 OR #30 OR #31 OR #32 OR #33 OR #34 OR #35	4205
#37	#24 OR #28 OR #36	22884
#38	#8 AND #19 AND #37	164

Scopus

Host: Scopus.com

Data parameters: n/a

Date range searched: No limit

Date searched: 13/01/2022

Searcher: CD

Hits: n=368

Full string	(TITLE-ABS ("serious mental illness*" OR "serious mental disorder*" OR "severe mental illness*" OR "severe mental disorder*" OR smi OR schizophren* OR schizoaffective OR psychosis OR psychotic OR "bipolar disorder*" OR "personality disorder*")) AND ((TITLE-ABS (antipsychotic OR anti-psychotic* OR psychotropic* OR neuroleptic* OR clozapine OR olanzapine OR quetiapine OR risperidone OR aripiprazole OR haloperidol)) OR (TITLE-ABS ((medication OR medicine) W/1 (optimi* OR manag*))) OR (TITLE-ABS ((appropriate* OR inappropriate*) W/2 (prescri*))) OR (TITLE-ABS (deprescri* OR de-prescri*)) OR (TITLE-ABS (polypharmacy OR poly-pharmacy))) AND ((TITLE-ABS ((shared OR joint OR collab*) W/1 decision*)) OR (TITLE-ABS ((decision OR communication) W/1 (aid* OR support* OR tool*))) OR (TITLE-ABS ((patient* OR client* OR "service user*" OR person OR people) W/1 (centred OR centered OR participation OR involvement OR engagement OR preferen* OR empower* OR self-determin*))) OR (TITLE-ABS ((clinician* OR doctor* OR physician* OR psychiatrist* OR psychologist OR nurse* OR pharmacist* OR "health care professional*" OR "healthcare professional*" OR "social care professional*" OR prescriber*) W/1 (patient* OR client* OR "service user*" OR family OR carer*) W/1 (relations* OR communic* OR cooperat* OR partnership* OR collab*))) OR (TITLE-ABS ("therapeutic alliance"))) AND (LIMIT-TO (LANGUAGE , "English"))	368

Web of Science (Core)

Host: Web of Science (Clarivate Analytics)

Data parameters: SCIE, SSCI, SHCI, ESCI, CPCI, BKCI indexes

Date range searched: Unknown

Date searched: 13/01/2022

Searcher: CD

Hits: n=368

1	TI=(("Serious mental illness*" OR "serious mental disorder*" OR "severe mental illness*" OR "severe mental disorder*" OR SMI OR	388022

	schizophren* OR schizoaffective OR psychosis OR psychotic OR "bipolar disorder*" OR "personality disorder*")) OR AB=(("Serious mental illness*" OR "serious mental disorder*" OR "severe mental illness*" OR "severe mental disorder*" OR SMI OR schizophren* OR schizoaffective OR psychosis OR psychotic OR "bipolar disorder*" OR "personality disorder*"))	
2	TI=(antipsychotic* OR anti-psychotic* OR psychotropic* OR neuroleptic* OR clozapine OR olanzapine OR quetiapine OR risperidone OR aripiprazole OR haloperidol) OR AB=(antipsychotic* OR anti-psychotic* OR psychotropic* OR neuroleptic* OR clozapine OR olanzapine OR quetiapine OR risperidone OR aripiprazole OR haloperidol)	149751
3	TI=(((medication OR Medicine*) Near/1 (optimi* OR manag*))) OR AB=(((medication OR Medicine*) Near/1 (optimi* OR manag*)))	14929
4	TI=(((appropriate* OR inappropriate*) near/2 prescri*)) OR AB=(((appropriate* OR inappropriate*) near/2 prescri*))	8944
5	TI=((deprescri* OR de-prescri*)) OR AB=((deprescri* OR de-prescri*))	2905
6	TI=((polypharmacy OR poly-pharmacy)) OR AB=((polypharmacy OR poly-pharmacy))	11643
7	#2 OR #3 OR #4 OR #5 OR #6	183623
8	TI=(((shared OR joint OR collab*) near/1 decision*)) OR AB=(((shared OR joint OR collab*) near/1 decision*))	19711
9	TI=((decision OR communication) near/1 (aid* or support* or tool*)) OR AB=((decision OR communication) near/1 (aid* or support* or tool*))	154797
10	TI=((patient* or client* or "service user*" or person or people) near/1 (centred or centered or participation or involvement or engagement or preferen* or empower* or self-determin*)) OR AB=((patient* or client* or "service user*" or person or people) near/1 (centred or centered or participation or involvement or engagement or preferen* or empower* or self-determin*))	138302
11	TI=(((clinician* or doctor* or physician* or psychiatrist* or psychologist or nurse* or pharmacist* or "health care professional*" or "healthcare professional*" or "social care professional*" or prescriber*) near/1 (patient* or client* or "service user*" or family or carer*)) near/1 (relations* or communic* or cooperat* or partnership* or collab*)) OR AB=(((clinician* or doctor* or physician* or psychiatrist* or psychologist or nurse* or pharmacist* or "health care professional*" or "healthcare professional*" or "social care professional*" or prescriber*) near/1 (patient* or client* or "service user*" or family or carer*)) near/1 (relations* or communic* or cooperat* or partnership* or collab*))	21189
12	TI=("therapeutic alliance") OR AB=("therapeutic alliance")	4149
13	#12 OR #11 OR #10 OR #9 OR #8	328533
14	#13 AND #7 AND #1	483
15	#13 AND #7 AND #1 and Web of Science Core Collection (Database) and English (Languages)	368

Sociological Abstracts

Host: Proquest

Data parameters: 1952 to present (update unknown)

Date range searched: 1952 to present

Date searched: 13/01/2022

Searcher: CD

Hits: n=8

Full string	(noft("serious mental illness*" OR "serious mental disorder*" OR "severe mental illness*" OR "severe mental disorder*" OR SMI) OR noft(schizophren* OR schizoaffective OR psychosis OR psychotic OR "bipolar disorder*" OR "personality disorder*")) AND (noft(antipsychotic* OR anti-psychotic* OR psychotropic* OR neuroleptic* OR clozapine OR olanzapine OR quetiapine or risperidone or aripiprazole or haloperidol) OR noft((medication or medicine*) N1 (optimi* or manag*)) OR noft((medication OR medicine*) NEAR/1 (optimi* OR manag*)) OR noft((appropriate* OR inappropriate*) N/2 (prescri*)) OR noft(deprescri* OR de-prescri*) OR noft(polypharmacy OR poly-pharmacy)) AND (noft((shared OR joint OR collab*) N/1 decision*) OR noft((decision OR communication) N/1 (aid* OR support* OR tool*)) OR noft((patient* or client* or ("service user" OR "service users") or person or people) N/1 (centred or centered or participation or involvement or engagement or preferen* or empower* or self-determin*)) OR noft((clinician* or doctor* or physician* or psychiatrist* or psychologist or nurse* or pharmacist* or "health care professional*" or ("healthcare professionals") or "social care professional*" or prescriber*) N/1 (patient* or client* or ("service user" OR "service users") or family or carer*) N/1 (relations* or communic* or cooperat* or partnership* or collab*)) OR noft("therapeutic alliance"))Limits applied	8

Additional search 1: Internet use for health information

MEDLINE

Host: Ovid

Data parameters: Ovid MEDLINE® ALL

Date range searched: 1946 to present (Daily update)

Date searched: 16/06/2022

Searcher: CD

Hits: n=51

1	(serious mental illness* or serious mental disorder*).ti.	1985
2	(severe mental illness* or severe mental disorder*).ti.	2861
3	SMI.ti.	274
4	(schizophren* or schizoaffective or psychosis or psychotic or bipolar disorder* or personality disorder*).ti.	135488

5	exp *"bipolar and related disorders"/ or exp *"schizophrenia spectrum and other psychotic disorders"/ or exp *"personality disorders"/	187573
6	or/1-5	213216
7	(internet or online or digital or web* or social media).ti.	131598
8	exp *Internet/	51669
9	or/7-8	150150
10	*Access to Information/	3801
11	*Information Seeking Behavior/	1981
12	*Patient Education as Topic/	40821
13	exp *Health Education/	152838
14	or/10-13	157860
15	6 and 9 and 14	51

PsycINFO

Host: Ovid

Data parameters: PsycINFO 1806 to present

Date range searched: 1806 to present (Weekly update)

Date searched: 16/06/2022

Searcher: CD

Hits: n=8

1	(serious mental illness* or serious mental disorder*).ti.	2052
2	(severe mental illness* or severe mental disorder*).ti.	2622
3	SMI.ti.	66
4	(schizophren* or schizoaffective or psychosis or psychotic or bipolar disorder* or personality disorder*).ti.	122262
5	exp *psychosis/ or exp *acute psychosis/ or exp *chronic psychosis/ or exp *"paranoia (psychosis)"/ or exp *schizophrenia/ or exp *personality disorders/ or exp *bipolar disorder/	161261
6	or/1-5	177601
7	(internet or online or digital or web* or social media).ti.	61281
8	exp *Internet/	23697
9	or/7-8	68872
10	exp *health information/	1783
11	exp *information seeking/ or *computer searching/	7324
12	exp *health literacy/	3337
13	*health knowledge/	6824
14	or/10-13	18211
15	6 and 9 and 14	8

CINAHL

Host: EbscoHOST

Data parameters: CINAHL 1981 onwards

Date range searched: 1981 to present (Update unknown)

Date searched: 16/06/2022

Searcher: CD

Hits: n=42

S17	S6 AND S9 AND S16	42
S16	S10 OR S11 OR S12 OR S13 OR S14 OR S15	71,217
S15	(MM "Health Knowledge")	16,703
S14	(MM "Health Literacy")	4,086
S13	(MM "Health Education")	15,410
S12	(MM "Patient Education")	25,236
S11	(MM "Information Seeking Behavior")	2,677
S10	(MM "Access to Information+")	10,430
S9	S7 OR S8	115,736

S8	(MM "Internet+")	62,333
S7	TI internet or online or digital or web* or "social media")	86,218
S6	S1 OR S2 OR S3 OR S4 OR S5	129,725
S5	(MM "Psychotic Disorders+") OR (MM "Personality Disorders+")	120,415
S4	TI schizophren* or schizoaffective or psychosis or psychotic or "bipolar disorder*" or "personality disorder*")	39,522
S3	TI SMI	233
S2	TI "severe mental illness*" OR "severe mental disorder*"	1,815

S1	TI "serious mental illness*" or "serious mental disorder*")	1,637

Additional search 2: Peer support for health information

MEDLINE

Host: Ovid

Data parameters: Ovid MEDLINE® ALL

Date range searched: 1946 to present (Daily update)

Date searched: 20/06/2022

Searcher: CD

Hits: n=17

1	(serious mental illness* or serious mental disorder*).ti.	1985
2	(severe mental illness* or severe mental disorder*).ti.	2864
3	SMi.ti.	274
4	(schizophren* or schizoaffective or psychosis or psychotic or bipolar disorder* or personality disorder*).ti.	135530
5	exp *"bipolar and related disorders"/ or exp *"schizophrenia spectrum and other psychotic disorders"/ or exp *"personality disorders"/	187648
6	1 or 2 or 3 or 4 or 5	213290
7	(peer* adj1 (support* or group* or led)).ti.	2482
8	(expert patient* or "expert* by experience*" or lay expert*).ti.	166
9	exp *peer group/	10452
10	7 or 8 or 9	11809
11	*Access to Information/	3801
12	*Information Seeking Behavior/	1982
13	*Patient Education as Topic/	40825
14	exp *Health Education/	152860
15	11 or 12 or 13 or 14	157883
16	6 and 10 and 15	17

PsycINFO

Host: Ovid

Data parameters: PsycINFO 1806 to present

Date range searched: 1806 to present (Weekly update)

Date searched: 20/06/2022

Searcher: CD

Hits: n=0

1	(serious mental illness* or serious mental disorder*).ti.	2052
2	(severe mental illness* or severe mental disorder*).ti.	2622
3	SMi.ti.	66
4	(schizophren* or schizoaffective or psychosis or psychotic or bipolar disorder* or personality disorder*).ti.	122262
5	exp *"bipolar and related disorders"/ or exp *"schizophrenia spectrum and other psychotic disorders"/ or exp *"personality disorders"/	25212
6	1 or 2 or 3 or 4 or 5	138879
7	(peer* adj1 (support* or group* or led)).ti.	2562
8	(expert patient* or "expert* by experience*" or lay expert*).ti.	92
9	exp *peer relations/ or *peer counseling/ or *peers/	22823
10	7 or 8 or 9	23745
11	exp *health information/	1783
12	exp *information seeking/	5911
13	exp *health literacy/	3337
14	*health knowledge/	6824
15	11 or 12 or 13 or 14	16848
16	6 and 10 and 15	0

CINAHL

Host: EbscoHOST

Data parameters: CINAHL 1981 onwards

Date range searched: 1981 to present (Update unknown)

Date searched: 20/06/2022

Searcher: CD

Hits: n=4

S19	S11 AND S18	4
S18	S12 OR S13 OR S14 OR S15 OR S16 OR S17	71,233
S17	(MM "Health Knowledge")	16,707

S16	(MM "Health Literacy")	4,089
S15	(MM "Health Education")	15,413
S14	(MM "Patient Education")	25,241
S13	(MM "Information Seeking Behavior")	2,677
S12	(MM "Access to Information+")	10,434
S11	S6 AND S10	203
S10	S7 OR S8 OR S9	8,777
S9	(MM "Peer Counseling") OR (MM "Peer Group")	7,678
S8	TI "expert patient*" OR "expert* by experience* OR "lay expert*")	137
S7	TI peer N1 (support* or group* or led)	2,374

S6	S1 OR S2 OR S3 OR S4 OR S5	144,416
S5	(MM "Psychotic Disorders+") OR (MM "Personality Disorders+")	120,459
S4	TI schizophren* or schizoaffective or psychosis or psychotic or "bipolar disorder*" or "personality disorder*"	67,417
S3	TI SMI	233
S2	TI "severe mental illness*" OR "severe mental disorder*"	2,157
S1	TI "serious mental illness*" or "serious mental disorder*")	1,779
