# Supplementary material for: A realist review of medication optimisation of community dwelling service users with serious mental illness
Source: BMJ Qual Saf. 2023 Dec 7;34(1):e016615. doi: 10.1136/bmjqs-2023-016615 (PMC11671929; doi:10.1136/bmjqs-2023-016615)
Supplement: online supplemental file 3 [file bmjqs-34-1-s003.pdf]

Supplementary File 3: MEDIATE CMOC Coding Framework

Overarching Conceptual Theme	Parent Node	Child Node	Notes for researchers
Decision Making	Coercion:		Pressure to follow advice against own wishes/may include threat of Mental Health Act
	Independent decision-making		
		By provider	often when the provider deems the client incapable of making rational decisions
		By client	includes autonomy, control, empowerment over own decisions
	Shared decision-making:		evidence and options are discussed between client and provider-client's input is equally valued and considered by the provider
	Decisional factors-		often manifest during conflict/dilemma, new decision or change in condition
		Available information:	includes insufficient or sufficient info or access to info
		Family, peer roles/accountabilities	may be positive or negative
		Cultural and social influences	
		Stigma	stigma against SMI
		Other contextual considerations,	such as time and space for consultations, team-based care/inter-disciplinary approaches

		Client insight or personal awareness-user/capacity/unwell	
Therapeutic Alliance	relational factors		
		Patient approaches and perceptions	motivational interviewing, informed consent
		Provider approaches and perceptions	
		Strategies/interventions:	
Med Management Interventions	Positive experiences and outcomes		
	Negative experiences and outcomes		
	Orals (+/- experiences)		Types of medications
	Injectables [LAIs] (+/- experiences)		
	Theories		E.g., Example: Common sense or self-regulation theory in Pinfold<br>Stages of SDM and their barriers in Grunwald & Thompson<br>Personal identity formation
	Background Information		with conceptual/operational definitions of key terms and stats

Deleted: Therapeutic alliance/encounter/relationship-
