# Supplementary material for: A realist review of medication optimisation of community dwelling service users with serious mental illness
Source: BMJ Qual Saf. 2023 Dec 7;34(1):e016615. doi: 10.1136/bmjqs-2023-016615 (PMC11671929; doi:10.1136/bmjqs-2023-016615)
Supplement: online supplemental file 4 [file bmjqs-34-1-s004.pdf]

Supplementary File 4: MEDIATE CMOC data appendix

Each CMOC developed during the review is presented below with the underpinning data (extracted text).

Highlighted portions of text reflect researcher perceived level of importance at time of initial CMOC composition

CMOC 1 ('First Contact')

When an individual with SMI (Severe Mental Illness) is first diagnosed, is medicated and experiences coercive, dehumanizing experiences from practitioners (C), this often derails the development of trusting therapeutic alliances (O) because of feelings of powerlessness (M) and stigmatization (M).

CMOC based on data extracted from 9 documents.

Document (author, date)	Extracted data	Subset of literature
(Carrick et al., 2004)	The ability to function was impacted by how people felt in themselves (e.g. `I just feel really well and healthy most of the time' [Rebecca]; `It feels dead. I feel dead' [Brenda]. Appearance to the outside **world or embarrassment at how others perceived them** was of obvious concern and widely reported.[…]<br><br>In summary, a person's situation is evaluated easily in terms of the goal of Well-being. This goal is personally defined, but rests on function, feeling and appearance to the outside world. Treatment is viewed negatively if it does not increase Well-being (i.e., if it does not reduce symptoms suffciently and/or it causes side effects individuals consider significant).	Pre-2014
(Maj et al., 2021)	The view that the management of a patient with schizophrenia (or "psychosis") should be personalized is endorsed by the vast majority of clinicians, but the awareness that this would require a comprehensive assessment of the patient, beyond the mere diagnosis, is not equally shared, and personalization of management is actually lacking or inadequate in most clinical contexts worldwide. […] The verified presence of internalized stigma may have significant implications for the formulation of the management plan. **Stigmatizing contacts with health professionals can worsen internalized stigma**, and therefore interventions to reduce stigma among health care staff will contribute to reduction in internalized stigma.	Post-2014 4*
(Bolden et al., 2019)	At the same time, psychiatrists tend to steer the treatment discussion towards a particular outcome. For example, psychiatrist may apply **pressure on their clients by eliciting a commitment from them or direct them towards a**	Post-2014 5*

	**particular treatment by marking it as a best option** (Quirk et al. 2012). […] In psychiatry, and especially in long-term psychiatric care, patients may have opinions about how well their medications are working for them, both therapeutically and in terms of side effects. However, **their opinions may not be seen as legitimate by providers** since patients lack professional authority to judge medication effectiveness and they might not have insight into their illness (Chong et al. 2013). Given the delicacy involved in advocating for particular medical treatments, it is not surprising that, across medical contexts, patients rarely request a treatment explicitly.	
(Ehrlich & Dannapfel, 2017)	However, when health professionals did not listen to participants, the relationship was experienced as being **unequal, with the professional holding the power. Often, participants did not have the necessary skills to speak up, and rather than being active participants in their health care, became passive recipients.** Thus, rather than gaining critical skills of mastering living with mental illness and managing the consequences of poor physical health, participants felt mastered by health professionals:<br>Sometimes I think GPs get so full of themselves they forget to actually listen to the patient, and that people actually do know their bodies. Anyway, you just go along, what can you do? (ID002; woman, 35 years). However, participants wanted to be involved in their health, share<br>decisions and have the legitimacy to question the proposed care and not be treated paternalistically	Post-2014<br>5*
(Grünwald & Thompson, 2021)<br>('Restarting the conversation')	As a **result of this stigma**, SUs report that they are not listened to and that clinicians make assumptions about their capabilities to understand their treatment options and be included in conversations and decisions about their own care (Lester et al., 2003).	Post-2014<br>5*
(Grunwald et al., 2021)<br>(The role of hope and trust)	Managing these uncertainties together requires a trusting relationship between GP and SU [60, 61]. Any history of coercion or sectioning under the Mental Health Act can make developing and maintaining trust more difficult, but a **trusting relationship is key to shared decision making** [29, 82]. […]	Post-2014<br>5*
(Martinez-Hernaez et al., 2020)	The patients' awareness of suffering included a set of discomforts arising from the disorder, but mainly from the adverse effects of medication, their lived experiences in psychiatric admissions and the social consequences of their mental condition: **stigma and marginalisation** in the social arena, among others. […] However, they continued to disagree with the perception of professionals, mainly when **they considered they were not being well cared for, or because of hierarchical communication models that made them feel judged or undervalued**. In some cases (e.g. Male Patient 35), they specified that professionals' insistence on awareness of the disorder was a pretext for overmedication and coercive practices and reflected a disregard for other aspects of their lives that caused them suffering, such as **the diagnosis itself operating as a semantic and existential ascription which, by naming the affliction, acted as a barrier to full citizenship**. They also considered that this clinical insistence on the disorder was used to remind them of their sickness, fostering what we can define here as the "total patient" effect or the reduction	Post-2014<br>5*

of the affected person to a diagnostic category. As one of the participants stated, “People usually think that when a person suffers from a disorder, they have a permanent disorder. They don't. A person who has paranoid schizophrenia is not hearing voices every day” (Male Patient 7). According to those affected, this reductive perception was the main cause of professionals' and caregivers' lack of awareness of the patient's “real” suffering and its multiple sources. […]

The patients recognised that when they are in relapse, optimal clinical communication is not easily achieved. Nevertheless, even in these cases they insisted that **the clinical response should not be one of coercive measures and high doses of medication, but dialogue to help them accept and manage their suffering**, […]

The patients highlighted a social and clinical failure to recognise them as individuals with autonomy and agency, either in their decisions regarding medical treatments or in the broad spectrum of their lives, since the conflicts with medication become a synecdoche for their existence and mobilise morals worlds that have profound effects on the sense of self. […]

Caregivers' perceptions of those affected as “vegetables”, a “piece of furniture” are expressions of this structural coercion. **It is also present in professional attitudes such as overmedication and the kind of clinical treatment that objectifies patients and transforms them into nosologies**. This de-subjectivation is precisely what the patients in our study challenged when they contrasted the trato with the tratamiento. While the former expresses the hope that they will be recognised as subjects, the latter evokes the idea of a permanent or “total patient” in need of total therapy. […]

Motivated by Goffman (2017) classic concept of the total institution as places that absorb the set of behaviours of the individuals who live there together, such as the asylums prior to psychiatric reform, here we propose the idea of the “total patient” to express the reduction of the self to its condition as patient, in such a way that this identity absorbs the affected person's life in its totality. In this vein, it has parallels with other notions such as dehumanisation (Kontio et al., 2012), or desubjectivation (Verbeke et al., 2019). The identity is reduced to a very specific one in which the damaged self is perceived as a psychopathological island requiring total therapeutic intervention or total therapy. The phenomena of the total patient and total therapy clearly limit the possibility of dialogue. Patients see themselves as “zombies” induced by antipsychotics (see also Flore et al., 2019; Werremeyer et al., 2017; Morrison and MeehanStomski, 2015); their caregivers see them as a “**piece of furniture” without autonomy, and professionals see them primarily as “disorders”,** thus encouraging the primacy of nosology over illness narrative; bureaucratic pigeonholing over biography; technical treatment over human needs.

(Yeisen et al., 2017)	It seems that the **first contact with staff has a considerable impact on alliance and trust** during the treatment process to follow, which in turn affects adherence to AP, especially after discharge. This supports findings from another study, that the reception of patients by medical staff has crucial impact on adherence, regardless of legal status of admission [42]. Also it might explain why patients in our study who felt that **the staff violated their rights to freedom and authority to choose**, chose to discontinue their medication after discharge as a way of trying to regain control over their own life, regardless of legal status when admitted.	Post-2014<br>5*
(Younas et al., 2016)	SDM could help **service users feel more valued and respected, and work towards removing some of the stigma that is associated with mental health**.<br>**It's huge stigma all around… so if you treat them like every other human being… they're going to feel valued and respected definitely** …there's definite improvement, they feel at the centre of their care…they will respect you for giving them that rather than being domineering and telling them…. I know better than you. (In02) […]<br><br>**A strong, trusting relationship, with health care professionals and service users both accepting an active role, is essential to the success, or otherwise of SDM** [47].	Post-2014<br>5*

CMOC 2 ('Relief')

When an individual with SMI is first diagnosed and is medicated, validation and normalization of their condition by a respectful, supportive practitioner (C) results in increased relief, hope and optimism (O) due to decreased stigmatization of living with SMI (M) and increased reassurance (M) that they have a treatable condition.

CMOC based on data extracted from 6 documents.

**Document (author, date)**	**Extracted data**	**Subset of literature**
(Green et al., 2008)	A strong sense of hope arising from clinicians' positive attitudes and confidence about recovery and about treatment and available alternatives. This included powerful messages indicating clinicians were confident that a treatment would be found that would work, even when patients were having difficulties with current treatments.	Pre-2014
(Kaar et al., 2019)	Our study found that negative experiences with medication were more prominent among responses than positive ones, and **these experiences tend to stay with the patient over many years** and affect future decision making. […]<br>This highlights the need for psychiatrists to take a detailed medication history with a focus on past experiences of medication and to be explicit in their discussion with patients about the benefits and adverse effects of an	Post-2014<br>4*

	antipsychotic and, whenever possible, have a conversation about the comparative risks and benefits of different antipsychotic options [46].	
(Maj et al., 2021)	**: Internalized stigma has several key implications** for clinical practice. First, mental health practitioners need to recognize that internalized stigma among patients with a diagnosis of primary psychosis, in particular schizophrenia, is likely to be common and may be disabling. It is therefore necessary to ask patients directly about their understanding of their diagnosis of psychosis and their views about the implications of having such a condition. This will often lead to a detailed discussion to help the patient correct common misunderstandings, for example that psychosis is always a chronic and progressively disabling condition, or that psychosis means never being able to work or marry. Such discussions are often also necessary with family members to convey a **realistic prospect of recovery from a psychotic episode,** with an emphasis upon supporting advocacy, self-esteem and empowerment31	Post-2014<br>4*
(Geyt et al., 2017)	The majority of **participants recalled initial relief, hope, and optimism when told by their clinician that their experiences were treatable** with medication, as captured in the following statement:<br>I thought . . . "something can be done . . . it's not just me being a freak, this is a thing that people have and there's a thing for it." I had a very sort of reductionist attitude really in many ways. I thought, "well my brain doesn't work, just as somebody's heart might not work, so they go on, I don't know, beta-blockers." Receiving professional explanations of psychosis and treatment gave many participants a way **to make sense of their experiences,** which fitted with cultural expectations of illness and cure and was **both normalizing and validating**	Post-2014<br>5*
Martinez-Hernaez (2020)	(Martinez-Hernaez et al., 2020)Mental health workers agreed with patients that it is difficult for a person, especially if they are young and do not experience their problem as a dysfunction, to accept they have a mental disorder which requires continuous or lifelong use of medication that will affect many areas of their life: social relationships, employment, and expectations for their future. Nevertheless, they considered **awareness of the disorder to be the touchstone for treatment success.**	Post-2014<br>5*
(Weiden, 2016)	Most patients will **find it reassuring when** their clinicians provide guidance as to how they can feel better, even if that guidance is not immediately accepted.[46] Differences of opinion that are respected are easier to accept than when verbalizing those differences leaves someone feeling chastised. Clinicians can learn that listening and acknowledging feelings does not represent colluding or abandoning the process of making strong recommendations with conviction.	Post-2014<br>5*

CMOC 3 ('Dismissal')

When an individual with SMI on medications realizes practitioners are withholding medication information, and/or excluding, ignoring or dismissing them from medication decisions (C), they are apt to withdraw from the practitioner relationship and make their own medication decisions (O), due to mistrust (M) in the practitioner's interest in them and their need for more control (M) over decisions affecting their lives

CMOC based on data extracted from 20 documents.

Document (author, date)	Extracted data	Subset of literature
(Opler et al., 2004)	Lin and Smith2 present evidence that medication noncompliance is particularly high among immigrants and that this is at least partly because doctors and patients have differing views regarding the nature of distress as well as its causes and cures. Lin and Smith describe how, among Chinese, a number of disorders, including anxiety, are seen as emanating from an imbalance between cold or submissive processes (ying) and hot or active processes (yang). They describe how this can lead to a greater likelihood of anxiolytic noncompliance if a pill is red, given the culturally based belief that red is associated with exacerbation of active processes.<br>In this paper, we first describe how we came to realize that noncompliance with antipsychotics in Hispanic outpatients was due primarily to differing concepts of illness, of medication, and of medication side effects between ourselves and our patients. Second, we describe how, by learning to respect and address our patients' views, we were able to offer them real treatment. In retrospect, before we came to adequately appreciate our patients' perspective and its implications, not only was our clinical expertise of little benefit to these patients, but it was, in fact, interfering with their treatment. In many cases, our initial treatment choices probably caused more rather than less distress for our patients. Fortunately, we were able to acquire culturally responsive clinical insights and to make corrections in our prescribing practices based on these insights, leading to improved medication compliance and clinical status in our patients. […]<br><br>What we came to realize was that, even though the staff of the Tavares Clinic spoke fluent Spanish and was culturally knowledgeable, the patients and psychiatrists were in cultural conflict. […]<br>Patients, on the other hand, were coming to the Tavares Clinic to be treated not for hallucinations and delusions, but for nerviosismo. The patients concluded that, while the doctors at the clinic were well meaning, they obviously did not know how to treat nerviosismo. […]<br>While our patients did need treatment for their psychotic symptoms, it was clear that we needed to develop culturally informed treatment plans, not only for psychosocial interventions but also for pharmacotherapy, that were tailored to the needs of this specific patient population […]	Pre-2014

	We provided ongoing education, both individually and in group settings, regarding reasons for taking medication, possible side effects that could occur, and methods that could be used to alleviate many of these side effects. We also created a “Medication Calendar” that listed what medications to take and frequency and dose. Visual aids were taped to these “Medication Calendars” for our illiterate patients. We reviewed side effects periodically.[…]<br><br>The result of the changes in procedures outlined above was that, soon after their first contact with a psychiatrist, usually on the same day, patients experienced at least some relief from their nerviosismo. We observed an increase in patient retention and a decrease in noncompliance with antipsychotic medications. […]<br><br>We had used our knowledge of cultural differences to become a Hispanic mental health clinic, but not until we understood our population more thoroughly did this become more than just a change in name. Our patients had taught us the importance of considering cultural factors, even in something so seemingly straightforward as prescribing medication, and of being sensitive to the complex ways in which our own culturally based assumptions could undermine our attempts to provide effective treatment.[…]<br>The lesson to be learned here is that, in standard clinical practice, psychiatrists do not routinely explain the full spectrum of possible side effects to patients. Rather, they tend to wait until such side effects are mentioned by patients before addressing them. Given that some side effects may have particular significance for members of certain cultural groups, it is recommended that clinicians have an in-depth discussion of possible side effects with patients and assure them that there are treatment strategies (e.g., amantadine for neuroendocrine side effects) that can alleviate some of these side effects. Such discussions can improve patients’ confidence in the practitioner and increase medication compliance.	
Delman et al (2015)	(Delman et al., 2015)When residents reported that the psychiatrist was a **particularly dismissive person** or the client remained nervous despite meeting preparation support, staff might attend the meeting with the client, and if necessary act as an advocate. In one case, a client informed his case manager that he was having a difficult side effect and his psychiatrist didn’t seem interested in discussing it. The case manager offered to drive him to the next appointment: But by the time we got there I was a wreck; he offered to come up with me and I was for that. At the meeting, he was really insistent that I was suffering and there really should be some change in medication. There was tension in the room, but there didn’t seem to be any disagreement. And the medication was changed. I also felt I could be more honest with GH because of the case manager’s support, and that perhaps GH would take my concerns more seriously in the future. […]<br>**Many of the respondents described relationships with previous psychiatrists who resisted or showed a deep disinterest in client participation. In general, resistant psychiatrists were described as poor listeners, rigid, careless, and/or inaccessible.** As one person noted: “He wouldn’t listen to anything. He didn’t do anything. With Depakote,	Post-2014<br>4*

	when I needed my blood test, he never got my blood test. He never rescheduled my appointments, he just didn't do ANYTHING for me**."** **Some psychiatrists were seen as having a very rigid approach to meetings, leaving very little time for actual discussion about the client's short- and long-term goals. In some cases, psychiatrists were perceived as being so focused on the client's short-term mental stability, that the client's overall wellness didn't seem to matter.** This could occur even when a client brought up a specific concern about a medication to the psychiatrist: His big thing was, 'It doesn't matter if you end up having other side effects from the medication. You've gotta deal with those, as long as the medication's working.' And I was starting to have blood sugar issues and hypoglycemic attacks. He didn't want to help me lower or get off my meds.	
Green et al (2008)	Somehow I think [clinician] thought I was [drinking] when I wasn't, and **I felt he didn't believe me [that I wasn't drinking]. I lost trust for him…because I was there to get help**. Obviously, I was not there because I wanted to lie to him…That was a bad experience because I know he did not trust me. On the other hand, I did not trust him because he was not believing me.	Pre-2014
(Kaar et al., 2019)	**Patients voiced concerns with regard to disempowerment in the decision-making process due to improperly presented information**. 'I was … trying to understand what the doctors are saying, because you walk into a doctors surgery at best of times, and they start talking about this medication, all this medical stuff, and you're sitting there thinking "What are they saying?..."' (P02) **The lack of information often precipitated distress and anger towards doctors and substantially affected adherence to medication.** 'They came and gave a little tub of something, a liquid, and said "Here, take this, this is going to help you!" So I'm like "Wait, how will help me?" What do you mean "it is going to help me"?! What is it?! It's weird, isn't it? Of course, I would not be ok.' (P01)	Post-2014 4*
(Aref-Adib et al., 2016)	Participants reported that they did not volunteer information about their Internet use for fear of undermining their clinician's authority. This suggests a tension between the potential independence and empowerment offered by online health information seeking and the sense of dependence and respect for authority engendered by the traditional patient-clinician relationship. **This has repercussions for shared decision-making beyond digital technology and may reflect perceived inequalities of power in the therapeutic relationship [16].**	Post-2014 5*
(Bolden et al., 2019)	At the same time, psychiatrists tend to steer the treatment discussion towards a particular outcome. **For example, psychiatrist may apply pressure on their clients by eliciting a commitment from them or direct them towards a particular treatment by marking it as a best option (**Quirk et al. 2012). […] In psychiatry, and especially in long-term psychiatric care, patients may have opinions about how well their medications are working for them, both therapeutically and in terms of side effects. However, **their opinions may not be seen as legitimate by providers since patients lack professional authority to judge medication effectiveness and they might not have insight into their illness** (Chong et al. 2013). Given the delicacy involved in advocating for particular medical treatments, it is not surprising that, across medical contexts, patients rarely request a treatment explicitly.	Post-2014 5*
(Crellin et al., 2022)	Some patients report **a lack of involvement in decisions** about their treatment and, specifically that **requests to**	Post-2014

	**reduce or discontinue medication are often not supported** [28, 29]. These factors may be related to the low adherence rates amongst patients with schizophrenia [30, 31], which are linked to increased risk of relapse […] Yet, as other research indicates, patients often report that they lack the opportunity to make collaborative decisions about taking antipsychotic medication, and support to try to reduce or discontinue this medication is rarely available [28, 29].	5*
(Ehrlich & Dannapfel, 2017)	Medication management was a particular concern for several participants who felt **excluded from decisions or discussions about medication prescription, use and possible side effects: "I don't decide anything about that [medication]"** (ID020; male, 58 years). Participants wanted to be part of their care and learn to master their problems, but believed that the GP or psychiatrist **did not always give them enough information about the medication or explain potential side effects in ways that they could understand and interpret in a personally meaningful way: "It can be quite frustrating** if obviously he's [doctor] got quite a bit more knowledge than the patient, but if he's not sharing that knowledge with you, he's [doctor] not really doing his job properly" (ID015; man, 38 years); "At times he [doctor] talks, I can't understand everything he says" (ID032; woman, 54 years). […]<br><br>However, **when health professionals did not listen to participants, the relationship was experienced as being unequal, with the professional holding the power**. Often, participants did not have the necessary skills to speak up, and rather than being active participants in their health care, became passive recipients. Thus, rather than gaining critical skills of mastering living with mental illness and managing the consequences of poor physical health, participants felt mastered by health professionals: Sometimes I think GPs get so full of themselves they forget to actually listen to the patient, and that people actually do know their bodies. Anyway, you just go along, what can you do? (ID002; woman, 35 years). **However, participants wanted to be involved in their health, share decisions and have the legitimacy to question the proposed care and not be treated paternalistically**.	Post-2014<br>5*
(Grünwald & Thompson, 2021) (Restarting the conversation)	This misalignment of goals can be problematic and prevent SDM from occurring. **If outcomes and options are not discussed and aligned this can have consequences moving forward. SUs may choose not to adhere to treatment plans, which can lead to unsafe medication practices, putting their health at risk.** […] **The lack of trust** created by previous experiences of coercion or sectioning under the Mental Health Act may also prevent SU from sharing whether they are currently experiencing symptoms.	Post-2014<br>5*
(Katz et al., 2019)	After meeting with him [the psychiatrist] once every three months, he would always ask me, "**What's your name, where do you live?" That was the thing that infuriated me. Why do you not remember the most basic facts about me?! It was so impersonal!**<br>**Ayelet felt insulted every time she visited the psychiatrist because of the lack of personal contact. This made her**	Via Citation Search

	**doubt his ability to help her.** Eventually, she decided she was ready to stop taking medication, and did so for a few weeks, but, at first, she was afraid to share this with her psychiatrist:<br>One day I was struck by the fact that I was, in fact, deceiving myself . . . I was a bit afraid of his reaction, so I told him I was taking less [medication] than what he had prescribed. I did not say I had stopped taking it completely. Then he said, "It's a sign that you don't need the medication." I was completely shocked! Suddenly, I felt it validated what I had always noticed, internally.	
(Keogh et al., 2022)	While relationships with professionals has been discussed as influential in the decision-making process, **impersonal relationships and a lack of dialogue were specifically discussed as a reason for discontinuing medications in four studies** (Bülow et al., 2016; Katz et al., 2019; Roe et al., 2009; Salomon & Hamilton, 2013). […]<br><br>For many of the participants, the process of being prescribed and taking medication was seen as an **erosion of their personal autonomy, loss of control** and for some taking medication conflicted with their personal beliefs about health and wellness (Bjornestad et al., 2017;Bülow et al., 2016; Katz et al., 2019; Le Geyt et al., 2017; Roe et al., 2009; Salomon & Hamilton, 2013; Tranulis et al., 2011). Central to this experience was the perceived lack of collaboration between the participant and the prescribing professional and the limited choices that were available to them (Büulow et al., 2016; Gale et al., 2012; Le Geyt et al., 2017; Salomon & Hamilton, 2013; Tranulis et al., 2011). […]<br><br>Furthermore, in many cases **participants reported a lack of information provision to enable them make an informed decision about whether or not to take medication** (Bülow et al., 2016; Le Geyt et al., 2017; Roe et al., 2009; Salomon & Hamilton, 2013). In Le Geyt et al. (2017) study, the participants specifically refer to a power imbalance between them and the professionals involved in their care. **This led to feelings of powerlessness and in some cases participants felt coerced into taking medication which further eroded their sense of autonomy and perceived control** over the situation. Individuals' autonomy and control was further hampered by the distress they were experiencing at the time, which also rendered them vulnerable and susceptible to coercion (Roe et al., 2009).	Via Citation Search
(King et al., 2022)	Three participants could attribute no benefits to antipsychotic use. Two felt that antipsychotics were to benefit others, as opposed to themselves:<br>"I don't feel like I'm taking [amisulpride] for my own good, it just makes me more compliant to society." – Ben […]<br><br>**Whilst on antipsychotics participants felt like they were not in control of their wellbeing.** Two participants explicitly talked about their desire wanting to "feel like me again". For some participants adverse effects were the sole reason behind their decision to withdraw:<br>"I got postural hypotension the last time I was on [quetiapine], so I'd get out of bed and collapse, and I'd wake up	Via Personal Networks

	lying on the floor." – Grace […]<br><br>**The participants felt they were not believed or taken seriously. The severity and impact of the symptoms were overlooked**. Four participants said professionals had classed their symptoms as relapses of prior diagnoses. This angered the participant especially when they had not previously experienced these symptoms. **Two participants completely disengaged with healthcare services after their withdrawal symptoms had been disregarded**:<br>"I've severed relationships with the medical system, and therefore put myself in a difficult category. I discontinued my contact, and I've improved ever since." – Paula	
(Larsen-Barr et al., 2018)	Consistent with the only other AM withdrawal study to explore the issue (Salomon et al., 2014), we found consulting a doctor may improve the likelihood that people follow a gradual withdrawal method. However, as others have found (Salomon et al., 2014), only half the sample consulted a doctor or followed a gradual taper. **Interview studies suggest some people are reluctant to disclose their plans to stop and others report feeling pressured to abandon their plans by clinicians when they do seek advice (Roe et al., 2009; Geyt et al., 2016). People may lack information and guidance** about implementing gradual withdrawal methods and appear to commonly proceed with an abrupt or swift taper that may increase their risk of relapse and reduce their chances of successfully stopping **A lack of information** about what is needed to safely manage withdrawal effects poses practical and ethical considerations for treatment systems aiming to align their practice with the principle of informed consent. It is difficult to freely choose to persist with AMs without knowledge of how to stop them.	Via Citation Search
(Geyt et al., 2017)	A challenge discussed by all participants in varying degrees was feeling heard and understood by others, including general practitioners, mental health professionals, family, and friends; this presented barriers to forming alliances. For many participants, forming alliances with professionals was **hindered by medication being prescribed and managed in a way that established a perceived power imbalance. This dynamic resulted in some participants feeling coerced into taking medication and out of control. Having the support of my doctor who didn't take a hard line with me and say "you must go back on your medication," that in itself was very helpful.** 'Cause I think if he'd said otherwise, that would have been very difficult…<br>I didn't really have any understanding about them [neuroleptic medications], I just knew that I didn't like them . . . I didn't like the effect they had on me, I didn't like the fact that I was told that I was psychotic and had to take them. I was very, uh, resistant to taking them. There wasn't much information. **Negotiating acceptability was limited for this participant by lack of information and a noncollaborative approach** by clinicians to exploring psychosis and medication. In this sample, choices about neuroleptic medication were often shaped according to whether systems facilitated or hindered decision-making processes. Le Geyt et al. 2017, p. 566	Post-2014<br>5*

(Martinez-Hernaez et al., 2020)	The patients recognised that when they are in relapse, optimal clinical communication is not easily achieved. Nevertheless, even in these cases they insisted that **the clinical response should not be one of coercive measures and high doses of medication, but dialogue to help them accept and manage their suffering, […]**<br><br>**One of the patients stated that he could hardly trust his psychiatrist if the professional did not trust him (Patients Focus Group 1). Some of them also wondered to what extent the clinicians took what they said seriously**, because the professionals might wrongly interpret their demands as distortions caused by the disease (Male Patient 38). One of the patients expressed himself in these terms, "We've developed a survival attitude. We can distinguish clearly between a professional seeing a disease and a professional seeing a person" (Male Patient 38). **[…]**<br><br>The patients highlighted a social and **clinical failure to recognise them as individuals with autonomy and agency, either in their decisions regarding medical treatments or in the broad spectrum of their lives,** since the conflicts with medication become a synecdoche for their existence and mobilise morals worlds that have profound effects on the sense of self. […]<br><br>One of the patients stated that he could hardly trust his psychiatrist if the professional did not trust him (Patients Focus Group 1). Some of them also wondered to what extent the clinicians took what they said seriously, because the professionals might wrongly interpret their demands as distortions caused by the disease (Male Patient 38). One of the patients expressed himself in these terms, "We've developed a survival attitude. We can distinguish clearly between a professional seeing a disease and a professional seeing a person" (Male Patient 38).	Post-2014<br>5*
(Pedley et al., 2018)	There was also some evidence that patients held negative views of staff, with some speaking of being **unable to trust health professionals, eroding their ability to be open and honest about their medication.43 Health professionals acknowledged that lack of patient trust could act as a barrier to involvement, which they attributed to their power to enforce mental health law.52**<br><br>Further evidence of this power imbalance was evident in the way some patients acted to appease the prescriber. Sometimes, this was due to a belief in the need to respect the medical practitioners' expertise and authority.43,48 Patients felt a pressure to 'please' the provider, 'keep the peace' and allow the meeting to progress quickly.20,32,48 **At other times, open dialogue was inhibited by a fear of the prescriber becoming angry or showing disapproval, or by patients' fear of being perceived as difficult or even manipulative.20,43,44,48 Less frequently, fears were attributed to the powers that health professionals held to bring serious negative consequences to patients, such as being removed from the general practitioner surgery list 44**. […]	Post-2014<br>5*

	Although all these factors were conceptualised as relational processes in the synthesis, it is important to acknowledge that interactions are based on relationships built over time. In addition, it should be acknowledged that interactions are likely to be influenced by broader influences, such as cultural perceptions of medicine and views of medical practitioners as powerful.58 […]<br><br>The degree to which patients could be involved in prescribing was dependent on an environment that was conducive to collaborative discussion. **Patients complained that their input in prescribing decisions was restricted by a lack of practical opportunity for discussion. Consultations were experienced as an 'in and out' process in which prescribers appeared visibly 'bothered' and rushed.**32,43,48,53 […]<br><br>One qualitative study involving people with chronic physical health conditions found that, compared with patients from high socioeconomic groups, those from lower socioeconomic groups were less likely to ask questions within consultations, believing it not to be within their role.57 This suggests that there are individual differences between patients, a subgroup of whom do not feel entitled to be involved. This underlines the need for health professionals to not take passivity at face value, but instead to help patients to establish, as Protheroe et al57 have discussed, 'permission to participate' […]<br><br>We would argue that many of the belief and attitudinal barriers identified through this synthesis reflect differences between stakeholders in the understanding of the rights and responsibilities of each individual in the prescribing partnership. Without such a shared model, it is unreasonable to expect that patients will challenge and speak up in the determination of their own treatment, and that prescribers will challenge those obstacles that inhibit patient involvement. **From the earliest points of engagement with services, patients need to understand that they have a right to involvement, which can only be removed in very specific circumstances.** Additionally, both patients and prescribers need to understand that collaboration cannot be achieved without each partner being willing to value their own, as well as the others expertise.	
(Thompson et al., 2020)	Several studies, including some of those included in this review, have **commented on the loss of autonomy** experienced by people with severe mental disorder, and their **relative lack of involvement** in decision-making compared to patients with other medical conditions [36, 46, 65]. In the current review, this was due to the ongoing or recurrent nature of the underlying condition, legal restrictions, informal pressures from family, friends or professionals, or lack of encouragement by clinicians to be involved in treatment decisions.	Via Citation Search

(Weiden, 2016)	Within the social ecology of psychiatric treatment, failure to follow the recommendation is socially undesirable. […]<br><br>Assuming that disclosure of intentional nonadherence is likely to elicit, at best, negative feedback from many clinicians, it follows that patients may find these responses stressful and difficult.25 Even if well intended, the way clinicians react to information of recent or intended medication discontinuation may be perceived as patronizing, insensitive, or as being a personal criticism. […]<br><br>**If patients' concerns and complaints about problems with medication are dismissed or trivialized, then the patients will be frustrated and feel that their opinions do not matter.** Clinicians may also react with frustration because the patient seems to be ignoring recommendations and will likely suffer serious consequences as a result. However, **it will not help the therapeutic relationship** if the clinician's frustration appears to be based on annoyance that the patient is not obeying the medication recommendation rather than true concern for the patient's well-being. Once the patient has experienced these kinds of interactions, **the patient will be more likely to take adherence matters into his or her own hands,** and decide it is not in the patient's best interests to disclose his or her true beliefs and decisions about the recommended medication regimen	Post-2014<br>5*
(Yeisen et al., 2017)	**For most participants, insufficient information evoked negative emotions such as frustration and anger, resulting in unfavorable alliances with staff which subsequently and reduced their adherence to AP**.<br>"The psychiatrist gave me rudimentary information about my medication. However, I remember that I went online and I looked for more information, I remember very well a situation when I just had increased the dose of my first AP. This led to suicidal thoughts and I proceeded to cut myself. Afterwards they informed me that this was a side effect of the medication. This hurt me a lot when I think about it. They had given me something wrong. **I thought this is not my fault; it is the medicine's fault and the doctor's fault because he had not informed me about this side effect. I remember that I felt that I had received inadequate information. I made the choice not to use any sort of AP medication"**	Post-2014<br>5*
(Kaminskiy et al., 2021)	For service users not feeling involved in discussion about options in routine, while not in crises, was associated with feelings of helplessness and lack of control. Rosie: The last time that I saw her [the psychiatrist], my medication was increased, and my mood was low but I didn't really know, **like I wanted more options and I thought that it would have been better if I had talked it through with her a bit more about increasing the dose and instead she just increased the dose and that' it. […]**<br><br>Service users referred to worries about labeling and stigma associated with a psychiatric diagnosis, and as problematic for SDM. For some participants, **diagnosis related to feeling labeled and prejudged impacting feelings of**	Via personal network

	**not being valued or listened to in conversations about medication**. Ziggy, when describing a memorable negative meeting with his psychiatrist, refers to a pretense of listening by the psychiatrist and feeling ignored:"And there are some semblances of listening, but it's not really going in because in their mind they've already put a label on me." For SUs this theme is also connected to feelings of being spoken down to, of not having a voice, and of a culture of doctor knows best. In Natasha's quote below, feelings of being attacked, looked down upon and judged connected to being unable to contribute in a conversation with her psychiatrist. Natasha: **I felt like I was being attacked and I don't know, it was like I was coming to them for help, it just felt like I was just being attacked and judged and sort of looked down on and it just made me feel really uncomfortable, upset. The fact that I was too scared to say anything, it was, you know, just horrible, it made me feel even worse**	

CMOC 4 ('Being Heard')

From the start of their relationship onwards, when an individual with SMI on medications is actively engaged by a respectful, supportive practitioner who takes an interest in them and their issues and concerns about their illness, medication and side effects (C), they are more apt to forge a therapeutic alliance with their practitioner (O), because they feel heard and listened to (M) and they trust (M) in the practitioner's motivations to help them better manage their medications and illness.

CMOC based on data extracted from 17 documents.

Document (author, date)	Extracted data	Subset of literature
(Green et al., 2008)	**The need for a good "fit" and to feel comfortable with clinicians was a common theme in participants' descriptions of what made for good relationships. Most often, participants made relatively quick assessments** upon meeting a clinician about whether or not the "fit" was good and most opted for change when it seemed poor. [...]<br>Participants described multiple factors as contributing to the fit and comfort they felt with clinicians. **Many mentioned some shared experience or characteristic, including shared values, experiences, gender, age, or religious or world views. Also of great importance were the following: the clinician attending carefully to the patient and accepting the patient unconditionally; having the right balance of closeness or distance in the relationship;** the clinician not threatening the patient (as with involuntary hospitalization); the clinician being able to have fun, a sense of humor, or a positive attitude; **the clinician providing honest feedback and pushing the patient to take on more responsibility when it was appropriate; the clinician's ability to stay calm during difficult times;** and the clinician being willing to not just "do it by the book." […]<br>Interviewer: Could you describe things you'd look for in a clinician that would be a good match for you? **Participant:** Somebody who would look at you and listen to you, and would work with me on medication, and that **I would feel**	Pre-2014

	**comfortable with, comfortable meaning at ease, and not nervous, and somebody that would remember what I had told them about my family, or about my life and wouldn't have to ask me every time I came in, somebody I felt was personally interested in me,** somebody that would return my calls within a day, and, or let me know some way why they didn't return my call. I like [PSYCHIATRIST] having [NURSE] as his nurse because I can go through her, and right away…**she's available**.[…]<br>**A caring, compassionate approach on the part of the clinician was essential in facilitating the development of the long-term partnerships we encountered**. This was the most commonly discussed and prominent theme in participants' descriptions of good and bad relationships with mental health care providers. Patients described many ways clinicians could show they cared, while conversely, the absence of these practices was often seen as indicating that a clinician did not care. **Key indicators that a clinician was caring and compassionate included the clinician listening carefully, providing hope and reassurance, returning calls during off hours, spending extra time when it was needed, and checking in to make sure all was well during difficult periods.**[…]<br>A common subtheme within the desire for a caring and compassionate approach was the importance of **clinicians listening, understanding, believing, and truly knowing their patients**. One participant said "He [psychiatrist] mostly controlled my medication. He didn't really know me as a person." Relationships with clinicians were undermined when they did not listen well, believe, or seem to understand their patients, or, perhaps worst, even remember their patients or what they had said during previous consults. […]<br><br>For the majority of decisions, the provider initiated the decision making process (See Table 1). When consumers initiated, the decision most often began with a question (e.g., "Are you still wanting to take me off meds?") or a concern (e.g., "I haven't really heard any voices. I have had sort of like, it's sort of like someone's … talking to me, but I'm not getting clear words.") Outright disagreement was rare.	
(Matthias et al., 2012)	For the majority of decisions, the provider initiated the decision making process (See Table 1). When consumers initiated, the decision most often began with a question (e.g., "Are you still wanting to take me off meds?") or a concern (e.g., "I haven't really heard any voices. I have had sort of like, it's sort of like someone's … talking to me, but I'm not getting clear words.") Outright disagreement was rare.	Pre-2014
(Clifford et al., 2020)	The prescriber's **interest in the consumer's experience** (reflected through in-depth questioning), their knowledge of relevant background information and the degree to which prescribers tailored their medication regimens to their individual circumstances, including symptom fluctuations, stressful situations and lifestyle factors, were important in successfully encouraging adherence. Having a "good relationship" with the treating psychiatrist and **being able "to feel heard" encouraged interviewees to adhere to their medication**. […]	Post-2014<br>4*

	Interviewees indicated that they **wanted their prescribers to ask them relevant questions** to assist with their illness and treatment management, which was contrasted with experiences of an impersonal service from prescribers, who forgot simple details including those related to the medication regimen…	
(Bjornestad et al., 2020)	What worked was when I told my therapist how I was doing, and he managed to tell me in another way why I felt that way…. I think my Community Psychiatric Nurse takes on board what I say she's quite good, **I can like test the waters with her and then we will think about it and not just on one single answer but look for a variety of avenues to follow** (Bjornestad, Davidson, et al., 2017)	Post-2014<br>5*
(Ehrlich & Dannapfel, 2017)	Gudzune, Beach, Roter, and Cooper (2014) found that **patients who trust** their primary care provider are more likely to be committed to the primary care relationship and more likely to adhere to medical advice; it may determine whether or not they take their medication as prescribed, reduce their dosage, or stop taking it completely. […]<br>**For your doctor to remember your first name, or see your face and know it's you.** When I do get emotional and then I start questioning myself, he reassures me and says that it is nothing to do with that [mental illness], it's called being human…" (ID002; woman, 35 years). When participants were able to develop relationships with doctors who had these qualities, they believed that **they could talk openly with the doctor about their problems.** Participants highlighted that they **wanted their concerns to be taken seriously,** and that GPs would act to uncover whether or not there was a physical basis for their concerns.[…]<br>**Being like everyone else was an important issue, as well as being treated respectfully by people involved in their health care, who should be available and accessible when necessary.** […]<br><br>All participants stated that their relationship with their GP was important. **Qualities such as being respectful, understanding, supportive and making participants feel comfortable by taking their issues seriously were valued highly.** […]<br><br>**Listening to participants was critical to ensure that they felt as though they were credible partners in producing their health outcomes**: He's [doctor] very understanding. **He listens. He takes everything very seriously; he doesn't just dismiss or, you know, thinks it's her [participant's] mental illness or whatever**. He always dig[s] deep to find what's wrong…he's only quite young but he's really good to me (ID 001; woman, 47 years). […]<br><br>Our findings highlighted the importance of the relationship with health professionals; the way that physicians **engaged with participants affected their willingness to share their story**, which in turn affected the type of care that was provided and the degree to which shared decisions were achievable. Respondents were more likely to talk about their problems with a **physician who listened, and showed interest and respect.** The ability of health care professionals to	Post-2014<br>5*

	communicate openly and honestly had a positive impact on relationships and was an important channel to improving quality of care. […]<br><br>**Thus, partnership, mutual dialogue and listening were aspects that increased the participants willingness to share personal information, which enables health professionals to suggest appropriate care. […]**<br><br>**Everyone's very cool to me. They treat me like a person without a mental disorder, but at the same time they're always there just in case I need it… they just treat me like a normal human being that had a problem, and I like that**" (ID016; man, 28 years).	
(Fiore et al., 2021)	Some patients reported that the drug was initiated during an exacerbation of the disorder, when they had no capacity to choose, and that **trust in the doctor** and the therapeutic alliance were very important during recovery in the decision to continue to take medication or not[16]	Post-2014 5*
(Haddad et al., 2014)	**The clinician needs to listen to the patient, understand their perspective, including their beliefs and concerns about their illness and medication, a**nd ensure that their preferences regarding treatment are based on fact rather than misperceptions. Involving patients in the choice of their medication increases the likelihood of adherence.84,85	Post-2014 5*
(Haugom et al., 2020)	**SDM involves presenting information about the patient's health issue and treatment options, acknowledging their values and preferences, discussing pros and cons, and considering the patients' abilities and self-efficacy**. Further, when the health professional presents their recommendation, the SDM practice is to check and clarify the patient's understanding, make or explicitly defer a decision, and arrange follow-up [2]. **[…]**<br><br>**…the time factor is very important, you spend time building an alliance and trust**. And the quality of SDM, or **the quality of the choices made, will increase when you trust the person who's suggesting something.** So that's why I support this idea that the time factor is absolutely crucial. (P2, outpatient care) **Several participants conveyed that patience is needed to help patients to open up and talk about symptoms that they had been too distrustful to mention previously.** According to several participants, as the **patient's suspicions decrease and their confidence in the relationship increases**, a firmer basis for co-operation on treatment options develops. **[…]**<br><br>Some participants said that patients with psychotic disorders must be fully respected. Health professionals must be willing to let these patients make a choice and live with the consequences of that choice, even if they disagree.	Post-2014 5*
(Jakovljevic, 2014)	**The individual patient preference is very important. Linking LAAIs to the patients' individual goals and desires may significantly contribute to the achieving therapeutic alliance and**	Post-2014 5*

	**better treatment outcome**.	
(Geyt et al., 2017)	The **first task** involved participants **striving to understand the need for, and acceptability of, taking neuroleptic medication within the context of their lives.** This involved developing a theory of these issues over time and acquiring knowledge to support decision making. One participant described the value of this: "I've always maintained that if you've got a mental illness . . . getting more knowledgeable about it somehow empowers your brain to sort itself out a bit more." […]<br>The desire to understand whether medication was needed and if so, in what dose, was of foremost concern to participants. **Most participants aspired to develop a shared understanding with others, particularly their treating clinicians whose opinions were considered influential.**	Post-2014<br>5*
(Martinez-Hernaez et al., 2020)	Trato or buen trato means both having a pleasant manner and dealing with or agreeing with someone and presupposes an **intersubjective relationship as well as negotiations** with another person. […] In short, we can say that patients **expected to receive a kind trato that was sensitive to the problems beyond their medical condition and that allowed them some room for negotiation, including the recognition of their rights.** Here trato comes close to the notions of concordance and supported decision-making. […] In their view, **good trato can be defined as proximity, empathy, dialogue, negotiation, feeling that their vital needs are heard and receiving unhurried attentive information** about their disorder and the effects of medication, especially when they are under polypharmaceutical treatment.	Post-2014<br>5*
(Pietrini et al., 2019)	Conversely, from a clinical perspective, focusing on patient's **subjective experience offers precious insight into each individual's personal recovery and into his or her strengths, weaknesses, wishes, activities, and preferences**. The personal meaning that each person attributes to his/her illness and to the treatment received within his/her life context is central, to address an optimal recovery.20 […]<br><br>**Understanding the attitudes of patients and caregivers** can thus represent a valuable asset in tailoring treatment to expressed needs, and has the potential to improve the therapeutic alliance and medication adherence, thereby enhancing long-term prognosis.22 […]<br><br>The first contact with mental health professionals can understandably be perceived as a source of stress by patients and caregivers, especially in cases of early hospitalization due to an acute episode. In consideration of all of these aspects, it is necessary to actively engage patients, starting with the very first contact with mental health services. **This implies investing time in listening and understanding** the patient's feelings and values, in order to create a virtuous circle aimed at **achieving practical and individualized goals that can in turn boost treatment adherence.31**–33	Post-2014<br>5*
(Salzmann-Erikson & Sjodin, 2018)	Participants stated, among other things, that it was pleasant to visit the outpatient facility. In particular, the contact nurse was described as being a stable contact in terms of being a consistent, attendant and reliable person and thus provided a "sense of order". They [SUs] also reported that the contact nurse was able to spend quality time with the	Post-2014<br>5*

	patients during administration of the injection, which was highly valued. During these encounters, the participants were **able to talk about any matters that concerned them** (Phillips and Mccann, 2007).	
(Velligan et al., 2017)	In another prospective observational study, better **patient-reported therapeutic alliance at baseline was associated with better adherence of patients** with early-episode schizophrenia 6 months after hospital discharge.[22]	Post-2014 5*
(Watts et al., 2021)	**Supportive professionals** including medical consultants, nurses, GPs and psychotherapists were identified as being important, when available. In addition to providing advice on medication management, offering tips related to sleep and other behaviours, **professionals who listened, demonstrating empathy**, and responding to participants' wishes to discontinue medication in an affirmative, collaborative and holistic manner were valued. [names doctor] made out a plan, he gave me a time I'd be off them. .. .he gave me advice on sleep, put me on to a counsellor.. . the most important person for me is my counsellor [P 21; F] There was a nurse that I got on particularly well with...she has been a lifesaver to me. Because in the early stages of my recovery just being able to talk to her...there was a huge amount of affirmation involved in that process. She's been a huge positive influence on me, believed in me. […]<br><br>**In the few occasions where practitioners moved away from a zero-tolerance approach and engage in dialogue around participant desires and wishes to discontinue medication, participants not only valued this approach but the felt listened to and valued as a person**. The seriousness of some of the withdrawal reactions experienced by participants in the study, including suicidal thoughts, relapse and rehospitalization, and the growing body of literature on discontinuation syndrome (Cosci & Chouinard 2020; Tondo & Baldessarini 2020), highlights the importance of clinicians adopting a harm-minimization approach.	Via Citation Search
(Weiden, 2016)	For patients who are ambivalent or opposed to antipsychotic medication, our belief is that it is best to **take a longer term, developmental perspective. The road to better adherence may take a while, and in the long-run it is more effective to focus on the therapeutic relationship**. This can be accomplished through dropping adversarial or imperious responses to patients' concerns, and **focus on understanding those concerns before responding.** Finally, when nonadherence is a choice and seems inevitable, then it is best to stop trying to "stop" nonadherence, and rather **to focus on coming up with a collaborative harm-reduction strategy** to try to mitigate the risks and consequences of medication discontinuation.	Post-2014 5*
(Kaminskiy et al., 2021)	The vast majority of service users and CPN participants mentioned the importance of service user's **experiential knowledge for meaningful sharing of expertise in decisions. The importance of both parties having a say, and equally contributing to the conversation**, is seen as integral to SDM. […]<br><br>Psychiatrists also emphasized the **importance of honesty and, at times, disagreement was considered a success (and highlighted this during descriptions of successful meetings). However, CPNs and service users directly expressed the importance of experiential knowledge and a shared dialogue**. […]	Via Personal Network

	Service user participants were aware of the effort required by both parties to establish a deeper relationship and understood that SDM requires honesty on both sides. **Several participants referred to not just establishing a general rapport but rather an ongoing effort in building mutual trust**. […]<br><br>David: I have to do everything I can to allow myself to get better but if it means I have to trust somebody that I don't know, which is very, very difficult for me to do, then so be it. Carrie: **I think the main thing is to be as honest as possible.....the honesty and the trust I think as well, and you know you kind of build up a relationship with somebody and you get to trust them.** When describing memorable positive meetings, psychiatrists referred to **establishing rapport and service users feeling able to speak openly and honestly.** Some psychiatrists acknowledge that there are potentially differing agendas in conversations concerning medication [and that conflict may emerge when medication is deemed as the best course of action [see (28)]. In this context, service users feeling able to express their views honestly is seen as a particular success.	

CMOC 5 ('Practitioner information exchange')

From the start of the therapeutic relationship onwards, when an individual with SMI feels comfortable accessing their practitioner for honest, easy-to-understand and personalized information about their medications (C), they are more apt to use the information to prepare for and to cope better with medications and side effects (O), due to development of mutual trust (M) and mutual respect (M) in each other and in the information being exchanged.

CMOC based on data extracted from 19 documents.

Document (author, date)	Extracted data	Subset of literature
(Green et al., 2008)	The following passage illustrates another aspect of comfort in clinical relationships– interpersonal distance. It suggests that the clinician was able to provide care with the **interpersonal distance** this participant preferred. […] When patients were not comfortable with their clinicians, some participants mentioned that it could lead to poor treatment adherence. […] **Conversely, comfortable relationships encouraged communication about decisions and concerns that could affect mental health outcomes in important ways:** I am thinking of trying some Dexatrim, or something like that because I take [medications] which increase my appetite…! want them [clinicians] all to know about it because my behavior can change with the addition of an appetite suppressant, or whatever. I'm not going to do it without a doctor's supervision. I'm going to consider it. I'm going to try it, but **I feel comfortable enough to ask them about it instead of going behind their backs. That's an important thing to me.** […]	Pre-2014

	**Many of these discussions addressed how important it was for participants to have knowledge about medications, support from clinicians in controlling and making day-to-day medication adjustments, and clinicians responsive to requests for medication changes when symptoms worsened or side-effects were problematic**.	
(Opler et al., 2004)	We had used our knowledge of cultural differences to become a Hispanic mental health clinic, but not until we understood our population more thoroughly did this become more than just a change in name. **Our patients had taught us the importance of considering cultural factors**, even in something so seemingly straightforward as prescribing medication, and of being sensitive to the complex ways in which our own culturally based assumptions could undermine our attempts to provide effective treatment. […] **The lesson to be learned here is that, in standard clinical practice, psychiatrists do not routinely explain the full spectrum of possible side effects to patients. Rather, they tend to wait until such side effects are mentioned by patients before addressing them. Given that some side effects may have particular significance for members of certain cultural groups, it is recommended that clinicians have an in-depth discussion of possible side effects with patients and assure them that there are treatment strategies (**e.g., amantadine for neuroendocrine side effects) that can alleviate some of these side effects. **Such discussions can improve patients' confidence in the practitioner and increase medication compliance.**	Pre-2014
(Rungruangsiripan et al., 2011)	**Therapeutic- alliance had a positive direct effect on illness representation and an indirect effect on intention to change adherence behavior**. This suggests that a **therapeutic alliance may help patients observe their bodies, thoughts, and other symptoms, which help them create an individualized picture about their illness. […]**<br>Efforts to cope with their illness depend on the components of the illness representation and what interventions have been proven helpful. Normally, the illness representation is vague until experience and outcomes begin to frame the important elements for each participant. **When patients interact with health care providers, they exchange information about illness discomfort and medication side effects, symptom experiences, and crisis situations (Diefenbach & Leventhal, 1996). The information exchange from the interaction between patients and health care providers can initiate a comparison between the initial perception and the reference value from health care providers. Thus, patients reanalyze and develop important self-care strategies, such as medication adherence (Leventhal et al., 2003).** […]<br><br>As Leventhal et al. (1998) postulated, **coping procedures relate to illness representation. Coping derives from illness representation by "if–then" rules** (Leventhal et al., 1998). […]<br>The finding of a causal relationship between illness representation and intention to change adherence behavior and adherence behavior reflected an appraisal process of illness representation, which generated adherence intention and medication adherence. **Patients appraised the idea that adherence intention and medication adherence could help them manage the illness threat,** […]	Pre-2014

	**…** it emphasizes that patients have the ability to use their cognition to think about illness and generate their own coping behaviors to manage the illness threat.	
(Seale et al., 2006)	Seven of the doctors made comments about the value **of honesty in** relationships with patients. **This involved being as honest as possible about risks, particularly with regard to adverse effects, developing a reputation for being 'straight' with patients and for facing up to difficult issues such as the limitations of one's own knowledge.** Although one mentioned that this was an ethical way to behave anyway (interview 19), all mentioned that there were potential benefits to this approach in building up trust. I don't feel that I have all the answers. I feel that's very liberating (laughs). If I don't know something, I just tell the patient, 'I'm sorry I don't know that' or I feel happy to say, 'I'm not very good at that.' (Interview 13) […]<br><br>**When asked how they found out about what patients were doing with their medication, the most commonly mentioned approach was to ask patients (19 doctors), as in 'You rely on self report' (Interview 11) or 'In the vast majority of cases I have to rely on their account' (Interview** 15). Several distinct approaches were described to eliciting information from patients. The most common (11 doctors) was to indicate that non-compliance was normal, and that reporting it would not be followed by disapproval. Six mentioned the **value of having a trusting and co-operative relationship in which honesty feels right, and** one described appealing to the wisdom of knowing true intake if future dosage was to be planned rationally.	Pre-2014
(Delman et al., 2015)	Clients who expressed a higher level of activation (i.e., not just expressing preferences but negotiating towards an agreed course of medication) reported that their psychiatrists had **actively encouraged them to participate and express opinions, often early on in the relationship: She said that she wanted to hear what I wanted to when we worked together. She would give me information about a medication, and it would be up to me if I should take it.** And if I didn't want to take it, we'd talk about it, and see if we could make that medication work by a change in dose or whether another treatment made sense. Many of these psychiatrists even asked the client to describe what they saw as their problems, not focusing so much on a diagnostic category initially. **This was essentially an "ice-breaker" that set the tone for the ongoing relationship.** […]<br><br>Respondents who were at a negotiating level of activation recognized that that their **psychiatrists' willingness to make him/herself available** as needed for consultation or treatment was a critical component. These psychiatrists reportedly informed their clients of a specific way they could be reached (usually a phone number) if the client was having any serious problem. When a person called, the psychiatrist would get back in touch with him/her within 24 business hours and decide with the client for him/her to increase/decrease a dosage, to come in for an immediate appointment and/or plan to increase the frequency and/or length of future meetings. In one instance, a psychiatrist went out of his	Post-2014 4*

	way to be both accessible and to assist his client with her housing situation: And he's like, 'You know, call me, even with medication changes.' And he gave me his pager number. He gave me his cell phone number…	
(Morant et al., 2018)	There were some accounts of positive interactions and relationships with practitioners regarding medication management, expressed by a minority of the willing and resigned accepters groups. **These people felt the psychiatrist took their concerns about adverse effects and physical health more seriously and that this was reflected in how decisions were made.** […] Strategies for enhancing shared decision-making in relation to antipsychotic medication may need to include practitioner training, patient empowerment and attention to the requirements of a genuinely therapeutic relationship in order to overcome powerful interactional inequalities and the impacts, for some, of histories of difficult, mistrustful or coercive relationships with services (Morant et al., 2016). Enabling broader discussions that explore service users experiences of antipsychotics, and their long-term preferences and goals, key components of genuine shared decision-making; (Charles et al., 1997), are consistent with broader cultural shifts towards mental health services that aim to promote recovery, patient-centred care and self-management.	Post-2014 4*
(Phan, 2016)	**A good therapeutic alliance, which can be created through adequate planning, and consistent, accessible contact with patients, can also lead to improved medication adherence**.1 Patients who were able to develop a beneficial alliance with their provider within the first six months of treatment were more likely to remain engaged in treatment, both psychotherapy and pharmacotherapy, and had improved outcomes after two years.4 […] To foster these relationships, patients and their support systems should be comfortable asking questions and discussing concerns in **a non-judging, non-blaming environment**.4 The provider can also ask open-ended questions to encourage dialogue; doing so can also help the provider to identify and clarify misconceptions about the illness or treatment and ultimately **build trust and mutual respect.4 Again, accessibility** of patients to services via elements such as minimal waiting times to see providers or **timely responses** to questions or requests for when issues or concerns arise can also enhance the therapeutic relationship.1	Post-2014 4*
(Aref-Adib et al., 2016)	Two participants in the parallel universe group[not collaborative] reported feeling anxious and hopeless after reading or watching YouTube videos online about mental illness. Both described how this material left them feeling confused and unable to process the information. This was in contrast to the experiences of the collaborator group, where some individuals reported **consulting a doctor in order to check the accuracy of the information they had found, which relieved the anxiety created by this supplementary information. "There's a lot of information on the Internet that may not necessarily be correct. It may be correct at the time but it may have changed. So I was … checking and then going back to the doctors and asking."** (P13, collaborator) […]<br><br>A partnership approach to online health information-seeking is needed with mental health clinicians encouraging patients, particularly those with a longer psychiatric history and from an older age group, to **discuss information they**	Post-2014 5*

	**have found online as part of a shared decision-making process**. […] …patients want mental health clinicians to recommend websites and appropriate resources. This could provide an opportunity to initiate dialogue around patients' mental health related Internet use. In addition, professionals could play an important role in enabling patients to critically evaluate and interpret information that they read on…	
(Bolden et al., 2019)	In treatments for chronic conditions, participation in decision making is more fruitfully viewed as a process which may **unfold over the course of an encounter, or even multiple encounters** (Say et al. 2006), and, in the case of psychiatric disorders, is inextricably tied to the context of **an on-going therapeutic relationship (Matthias et al. 2013).** […]<br><br>In line with the prior research, our analysis shows that, in psychiatry, clients commonly convey their preference for a medication adjustment implicitly rather than request a change directly.	Post-2014<br>5*
(Bjornestad et al., 2020)	Obtaining proper information, either from the treatment provider or from personal reading, and thus becoming knowledgeable about one's own condition and process, seemed important when moving from the short-term horizon to thinking about living with the challenges over a longer-term perspective. […]<br><br>**Further, information appears to best facilitate successful use when delivered in a manner that supports and sustains the person's concerns with his or her autonomy and individual efforts. A straightforward and honest use of everyday language** can promote a collaborative framework (Dixon, Holoshitz, & Nossel, 2016; Thomas, 2015), and **a respectful tone** was considered a powerful remedy for early discontinuation. Patients described preferring communications to be especially **clear and to include repetition** of important aspects over time. […]<br><br>Other information sources, such as the Internet, social media, and peers, augmented the dialogue between patients and professionals. Gaining knowledge, comparing drug effects, and learning from others with first-hand experience of antipsychotic treatment were commonly used strategies in moving from an initial surrender to authority to forming an autonomous opinion on the process, with an **increasing sense of personal agency** as a result.	Post-2014<br>5*
(Grunwald et al., 2021)<br><br>(Role of hope and Trust)	Increased trust has been associated with a better therapeutic alliance [43, 54, 60, 61, 82]. Given that there are multiple types of antipsychotics and dosing options, varying responses to antipsychotic medication, and no guidelines on how to review and reduce medication [16], GPs and SU encounter many uncertainties. **Managing these uncertainties together requires a trusting relationship between GP and SU** [60, 61]. Any history of coercion or sectioning under the Mental Health Act can make developing and maintaining trust more difficult, but a **trusting relationship is key to shared decision making** [29, 82]. Given the power imbalance between SU and GPs, and often held view that "doctor knows best" [82] **the onus might be on the GP to start the conversation** […]	Post-2014<br>5*

	A recent systematic review also identified expectations of low capabilities, lack of trust and paternalism (including the decision to limit the amount of information regarding adverse effects shared and "doctor knows best" mentality [87];) as barriers to patient involved prescribing. […]<br><br>Conversations about medication should include sufficient information about antipsychotic medication, and side effects as well as benefits. **Increasing SU awareness of potentially severe side effects has been associated with increased trust** between SU and GP [60, 82] and allows SU to prepare for side effects and return to the GP for help if they persist or cause problems. […]	
(Grünwald & Thompson, 2021) (Restarting the conversation)	Outcomes and options should be openly discussed and weighted […]<br>(1)Exchange of personal and medical information should flow in both directions (2) Decisions involve both the patient and the clinician (3) Outcomes and options should be openly discussed and weighted (4) Decisions are joint efforts requiring collaboration and balanced description […]<br><br>**Exchange of personal and medical information should flow in both directions. In any appointment it is crucial that both SU and clinician have all the relevant information available to be able to make informed decisions.**	**Post- 2014 5***
(Haugom et al., 2020)	Several participants shared that a t**herapeutic alliance needs to be built to access information about what the patient wants, and to help them:**<br>That's what I think is most important. To build an alliance between you and your patients. (P3, in- and outpatient care).	Post-2014 5*
(King et al., 2022)	As with previous research, the findings showed a particular value of being connected with other people with shared experiences via online user-led groups (Geyt et al., 2016). A common reason for accessing peer support is following failed doctor or psychiatrist supported tapers (White et al., 2021). […]<br><br>More needs to be done to move away from the historically paternalistic negotiation styles in psychiatry (Slade, 2017). The findings highlight the need to **inform service users from the outset** of what can be expected of withdrawal so that when they start to consider it, they are aware of what facilitates positive withdrawal outcomes. **This will require fully informing service users, prior to the initial prescription,** of the risks associated with antipsychotic usage and discontinuation. This should also include raising awareness of minimal medication approach and medication-free	Via Personal Network

	treatment, currently officially offered only in Norway (Cooper et al., 2021; Oedegaard et al., 2020). Facilitating more **holistic consultations and medication reviews** exploring service users' priorities and goals, are consistent with broader cultural shifts towards promoting person-centred care.	
(Mahone et al., 2016)	Because nurses often have extensive and recurrent contact with clients, nurses can play a critical role in explaining to them the importance of medication follow-through and may be the first to detect clues to nonadherence, such as a missed follow-up appointment or missed appointment for administering an LAI (Kirk Morton & Zubek, 2013). When discussing treatment options, it is beneficial to understand clients' treatment goals, to help them select a medication that meets their personal preferences, and to clearly explain potential treatment related adverse event	Post 2014 4*
(Pedley et al., 2018)	Patients felt that being given information of a higher quality and quantity could lead to increased involvement, not only through increasing their knowledge, but also by enhancing their confidence and levels of empowerment.[20,32,34,37,39,44,48] The importance of patients' confidence was highlighted in one study of young adults with SMI, which indicated that involvement increased through gaining knowledge and by having a prescriber who encouraged involvement.[32] Confidence was also increased through successful prior experiences of involvement, achieving accomplishments, gaining control over life in general or simply through growing older.[32.]	Post-2015 5*
(Weiden, 2016)	Another aspect of any therapeutic relationship is trust.[26] **Trust arises over time, and is based on expectations of honesty, integrity, and predictability.**[27] Trust as it pertains to adherence issues is that the treatment recommendation will be congruent with expected outcomes.[28] Although it is essential for the clinician to clearly articulate the benefits of antipsychotic medication for successful treatment, and the importance of staying on continuous antipsychotic therapy for long-term treatment, it is also important **to be honest** about limitations of current therapies.	Post-2015 5*
(Yeisen et al., 2017)	"It was a nightmare, like you were imprisoned in the hospital, you can't get out. It was quite a disgusting experience... 'No, you can't go outside today anyhow, there is not enough staffto do this. They could have talked to me about it as an equal human being in a way. Rather than saying 'No'. They did not have a conversation with me. They let me just sit there in my room. How could they get to know me when they didn't talk to me? I felt neglected. It was very frustrating. So I decided to discontinue my AP immediately after discharge" […]<br><br>**…some of the participants felt that they got thorough information from professionals about AP effects and the most common side effects. These patients stated that they remembered the conversation with their psychiatrist about their AP at the acute stage.** This type of information was linked to favorable adherence, reduced stress levels and helped patients cope with medication side effects. "I understood that they wanted me to try the medication. However, I think it is very important, that I should know about the side effects prior to use. I also had a medication counselling	Post-2015 5*

	session where it was presented that the side effects were not strong at all, that these pills would have no serious consequences, and that it was fine to use them. So I decided to use them, and I've used them ever since." […]<br><br>Some patients got information about expected duration. This was perceived as having a positive influence on adherence. "I asked the psychiatrist what he assumed would be the duration of my using APs. Also I had a dialogue with my General Practitioner after discharge, and she stipulated minimum 6 months to one year…" […]<br><br>Adherence to antipsychotic medication is a complicated phenomenon commensurate with complexity of human unique interaction and subjective interpretation of the world. Many elements have a prominent impact on it, and these factors may vary depending on patients' experience during their illness journey. […]<br><br>**The patient-therapist relationship seems to be a crucial factor, and the initial contact with the patient is the key to the creation of an empowering alliance. This must be done with care and respect. Additionally, information about medication should be repeated once the person is out of the acutely psychotic stage of illness,** as an interactive process throughout the course of treatment to strengthen the shared decision-making element of the treatment process. […]	
(Kaminskiy et al., 2021)	Receiving a full explanation of options and gaining detailed information about adverse effects was related to feelings of increased control for service users and referred to specifically by clinicians when describing memorable examples of success. **For service users, being able to understand the information about the options were associated with feelings of increased control**. However, this was tapered by acknowledging that during periods of crisis increased guidance and less ownership over the decision is possible and the "sad truth you just need someone to treat you" (Holly∗) (see section Being Ill as a barrier). In these more difficult times, having information to take away and revisit was associated with feeling more in control.<br>Casey: But I just think if I'd been given that information and going through it yourself and having time to discuss it, you're going to understand. I just think you'd feel like you had more control and, you know, that might reduce stigma, as well as you feeling you can take control of what's going on. […]<br><br>**All stakeholder groups stressed the importance of weighing up information and ensuring service users are provided with information about the potential adverse effects of medication options**, advocated in standard models of SDM (9).	Via Personal Networks

CMOC 6 ('Info seeking behaviour non-clinician')

Whenever an individual with SMI on medications desires additional information about their illness, medications, and potential side effects (C), they will often seek out accessible, easy-to-understand information from a variety of non-practitioner sources (e.g., peers, Internet) they perceive to be trustworthy and credible (O), due to need for increased knowledge (M), increased reassurance (M) and greater control (M) with respect to medication and life decisions.

CMOC based on data extracted from 10 documents.

Document (author, date)	Extracted data	Subset of literature
(Rungruangsiripan et al., 2011)	**Leventhal's Common-Sense Model of Illness Representation** could be used as a theoretical explanation for causal relationships affecting coping and adherence behavior in chronically ill patients. […]<br>**According to the model, it is believed that people are active problem solvers who perceive and interpret information from various sources, then generate an illness perception relative to symptoms they are experiencing**. Patients generally receive **information from three sources**: the generalized pool of illness information current in the culture, the social communication or information obtained in direct contact with other people (particularly practitioners), and the individual's personal illness experience (Hagger & Orbell, 2003; Leventhal, Leventhal, & Schaefer, 1992; Leventhal, Nerenz, & Steele, 1984). […]<br>**Components of an illness representation are identity, consequences, timeline, controllability, and cause of illness**.[…] Illness representation is used to generate a coping response. The coping response consists of action intentions and actions that are expected to solve problems arising from illness (Theunissen, deRidder, Bensing, & Rutten, 2003). According to the coping response, patients will evaluate the effectiveness of the behavior in the appraisal process. If the patients appraise the coping responses as effective, they will retain the illness representation and coping response. If they appraise a coping response as ineffective, then their illness representation and coping response will likely change in accordance with the appraisal outcome.	Pre-2014
(Alguera-Lara et al., 2017)	To help patients to prepare for the consultation it is important they have access to appropriate information. **Being informed and having the opportunity to discuss the information with others prior to meeting with the doctor may enhance self-confidence** and help address the barrier of insufficient time in the consultation. […] A controlled trial of patients with acute schizophrenia showed that the use of a decision aid booklet in conjunction with nursing staff support increased patients' perceived involvement in decisions, **increased their knowledge** about their disease and improved attitudes towards treatment.14	Post-2014<br>4*
(Delman et al., 2015)	Most respondents reported using the Internet to review information on medications already prescribed by the psychiatrist. They did so in order **to clarify their expectations for a medication's** potential side effects and risks or to check out a current health concern as a side effect. Most of these respondents didn't reference the Internet	Post-2014<br>4*

	information sources during their psychiatric meetings. They didn't think their psychiatrist would react negatively to Web references, but they also did not see the value of making a Web reference. A large majority of respondents used the Internet to learn more about their mental illness or their health generally. Many of them relied on the website WebMD for their information needs and **considered it a reliable source**. WebMD is a comprehensive and the most popular source of medical and health information, featuring specific search options, a section on prescription drugs, and the latest headlines from medical and health communities.2	
(Kaar et al., 2019)	Part of the debate revolved around what was perceived as actually helpful. Some employed all available resources **to find out more about their** illness, medication, and what to expect from mental health services .' … wanting to know more about your illness and your medication if you've got the option. I do go to the library a lot and read a lot of books and the causes of schizophrenia. It's quite interesting to read because … when you read some … it's like you are reading about yourself.' (P10) […]<br>Our data suggests that being actively involved in decision making about antipsychotic treatment is important to patients, and the discussion should consider adverse effects, mode of administration and symptom relief. Clinical encounters that **increase patient knowledge and maximise autonomy** in order to prevent early negative experiences with antipsychotic medication are likely to be beneficial	Post-2014<br>4*
(Aref-Adib et al., 2016)	It is reported that more than half of the people with first episode psychosis use the Internet as a source of information about their mental health [4] and that over 50 % of the people with psychiatric problems **use the Internet** to find out about their diagnosed mental health condition [5]. […]<br><br>Information about medication and medication side-effects was the most common topic of mental health enquiry online (n = 15). Participants described going online to enhance their knowledge and understanding because the information they found was more detailed and indepth than other resources, such as leaflets or clinician provided information. Several participants described **how accessing this detailed information helped them to feel better informed with regard to their medication and mental health problems, and how this led to a sense of security and reassurance**. […]<br><br>Some participants **described looking for advice from people with lived experience on how to cope with their diagnosis or manage their mental health, while others simply wanted reassurance** that they were not alone in their experiences of mental health problems. One participant reported that reading about other people's experiences had helped her make sense of her own and another described how reading recovery stories gave him hope for his own future (P08).<br>"Reading other people's success stories regarding how they've gone back to a normal life can be, you know, somewhat **reassuring."** (P08) […]	Post-2014<br>5*

	Many participants who used the **Internet** to search for mental health information described the benefits of having access to current and in-depth information online that was more accessible across space and time than other sources, including clinicians.<br>“It’s readily available, it’s easily accessible. Not that a clinician isn’t, I mean … you can access it any time that you want … I suppose the accessibility is … really positive.” (P04)). […]<br><br>For those in the collaborator group, both the act of independent research online and the understanding and knowledge gained as a result were closely linked with feelings of control and empowerment. “**I think for one thing, it makes me feel more in control of things to be able to look at things independently and to get new information about it.”** (P04, collaborator) […]<br><br>Four participants spoke about the usefulness of having links or mental health information on the **apps, which would allow them access to reliable and credible information through their mobile devices**. […]<br><br>Our research has shown that people affected by psychosis **appreciate the accessibility of online health information and find this empowering.** This is supported by existing evidence [7]. However, the results of this study suggest that while some participants find online information helpful and reassuring, for others who do not use this information collaboratively with their mental health team, it can lead to concern and affect health related decisions, including medication adherence. Since medication non-adherence is associated with a number of negative outcomes for people with psychosis [15], this is an important finding and warrants further research.	
(Bjornestad et al., 2020)	Achieving autonomy seemed to emerge from increased knowledge via information from professionals, peers and, importantly, one’s own explorations and experimentations. **Becoming knowledgeable helped the person to develop autonomy** in the face of his or her initial sense of surrender, and subsequently to establish a sense of personhood. (Bjornestad et al., 2020, p. 519.) […]<br><br>Other information sources, such as the Internet, social media, and peers, augmented the dialogue between patients and professionals. **Gaining knowledge, comparing drug effects, and learning from others with first-hand experience of antipsychotic treatment were commonly used strategies in moving from an initial surrender to authority to forming an autonomous opinion on the process, with an increasing sense of personal agency as a result**. Patients regularly used this information to challenge expert decisions and negotiate treatment choices. Bjornestad et al., 2020, p. 519 […]	Post-2014<br>5*

(Keogh et al., 2022)	For example, participants in Le Geyt et al. (2017) study spoke of searching the internet and books, and **developing confidence** over time to seek information from the psychiatrist and more informed others. Based on information received on their 'illness' and the advantages and disadvantages of taking medication, participants began to question the received wisdom of the medical model and biochemical theories, which in turn led them to formulate a personal perspectives or 'hypothesis' on the need to continue taking medication. This included seeking out peer support from other people who had similar experiences as in Gale et al. (2012) study.	Via Citation Search
(Geyt et al., 2017)	The first task involved participants striving to understand the need for, and acceptability of, taking neuroleptic medication within the context of their lives. **This involved developing a theory of these issues over time and acquiring knowledge to support decision making. One** participant described the value of this: "I've always maintained that if you've got a mental illness . . . getting more knowledgeable about it somehow empowers your brain to sort itself out a bit more." Le Geyt et al., 2017, p. 563)<br>Some of the elements described by participants to serve as ideal safety nets included successfully formed alliances (family, friends, professionals), peer support (self-help groups, community groups), practical resources (having written indicators of relapse to aid self/othermonitoring of mental health), access to alternative treatment (talking therapy), skills in relaxation, healthy lifestyles, and access to resources online or in libraries. **Knowledge was deemed an essential component: The knowledge of all the various other things, it's like the safety net is getting stronger because you've got so many other things that are in place, that if something does go wrong because you've come off the medication, then other things'll compensate . . . other things in your life are there**. Le Geyt et al., 2017, p. 567	Post-2014 5*
(King et al., 2022)	Describing withdrawal effects to healthcare providers was challenging and it was common to that only peers appreciated how challenging symptoms could be from peers. **"I found hundreds of other people that were saying exactly the same that I was, having exactly the same withdrawal symptoms as I was**, and even when I printed that out and took it with me I wasn't believed" – Harriet […]<br><br>A survey in New Zealand reported that a higher proportion of individuals who tapered gradually discontinued completely (Larsen-Barr et al., 2018). A follow-on qualitative study suggested that alongside tapering, an individual's psychosocial strength and available support impacted the outcomes (Larsen-Barr & Seymour, 2021). […]<br><br>As with previous research, the findings showed **a particular value of being connected with other people with shared experiences via online user-led groups** (Geyt et al., 2016). A common reason for accessing peer support is subsequent to failed doctor or psychiatrist supported tapers (White et al., 2021)	Via Personal Networks
(Yeisen et al., 2017)	Most patients sought information about AP on their own, in most cases using online information sources. This approach was perceived to reduce stress, **increase control** and gave a perception of involvement and influence with regard to the process of initiating AP. In most cases, such approach increased adherence.	Post-2014 5*

CMOC 7 ('Meds man continuity positive')

When an individual with SMI on medications has continuity over time in a trusting, repectful therapeutic alliance with practitioners who openly discuss and make collaborative medication decisions with them, even when there are disagreements (C), they are more apt to confide in and to negotiate with their practitioners about their medication issues and management plans (O), due to a sense of safety with their practitioners (M), and increased belief (M) in themselves to manage their lives.

CMOC based on data extracted from 32 documents.

**Document (author, date)**	**Extracted data**	**Subset of literature**
(Carrick et al., 2004)	**There is a spectrum of management of treatment from `control in the hands of others' which the patient is not happy about, through willingly deferred control to the patient being in control**. While many take an active role in negotiating their treatment to maximize their Well-being, doctors have the power to prescribe and the power of knowledge. How active the role of `patient' is depends very much on the individuals concerned. This unequal relationship can work in different ways, which are viewed more or less satisfactory by the patient. **In doctor-patient relationships viewed positively by the patient, phrases like `we decided' and `we had a good result from that' are found, as well as `I agreed with . . .' and `my doctor agreed with me', showing a sense of self as an important negotiator**. […]<br><br>A coping strategy used by those who like to have more control over their **treatment was to consider themselves as `experts', both in terms of having a wealth of experience and because they had sought out knowledge.** The dynamics of the patient-doctor relationship in managing treatment depend on the patient's willingness and ability to be an active partner and the doctor's willingness to allow this. […]<br><br>**A more holistic view of patients and a long-term view of treatment may encourage more effort in deciding upon treatment with which the patient is happy. By negotiating with patients about which side effects and symptoms impact most on their Well-being, a treatment plan can be developed in co-operation, addressing both medical and psychological needs. This approach will naturally lead to higher levels of adherence.**	Pre-2014
(Green et al., 2008)	Qualitative data showed that **positive, trusting relationships with clinicians, developed over time, aid recovery. When "fit" with clinicians was good, long-term relational continuity of care allowed development of close, collaborative relationships, fostered good illness and medication management, and supported patient-directed decisions**. **Most**	Pre-2014

	**valued were competent, caring, trustworthy, and trusting clinicians who treated clinical encounters "like friendships," increasing willingness to seek help and continue care when treatments were not effective and supporting "normal" rather than "mentally ill" identities.** Statistical models showed positive relationships between recovery-oriented patient-driven care and satisfaction with clinicians, medication satisfaction, and recovery. **Relational continuity** indirectly affected quality of life via satisfaction with clinicians; medication satisfaction was associated with fewer symptoms; fewer symptoms were associated with recovery and better quality of life. […]<br><br>Participants also talked often about the importance of **being able to trust their clinician and, equally as important, to have their clinician trust them. Such trust was developed in the context of long-term relationships and could provide important boosts to self-esteem and feelings of self-worth**. […]<br><br>Another theme stressed by many participants was the **importance of having a collaborative relationship with their clinician and, as part of that relationship, having some control over treatment decisions.**Participant: Actually, I just met her recently in the last month, and so far out of all of the doctors that I have seen here she seems to be the best. She is the most informed, the most willing to talk and work with me and what I want. Of course, this is what she wants me to do. We work together. I think she's really good so far. Interviewer: So…working together to figure out what's going to be best for you has been helpful? Participant: Uh huh. Then I get to keep some of that control. [..]<br><br>**Participants reported that seeing the same clinician over time** provided the historical background and experience necessary to get good care and to form a good working partnership. I think the main reason I've stuck with him [for 25 years] is because I tend to be someone that if I find something that works…then I'll stick with it, and **there's a lot to be said for having a provider who knows your history, knows where you've been. …He definitely always lets me share my ideas, and his approach is we work things through together and he listens to me, so…that's the reason I stuck with him**. […]<br><br>Interviewer: How has your mental health care…affected your progress toward making your life what you want it to be? Participant: It helped me feel good about myself, especially with Doctor [NAME] because he talked to me as an equal. He didn't talk down to me. **He didn't talk to me as if I were some kind of animal. He discussed the medications with me. He trusted me. He trusted me to medicate myself or to make a choice. He listened to me. Any questions that I had, he answered me openly and truthfully. He didn't beat around the bush about anything. Anything that I would ask point blank, he would answer me. I'm really impressed with him as a doctor. I like him** […]	

	**He actually makes me feel like part of the decision**, as far as what treatment to proceed with. If I feel that I want to try something else to improve conditions…he may give me some suggestions and he has always said, "Do you want to try to do this?" He says, don't worry. If it doesn't work we can do this and this and the other, but do you want to try it first this way?	
(Matthias et al., 2012)	**More common was negotiation to arrive at a mutually agreed upon decision. These instances were characterized by both parties openly sharing opinions and/or concerns. Negotiation occurred most frequently in medication discussions. Just over a third of all medication decisions were arrived at through a process of information sharing and expression of treatment preferences**. […]<br><br>Providers initiated the majority of decisions. Often, however, the initiation included an invitation for the consumer to offer an opinion, rather than a direct expression of the provider's own preferences. Invitations—**directly requesting input from the other person— appear to be one way to involve consumers in decision making.** […]<br><br>**We observed the greatest amount of discussion and negotiation for medication decisions**. This observation is consistent with the notion that multiple medication options are often available, and psychiatric medications carry varying degrees of risk and side effects that should be discussed with consumers to assure the medication is the best fit for the consumer (Lehman et al., 2004; Hamann, Cohen, Leucht, Busch, & Kissling, 2005). […]<br><br>Providers may view SDM as time consuming, and thus not feasible (Legare et al., 2010), and some research outside of mental health supports this view (Charles et al., 1999; Gotler et al., 2000). However, Loh and colleagues (Loh et al., 2007) conducted a shared decision making intervention in primary care with individuals who were depressed and found that **sharing decisions did not require additional consultation time. In our own data, consultations in which the consumer initiated the decision making process, (i.e., those with the greatest degree of consumer involvement) were very close in length to consultations without consumer-initiated decisions**, although our sample size was relatively small.	Pre-2014
(Quirk et al., 2013)	Our earlier study [6] found that, during outpatient consultations, psychiatrists rely heavily on patient self-report and accounts from informal caregivers (typically a spouse or relative) and other professionals to monitor adherence and attempt to **create a safe conversational environment to facilitate disclosure of information**; for example, by indicating that non-compliance is normal and that reporting it would not be followed by disapproval. […]<br><br>4/22 reports of partial/non-adherence are elicited by a direct question about adherence and a further 5/22 are prompted by a more general enquiry about the patient's use of medication. However, in 7/22 cases the patient volunteers the report of poor adherence without a prompt or targeted question from the doctor.[…] **This analysis**	Pre-2014

	**indicates that patients as well as doctors work to create a safe conversational environment in which to talk about this potentially difficult** issue.<br><br>Patients therefore use two main methods to **create a safe conversational environment** in which to talk about not taking medication. The first is to 'own up' to partial or nonadherence before being asked about it; the second is to present the report in such a way that it makes it difficult for the psychiatrist to respond to it in a disciplinary manner. […] The most common proximal outcome of an adherence exchange is a change to the patient's prescription (9/22; see Table 1). These resulted from the psychiatrist either agreeing to re-align the prescription dosage with what the patient is reportedly taking (e.g. formally ratifying a reduction), or **negotiating a change** in the prescribed medication to something the patient is more likely to take, (e.g. swapping antipsychotics). […]These meetings are generally part of a **long-term therapeutic relationship.** Patterns of interaction are sometimes repeated over time. […]<br><br>In outpatient consultations, psychiatrists rarely acted in a disciplinary way with patients about partial or non-adherence. As we have demonstrated, this can be explained partly by the skilful interactional work done by **both parties to create conversational safety** […]<br><br>**The experienced psychiatrists involved in this study worked together with their patients to construct a safe conversational environment to discuss non-adherence** with antipsychotics, hence allowing the free volunteering of information. […]<br>Reports of partial/non-adherence were not dealt with in a judgemental manner and prescribing was often modified as a result, with even the endorsement of the non-adherence resulting. **Psychiatrists responded in a way to maximise the engagement of the patient and preserve the therapeutic alliance**, even if the acceptance or endorsement of partial/non-adherence sometimes appeared to increase the risk of relapse.	
(Rungruangsiripan et al., 2011)	**Furthermore, the participants in this study had been treated for a long time by their psychiatrists. They reported high alliance with their psychiatrists. They discussed their distress about medication side effects with their psychiatrists, and the psychiatrists adjusted the dose of medication for them. When the psychiatrists adjusted the dose of medication for the patients, the side effects of medication could be reduced or managed. This condition could help foster the patients' positive attitude toward treatment and toward the health care provider**. Moreover, experience of medication side effects provided a topic of discussion for patients and their psychiatrists. All of these processes enhanced their understanding of illness patterns. This finding also emphasizes the important role of the health care provider in helping patients manage their side effects.	Pre-2014
(Seale et al., 2006)	Because thinking in terms of patient compliance or non-adherence has been criticised, on the grounds that this involves unduly blaming patients whose own expertise and rationality goes unrecognised, many therefore now	Pre-2014

	advocate concordance as a model that is more in tune with current views (Bissell, May, & Noyce, 2004). **Concordance** involves mutual respect and understanding in pursuit of an ideal therapeutic alliance (Working Party, 1997). **Decisions about treatment are then shared by all parties in the relationship, including healthcare providers, patients and any third parties such as relatives (Charles, Gafni, & Whelan, 1997, 1999).** […]<br><br>**It entails an implicit view of 'non-compliance' as a potentially reasonable response to breakdowns of concordance**, a view to which medical anthropologists and sociologists studying patients' experiences of treatments (including medications) have made important contributions (Britten, 1994; Conrad, 1985). […]<br><br>Thus, advice exists in the psychiatric literature on how to build a shared alliance with patients diagnosed as having schizophrenia (Bhugra & Holsgrove, 2005; Piatkowska & Farnill, 1992), **advocating respectful exploration of patients' feelings about their illness and treatment, and negotiation of treatment intervention.** […]<br><br>**A preference for a co-operative relationship involving shared decision making, choices that reflected patient's wishes, negotiated agreements and a sense of partnership, was described by 14 of the psychiatrists:** I genuinely see us as in it together, if you like, that it's a partnership and it's not going to work if I am somehow in a position of superiority and they're in a subordinate subject position. I think it just won't work. (Interview 19) **A number of ways of achieving this were described, including listening to people's views about their situation, their illness and their medications (9 doctors), showing empathy, understanding, warmth, encouragement, respect or closeness (8 doctors) and using language carefully and tactfully, often using non-technical words and explanations that a patient could understand, or reflected the patient's way of seeing things (7 doctors**). […]<br>The benefits of knowing someone **over a long period of time were described** by six doctors and three mentioned the need to give people time to process and respond to information before reaching decisions.	
(Clifford et al., 2020)	The most common service-related factor influencing medication adherence was the relationship between the consumer and the prescriber. "You've gotta have a good relationship with your psychiatrist. Otherwise, you're not gonna get anywhere. Well you can, but it's not very pleasant. When you go there, you wanna be able to feel heard and understand what they're doing and what needs to be done." **Interviewees were more positive** about medication adherence when they **perceived the therapeutic alliance to be collaborative, where treatment decisions were shared**. […] Seeing a new prescriber "every six months" (due to the structure of the psychiatry training program with six monthly rotations) affected medication adherence as it acted as a barrier for patients to confide in new clinicians: "Every six months you swap and you get somebody new and it's like, when you start to feel comfortable and talking to 'em, they change it".	Post-2014<br>4*

(Delman et al., 2015)	"Active" participation has been defined in several ways in mental health treatment.9 This definition is drawn from the work of Finfgeld, who **defines active participation as utilizing one's own capacity (knowledge, skills, and beliefs) to exert direct influence over decisions about his/her treatment.10** Finfgeld identifies two broad levels of active participation.9,10 At the "Choosing" level, the client expresses feelings about his/her treatment options and/or assertively selects from them. A higher level of participation is "Negotiating," by which the client is able to not only take a position and express a treatment choice but also to reach a compromise with the provider who may start from a different position. This fourth level is characterized by **a greater sense of mutual respect between the client and provider, and requires a heightened degree of effort and skill from both parties.** [...]<br><br>**Respondents reported that their current psychiatrists expressed an openness to their perspectives on treatment**. These psychiatrists demonstrated good listening skills, though not all of them made it a practice to ask directly for the client's opinion. As one respondent noted: "She doesn't treat you like a little kid, um, who doesn't really know anything. She treats you like an adult. She gives you the impression that it's important to her that she listen." The respondents' assessments here were buttressed by **an existing trusting relationship with the psychiatrist, whom they respected and liked. At times, the psychiatrists may have disagreed with the client's choice, but ultimately respected his/her right to make the treatment decisions**. They didn't just abandon the client, but worked with them as best as they could. According to one respondent: I wanted to go off meds. She said she didn't want to do it but **it was my decision and she'd help me do it a way that would be most effective, and we can catch anything if I have an issue.** She told me to call if I was having any delusions to call the crisis line here; she wanted to make sure that nothing bad happens, or something, we can stop. [...]<br><br>Those who at times negotiated with their psychiatrist to an acceptable solution reported high levels of confidence. **The actual act of "negotiating" in ways they had not previously raised their confidence level, as well as the "personal growth" reported in the previous section.** As one respondent noted: I was getting to know him and started to feel more comfortable talking about side effects as they occurred. He was OK with that, and we made changes together so I wouldn't have those problems. I became more confident in myself and had faith in myself. Instead of being scared and worried, I would speak up when he recommended a medication change. Sometimes I didn't like the side effects he mentioned, and I now say something, where before I wouldn't have said much**. Growth in confidence also appeared to relate to a respondent's gaining control over his/her life generally, even while struggling with illness** [...]<br><br>With early opportunities to learn about their role as responsible adults, these young adults may experience "personal growth" or maturation.5 The best opportunities for growth present when they **find psychiatrists who reduce professional boundaries, engage in respectful and caring communications, and encourage the client to participate as**	Post-2014<br>4*

	**collaborator.8 A by-product or component of these respondents' improved self-efficacy was their achieving greater control over their life and psychiatric condition.1** […]<br><br>Psychiatrists encourage activation by their willingness to reduce the relationship's power imbalance and by relinquishing control in order to compromise.10 As broadly stated by Finfgeld, this is not a light undertaking: "Health care providers are urged to accept the trial-and-error approach, provide meaningful feedback if needed, and be prepared to rescue clients when necessary."10, p. 47 Training, education, and mentoring for psychiatrists on incorporating client preferences in decision making should include approaches to taking and expressing an interest in their client's life, **encouraging the client to take an active role in treatment, and negotiating with the client when there is a disagreement**	
(Fiorillo et al., 2020)	In recent years, the process [SDM] has been gradually remodeled to become a more patient-inclusive approach letting the voice of those directly affected by the decisions to be heard [2]. In such spirit, the clinician empowers the patient to take part on his/her own treatment strategy by providing the patient clear and exhaustive medical information, while listening to the patient's preferences and priorities and facilitating the patient's evaluations towards a balanced reasoned decision. **It is a negotiation between the clinician and patient taking place for achieving a shared decision [3**] In the general medical setting, the type of decisionmaking is influenced by the balance achieved in the clinician–patients relationship and positions itself along a continuum, ranging from the paternalistic (clinician-led or passive style), through shared decision-making (SDM), up to the patient-led active style (also known as informed style) [4]. […]<br><br>**With specific reference to the SDM style, studies have demonstrated that it has a positive impact on the patient's levels of satisfaction and adherence to treatments, as well as on his/her quality of life and empowerment [**7]. This has been especially highlighted in the case of patients with severe mental disorders who report a greater desire of being involved in clinical decision-making and a need to have a say in the process of care, as compared to individuals receiving assistance for other medical conditions [8–11]. Patients and clinicians bring different—but equally important—knowledge and expertise to the decision process, which need to be integrated [12]. **When patients are involved in choices about their own health and care, they ponder options carefully and are most likely to appreciate the value of proposed treatment, to agree to treatment with a favorable attitude. In fact, shared process has proven to increase adherence to the prescribed treatment and improve long-term outcomes.** Furthermore, this has also translated in more efficient allocation of healthcare resources [13–15]. […]<br><br>As suggested by NICE guidelines, clinicians should negotiate with patients and their carers as early as possible on how information will be shared [27].	Post-2014<br>4*

(Garcia et al., 2016)	Therapeutic alliance has also been identified as a relevant factor for improving adherence to antipsychotics.[17,54,63] A study on patients with bipolar disorder found that patient collaboration was significantly associated with good adherence, that is, patients **being involved as a comanager of their own illness, with the psychiatrist considering their opinions and comments during the intervention process, helped improve the management of the illness, and hence led to better treatment adherence.**[64]	Post-2014 4*
(Jawad et al., 2018)	In terms of improving adherence, assessment of a patient's understanding of their diagnosis, symptoms and the potential risks associated with stopping medications, is likely to be critical. The communication style of the doctor is important to their ability to forge a therapeutic alliance. **Alliance may be weakened when a patients' care lacks clinical continuity, for exampl**e, they see different doctors at different appointments or receive their care from different teams at different time points, for example, the community mental health team, inpatient team and crisis home treatment team. Sometimes the involvement of multiple teams is unavoidable, and it may carry some advantages for example in terms of specialization and greater input at a time of high clinical need. However, services need to ensure a seamless transfer between teams with timely handovers	Post-2014 4*
(Kaar et al., 2019)	Others [SUs] found this kind of information [from other sources] neither helpful nor engaging. In these cases, the doctor's professional expertise was valued above all else, and the **rapport built over time trumped any external input**.	Post-2014 4*
(Leclerc et al., 2015)	In recent publications, **treatment engagement has been emphasized in addition to adherence to medication, since, alongside shared decision-making, it can provide better quality of life and beneficial lifestyle changes**, such as regularity of habits or reduced drug and/or alcohol use.27-29	Post-2014 4*
(Lim et al., 2021)	Studies have reported therapeutic alliance between psychiatrists and patients in the outpatient setting[17] and in the inpatient setting[19–21] as a reliable predictor of adherence. […]<br><br>**Therapeutic alliance was identified as a key determinant of drug attitudes in individuals with psychosis. In addition, individuals who identified psychiatrists as the health care professional with whom they had the strongest therapeutic alliance reported more positive drug attitudes toward psychiatric medications**. […]<br><br>A qualitative study supports this as patients had identified doctors being understanding and listening to their concerns as the reasons for adherence.[70] Thus, having an affective bond with the psychiatrist would likely contribute to a much more positive drug attitude.	Post-2014 4*
(Mahone et al., 2016)	Decisions about initiating, titrating, and switching medications involve **ongoing discussions during clinical consultations and are part of learning to self-manage a mental disorder**. […]	Post-2014 4*

	Recovery—a process of change through which clients improve their health and wellness, live a self-directed life, and strive **to reach their full potential—is usually accomplished through a combination of personal empowerment, a sense of responsibility, choice, and active self-help (Substance Abuse and Mental** Health Services Administration, 2012). […]<br><br>Selecting appropriate medication and behavioral therapies using shared decision-making is one strategy that may improve treatment follow-through (Haddad, Brain, & Scott, 2014). […]<br><br>Shared decision-making is a "process of enabling clients to participate actively and meaningfully in their treatment by providing them with accessible information and choices" (Adams, Drake, & Wolford, 2007, p. 1219). **It is a model that allows clients and providers to collaborate to assess treatment risks and benefits as part of a treatment plan** (Deegan & Drake, 2006). Shared decision-making empowers clients with the knowledge needed to make informed decisions together with their treatment team and to engage as active participants in their treatment plan (Mahone et al., 2011). […]<br><br>Gauging the client's initial preference for and ability to engage in shared decision-making is helpful in beginning the provider-client communication about medication treatment options. Because preferences and ability to engage may change throughout the course of treatment, this preference and ability should be reassessed at follow-up appointments (Wills & Holmes-Rovner, 2006). […]<br><br>Before discussing new treatment options and making a treatment decision, Sam was queried about the degree of involvement he would like in making decisions about treatment and services. Sam indicated that he would like to be involved in most decisions, but he also wants some assistance and reassurance as requested when making decisions.	
(Maj et al., 2021)	Over time, patients (and their environment) learn about their mental vulnerability, the relativity of formal diagnosis, the limitations of treatment, the gaps in knowledge, and the weak spots in local service provision. As a result, they become more involved in and opinionated about treatment and services88, so that the process of shared decision-making becomes even more essential. It is therefore important to assess, before planning the clinical management, the preceding course of decision-making about diagnosis and treatment, and the experience to date in being able to experiment with dosing and even discontinuation of antipsychotic treatment, to engage in alternative therapies and in general to take risks in pursuit of life goals. In order to be able to deal with an intense mental vulnerability, characterized by an often unpredictable waxing and waning expression over time**, a long-term therapeutic relationship of trust and mutual commitment is essential.** […] …the management of primary psychosis should be recovery-oriented; that it should take into account the patient's practical needs; and that **the management plan will**	Post-2014<br>4*

	**have to be agreed upon between the clinician(s) and the patient. It is indeed not common that a resilience-promoting therapeutic environment and a focus on empowerment**, identity, meaning and resilience is ensured in ordinary practice; that patients' needs in terms of employment, housing, self-care, social relationships and education are taken into account in the management plan; and that negotiation and shared decision-making are really implemented.	
(Aref-Adib et al., 2016)	Previous qualitative research from EIS has highlighted the value that patients place on being involved in treatment decisions and working jointly with clinicians on their care plans [17]. Perhaps greater **openness and equality in EIS therapeutic relationships has facilitated sharing and discussion regarding patients' online mental health searches**.	Post-2014<br>5*
(Bjornestad et al., 2020)	Here, reducing side effects through either dose-reduction or through manipulating the time points for when the hardest side effects were hitting – e.g. taking drugs in the middle of the night instead of in the morning – were seen as crucial. This usually involved some experimenting alone and in dialogue with professionals. […]<br><br>Also, in the longer term, patients saw it as essential that **communication was reciprocal, respectful, and involved a high degree of user involvement both in treatment planning and treatment delivery**. […] Moreover, patients preferred professionals to **view recovery as an individual matter** and to appreciate that antipsychotics were one of many tools and not necessarily the main ingredient in recovery. Bjornestad et al., 2020, p. 518	Post-2014<br>5*
(Bolden et al., 2019)	In treatments for chronic conditions, **participation in decision making is more fruitfully viewed as a process which may unfold over the course of an encounter, or even multiple encounters** (**S**ay et al. 2006), and, in the case of psychiatric disorders, is inextricably tied to the context of an on-going therapeutic relationship (Matthias et al. 2013).	Post-2014<br>5*
(Crellin et al., 2022)	**Routine discussions with** long-term patients may reveal people who are dissatisfied with their current treatment for a variety of understandable reasons that are important to explore. Some people who stop medication abruptly and potentially dangerously on their own might cooperate with a planned strategy of supervised reduction conducted in collaboration with their psychiatrist. Yet other patients may wish to switch to a different medication to lessen the impact of specific adverse effects [49]. […]<br><br>Support from healthcare professionals was identified as being helpful in attempting to reduce or discontinue antipsychotic medication, as was gradual reduction (cited by 25% of patients). This coincides with recent literature on the importance of gradual tapering when reducing or stopping antipsychotic medication to minimise the risk of relapse [45]. Wanting to be independent was regarded as an important motivation for trying to reduce or stop medication. Crellin et al., (2022, p. 8)	Post-2014<br>5*
(Ehrlich & Dannapfel, 2017)	They believed that they **could talk openly with the doctor about their** problems. Participants highlighted that they wanted their concerns to be taken seriously, and that GPs would act to uncover whether or not there was a physical	Post-2014<br>5*

	basis for their concerns.	
(Grunwald et al., 2021)<br>(Role of hope and trust)	**Increased trust has been associated with a better therapeutic alliance** [43, 54, 60, 61, 82]. Given that there are multiple types of antipsychotics and dosing options, varying responses to antipsychotic medication, and no guidelines on how to review and reduce medication [16], GPs and SU encounter many uncertainties. Managing these uncertainties together requires a trusting relationship between GP and SU [60, 61]. Any history of coercion or sectioning under the Mental Health Act can make developing and maintaining trust more difficult, but a **trusting relationship is key to shared decision making** [29, 82]. […]<br><br>**Continuity of care** has been highlighted as a crucial factor for this population [54, 82] as it can help GPs to potentially spot signs of relapse early and offer appropriate support, and is likely to be an important factor in facilitating a safe process of medication change. Continuity may also increase SU trust and encourage the start of conversations about medication.	Post-2014<br>5*
(Haddad et al., 2014)	The amount of information required will vary between patients. Where possible, a choice of medication should be offered. […] It is important that patients are warned of side effects before starting medication. Tolerance develops to some side effects. Titrating the dose upwards gradually and/or explaining to the patient that side effects should settle can reduce the likelihood of side effects impairing adherence. Managing side effects that emerge during treatment depends on their detection at review appointments. **If patients have a good therapeutic alliance with the clinical team, particularly the prescriber, then they are more likely to volunteer side effects.** […] …shared decision-making about medication may be undermined if a patient has brief interactions with many professionals, who may themselves hold differing views, rather than a close working alliance with selected individuals.	Post-2014<br>5*
(Hansen et al., 2018)	An important implication of our findings is therefore the need for clinicians and researchers to attend to both the benefits and risks of treatment with anti-psychotic medication (Harrow & Jobe, 2013; Wunderink, Nieboer, Wiersma, Sytema, & Nienhuis, 2013) and the importance of collaboration between service providers and the people using the services when it comes to decision-making regarding medication in early psychosis (Geyt, Awenat, Tai, & Haddock, 2017).	Via Citation Search
(Geyt et al., 2017)	Navigating the responses of others played a key role in participants' efforts to manage safely.<br>Trusted prescribers, therapists, family and other people with personal experience held valuable roles in people's efforts to understand themselves and meet their needs.<br><br>When I raised stopping the antipsychotic] she said, oh, that sounds wonderful. Let's go for it. [. . .] She had drawn diagrams and told me to shave bits of pills off and all that sort of thing. [. . .] She planned it all out and wrote it all out and spoke to me about it. And said, don't rush it, there's always a temptation to rush it but don't. [. . .] She was also someone you could email if there were any problems with it. Actually at that time when we were trying to do that, she	Post-2014<br>5*

	also sent me off to a psychologist. –	
(Katz et al., 2019)	The current study results are consistent with previous studies, which showed that the experience of medication usage might interfere with one's daily living and create "Medication-Related Burden" (MRB; Mohammed, Moles, & Chen, 2016). Naturally, when the benefits of medication are no longer perceived as outweighing the MRB, then doubts about continuing to use it are inevitable. **These doubts can be experienced in isolation or, preferably, may be shared and discussed with care-providers and natural supports. Obviously, alliance and trust are crucial for a meaningful and constructive dialogue to take place with the care team.**	Via Citation Search
(Pinfold et al., 2019)	Whether a shared or independent approach was described seemed strongly influenced by the women's experience of working with their clinical team. **Accounts of shared decisions** were characterized by active involvement of both the women and their clinical team, and in many cases their wider family. **Where continuity of care was reported, and established relationships were built upon trust, the accounts were more likely to describe a supportive process**. Open discussion was found in accounts where alignment of views between the women and clinician were strong, including acknowledgment of uncertainty: So we'd, prior to me getting pregnant, looked in depth at sort of what I should do, so I'd already made the decision [to stay on a specific medication]. (Emma) […]<br>**What was crucial to the women was the health support that clinicians could provide during the decision-making process, and the need to keep that support during and after pregnancy regardless of decisional (non)congruence**. The process of decision making and the level of agreement reached with clinicians should not affect women's rights to access high standards of care. […]<br><br>Shared decision making also involved active negotiation, particularly to bring the clinical team onside with personal preferences. Negotiation was found in accounts where medication changes were required both in order to become pregnant and manage the risk to mother and baby during pregnancy:<br>**I feel very fortunate that the psychiatrist was prepared to negotiate on [which medication to take] […] you know, I would take research papers along and say you know what do you think about this and how about that?** (Grace) The crucial thing, I did have a mission, I have to be honest, because I started negotiating with the psychiatrist before my husband had agreed to it [having a baby]. (Agatha) […]<br>The accounts all described the influence of clinical relationships on decision making, both mental health and general health care practitioners**. […] Reaching agreement on the decision with clinicians was, in most cases, helpful in achieving effective planning and support throughout the pregnancy and after childbirth. Close monitoring throughout the pregnancy provided the possibility to change an initial decision, as it did for Grace:** And because my health had been previously quite good, or had been good, the psychiatrist was happy for me to come off the medication during the process of trying for a baby with the recognition on both sides that if – both his side, as was then, and my side – that if things changed, if I became unwell, then we would re-visit the situation. […] So we agreed to	Post-2014 5*

	do a close monitoring, and effectively I kind of weathered the low for as long as I could but then it sort of got a bit worse, to the point where I had to take a little bit of time off work, at which point I said, OK, hands up, I need something. (Grace)	
(Sowerby & Taylor, 2017)	Engagement in shared-care clozapine with a relationship demonstrated by the Forensic HCP/CSU partnership provides an example of a shift in care from paternalistic to person centred. We suggest that shared-care clozapine in this context offers an opportunity to demonstrate dignity, respect and compassion to CSUs **through trust and handing over greater responsibility to the CSU**. The provision of coordinated care and support through the development of relationships between multi-professional team members in primary and secondary care enabled personalised care and support for CSUs, which was subsequently reflected in CSUs **developing their ability to manage their own role in shared care.** This person-centred approach ultimately enabled CSUs to live an independent and fulfilling life by self-managing their own clozapine.	Post-2014 5*
(Steingard, 2018)	It was suggested that patients reduce their doses by no more than 25–30% of the initial dose at intervals no more frequent than every 3 months. The rational for this recommendation was based on data from tapering studies where patients were followed for up to 2 years. Most relapses occurred within 3–6 months (Viguera et al. 1997). In addition, a slow taper was hypothesized to reduce risk of supersensitivity psychosis which could happen as a result of antipsychotic induced up regulation of the post-synaptic dopamine receptor (Chouinard et al. 2017). The goal was not necessarily to stop the drug completely but to establish a new minimally effective dose (MED). Patients who were more cautious could reduce at a slower rate. If a patient was able to remain stable for 3–6 months after dose reduction, it was assumed a new MED was established. **Decisions were made in a collaborative way and patients were free to stop or begin the taper at any time**	Additional search (tapering)
(Watts et al., 2021)	In many cases, they[SUs] are looking for nurses for advice and support in relation to their desires to discontinue medication. Furthermore, understanding people's experiences is important if the aspiration of delivering a service that embraces the concepts of clinical recovery and personal recovery (Chester et al. 2016; Stickley et al. 2016) and one that is **focussed on partnership approaches to care, and people's rights to make informed choices about their care and treatment** (Higgins & McGowan 2014; Leamy et al. 2011). […]<br><br>Where support was not forthcoming, participants either decided to 'go it alone' or, where possible, switched care to a psychiatrist or GP who they believed would be supportive of their decision. **Psychiatrists and GPs who offered support did not necessarily share participants' views; however, they were open to listening, discussing the idea and making a plan with the person.** He mightn't agree with me but he collaborates.. ..I would hate to be in a position where I'm arm wrestling my doctor,...or coping with resisting his pressure. [P 18; Male] […]<br><br>**… engaging in a collaborative discussion may support safer decision-making, and lessen the risk of people**	Via Citation Search

	**discontinuing medication abruptly and learning through trial and error. It also offers the person the opportunity to discuss strategies** such as changing medication to those with a shorter half-life or changing to a liquid form of a drug, which **gives both the person and team greater control over reducing the dosage slowly and safel**y (Darton 2016; Hall 2012), which is important given the potential relationship between the pace of discontinuation and relapse (Viguera et al. 1997). […]<br><br>**To enhance safety of practice, nurse need to provide people with the best evidence available in relation to discontinuation and support people to move from covert actions to articulate openly their preferences and priorities around medications** to the multidisciplinary team, even if those conflict with professional perspectives. Without this people may put themselves in greater risk by attempting to discontinue medication abruptly and without developing strategies to scaffold and protect their well-being and safety in the discontinuation process.	
(Weiden, 2016)	Most of the time patients want to feel better and do better, and most clinicians want their patients to achieve the best outcomes possible. If there are differences in how to achieve those goals, it does not automatically follow that it has to be a source of acrimony, alienation, or mistrust. **Most patients, if given the chance, feel better when they feel safe to disclose their true feelings about the difficulties posed by their condition**. They would feel a sense of relief if their clinician could really understand how disappointing and frustrating it is to have to take medications that do not fully restore the individual back to his or her premorbid condition. Most patients would find it liberating to feel like they are allowed to openly discuss ambivalence to medication without getting cut off midsentence to be told to stay on medication. Active listening without interruption will make it easier for patients to articulate concerns that are counterintuitive but important. […]<br><br>**Most patients would like to be honest with their clinicians and feel safe that they will not be thought of as a "bad patient" if they admit to stopping medication**. Most patients will find it reassuring when their clinicians provide guidance as to how they can feel better, even if that guidance is not immediately accepted.46 **Differences of opinion that are respected are easier to accept than when verbalizing those differences leaves someone feeling chastised.** Clinicians can learn that listening and acknowledging feelings does not represent colluding or abandoning the process of making strong recommendations with conviction. Acknowledging the obvious, that treatment recommendations are often not followed, does not mean resignation or willingness to accept suboptimal outcomes. The central thesis is that clinicians are better off realizing that the road to improving adherence is often through the therapeutic relationship. […]	Post-2014<br>5*

	Patients with schizophrenia may be relatively susceptible to social desirability factors ranging from passive acquiescence to fear (real or imagined) of disappointing their treatment team (eg, being thought of as a "bad" patient). Also, patients with schizophrenia may reasonably fear real-life consequences of disclosing nonadherence, such as hospitalization or losing housing benefits. **The goal is to help the patient understand that accurate reporting of medication status is a safe harbor and hopefully dispel anxiety or wariness**. […]<br><br>Clinicians who wait for their patients to take the initiative to disclose nonadherence often have to wait for a long time. Disclosing nonadherence can be stressful, and may be easier to respond to a gentle question than bringing it up without prompting. The discussion can be prefaced by reminding the patient about the importance of having accurate information about the medication regimen as taken; for example, "You know, I know what I have prescribed but I don't know what you've actually taken. It is really important for your care that I really know what's been taken, which makes it safer for you and more likely that I will 'get it right' for medication adjustments later on." **Afterward, the most important point for follow-up questions is that it is emotionally safe**, **with gentle questions and reassuring tone, for the patient to respond honestly**	
(Kaminskiy et al., 2021)	Service users, psychiatrists, and nurses viewed SDM in terms of encouraging service users to have **increased say over decisions concerning medication, and promoting ownership and self-determination in meetings concerning medication. All three groups strived toward the service user achieving greater self-management skills.** […]<br><br>Establishing a long-term partnership and supporting people with their personal recovery journeys was seen as integral to the process of SDM for CPNs and SU participants. **CPNs referred to the importance of a supportive, long term relationship with SUs. Care and empathy is emphasized reinforcing a deeper level of connection as a way of facilitating a collaborative partnership.** Both groups saw this as a continued reflective process, of being held accountable, pushing forward and having a belief in one's potential during more difficult times, and celebrating, and reflecting on success over time. **For SUs, meaningful involvement was not seen as a series of isolated decisions, but instead, a practitioner seeing and commenting on change over time was connected to feelings of being known and cared about**	Via personal network

CMOC 8 ('Perceived risks')

When individuals with SMI desire to taper, change or discontinue their medication regimen (C), their clinicians may resist sharing information with them and may not support them (O) because they judge that doing so may put themselves, the service user and others at risk (M) if adverse outcomes occur (e.g., harm to self or others).

CMOC based on data extracted from 11 documents.

**Document (author, date)**	**Extracted data**	**Subset of literature**
(Opler et al., 2004)	The clinicians in the [Tavares Hispanic]clinic had previously downplayed or been vague about the possibility of side effects because we had incorrectly assumed such discussions would add to fear of medications. **We had also incorrectly assumed that this population might be particularly prone to suggestibility and that discussing side effects might induce them.**	Pre-2014
(Seale et al., 2006)	Fifteen mentioned aspects of communication about adverse effects that were difficult and seven expressed concern about the negative impact of information about these effects on patients' motivation to take medications. Additionally, eight doctors mentioned difficulty in providing comprehensive or precise information about adverse effects, either because they found it hard to know these themselves or because it was impractical to discuss every single one, including those that might be extremely rare, in the time available. **That this was a problem was indicated by the fact that several linked this anxiety to the potential for later complaint or litigation from patients** affected by harmful effects they had not been warned about. I am generally fearful that I don't give people enough information. And we live in a litigious world, and I don't know any doctor who is not worried about it. It is absolutely impossible to give them every bit of information. (Interview 21). […]<br><br>A central dilemma, then, lies in the fact that on the one hand, doctors were largely convinced about the value of antipsychotic medications, but on the other hand**, were worried about the consequences of fully explaining adverse responses. Some appeared to solve this by being economical with the truth.** […]<br><br>Nine of the doctors reported degrees of **deception in** their approach to patients. This could range from not telling patients certain things, to saying things that were not true. All of these involved the feeling that the deception was in the best interests of patients. Explanation of adverse effects of drugs, or naming the diagnosis, presented particular difficulties, leading to delays in the disclosure of such information.	Pre-2014
(Fiorillo et al., 2020)	It is clear that in many cases patients being prescribed antipsychotics would need to understand the advantages and long-term positive impacts on their functional outcome, especially in the case of LAIs where this should be discussed as early as possible [26]. Many mental health care professionals consider the matter of medication **to be too sensitive to be**	Post-2014 4*

	**discussed with the patient and approached by SDM, too time-consuming for them and somewhat discouraging for the patient (in terms of adverse effects)**	
(Roberts et al., 2018)	Other participants suggest that while they are happy to hear the patient's views **they will overrule their views if they disagree with what they feel is best**. "I've got more psychiatric experience than the patient has…what I would do here, I would first try Amisulpride" (Participant eight). This participant did not mention guidelines at any point during their interview. Participant ten discusses and follows NICE guidelines, describing the discussion with the patient as a "negotiation". This is important to them as they recognise that many patients do not take the medication that they are prescribed. Other participants disagree; while they believe that the patient should be consulted they think that too much choice could be viewed as uncertainty about antipsychotics: "the patient may be left with the idea that erm, we're not one hundred percent certain, you know, which will be effective and which won't be really" (participant 6).	Post-2014 4*
(Crellin et al., 2022)	**Clinicians can be reluctant to help people reduce or discontinue antipsychotic medication because they are focused on risk**, and lack training and official guidance on how best to do this [47]. This is a concern since patients may sense this reluctance, and may reduce or stop medication without sharing this with their psychiatrists	Post-2014 5*
(Martinez-Hernaez et al., 2020)	Patients were aware that the lack of medical information was due to the **professional fear of jeopardising adherence**. However, they missed a kind of communication that prioritises continuity of care over control, vigilance, distrust and hierarchy. In this sense, they placed a very high value on their relationships with nurses and with the professionals involved in individualised support programmes designed to create a bond of care and trust over time. […]<br><br>The professionals stated that providing complete information to patients about the adverse effects of medication could lead to a risk of discontinuation. According to one professional, "unconsciously, we are all afraid that patients will abandon the treatment" (Professionals Focus Group 1). This **perceived risk** leads to the contradiction between their agreeing to give full clinical information and recognising patients' rights, and not informing, providing only partial information or adopting the logic of faits accomplis.	Post-2014 5*
(King et al., 2022)	Participants in this study felt that **services were risk averse where discontinuation was** concerned, which aligns with other recent findings (Geyt et al., 2016). As found in previous research, **participants experienced relationships with services as conditional on adherence (Bjo**rnestad et al., 2020). A focus on medication adherence is interwoven with underlying assumptions that those choosing to discontinue are predetermined to experience poorer outcomes (Cooper et al., 2019; Larsen-Barr & Seymour, 2021). The high rates of antipsychotic discontinuation without clinical support might therefore be maintained by the current nature of the mental health system. Clearer clinical guidance with specific details on discontinuation and tapering may encourage less risk averse practice among clinicians. […]	Via personal network

	Implications for Practice<br><br>These findings suggest a need for clinical guidelines for the withdrawal of antipsychotics to reduce the confusion caused by contradictory advice given by different professionals and improve the safety of attempts to withdraw. Practice recommendations should follow in the German schizophrenia guidelines (Gaebel et al., 2020) regarding explicit guidance for tapering, acknowledging the risk of withdrawal effects, and reiterating the importance of a stepwise taper. […]<br><br>It is necessary to educate service providers about the variety of motivations for stopping antipsychotics and the realities of the effects of long-term antipsychotic treatment. In addition, it is necessary to address any **blame-culture where clinician-supported discontinuation attempts are unsuccessful and may even result in legal action** (Cooper et al., 2019; Moncrieff et al., 2020).	
(Pedley et al., 2018)	In one study, although professionals acknowledged patients' entitlement to honest information, they also **discussed the need to limit, or even deliberately withhold certain information about side-effects. This was to avoid patients becoming confused, as well as to ensure the best possible clinical outcome**, e.g. telling a young person about the risk of weight gain may discourage them from taking the medication.[48] This raises the prospect that patients sometimes receive inadequate information owing to health professionals' concern about their ability to use this information responsibly.	Post-2014 5*
(Grünwald & Thompson, 2021) (Restarting the conversation)	Clinicians may also not be able to share information. Guidance is lacking on how to antipsychotic medication making open discussion challenging. Additionally, **clinicians may feel pressure to ensure medication adherence, leading to a reluctance to share information regarding potential adverse effects**. **Clinicians may worry about SU refusing or stopping medication they were given all the information.** Therefore, they may not disclose all of the information necessary to achieve true informed consent from SUs (Maidment et al., 2011).	Post-2014 5*
(Kaminskiy et al., 2021)	Many participants referred to not always receiving adequate information. Likewise, most psychiatrists, while stressing the importance of disclosing possible adverse effects, often referred to not doing so due to time constraints and limiting the conversation about what side effects might be important to them. Instead, psychiatrists preferred referring service users to other sources of information, such as leaflets. **In addition, while psychiatrists did not think they would deliberately withhold information on adverse effects, some acknowledged that at times, limiting the discussion about adverse effects and possible benefits of the medication was used as a way (consciously or unconsciously) to encourage concordance and avoid possible conflict (28).** […]	Via personal network

	Psychiatrist 1: And I'm probably not great about telling people about possible longer term side effects about things and particularly anti psychotics I suppose. I guess there are slightly peculiar circumstances, so if someone's psychotic and has lots of delusions and is fairly wound up in them then a conversation about medication can go a slightly odd way and tend to focus on symptoms that might be otherwise quite secondary. Psychiatrist 2: I think I'd usually say the commonest side effects that other people have mentioned to me about medication, but I usually tell them to look it up on the leaflet I provide, or the internet, because there's no way I can go through all the side effects and I don't know which of the side effects might be important to them.	
(Zisman-Ilani et al., 2021)	**Clinicians may resist SDM with patients with serious mental illness because they fear being held liable for any potentially negative outcome** that might result from SDM, such as symptom exacerbation, hospitalization, or death. Burnout, patient load, and limited appointment time also contribute to clinicians' reluctance to engage in SDM (4). […]<br><br>In psychiatry, reducing risks to safety is the highest priority, because it is a key part of one aspect of clinicians' professional training and responsibility and is the common institutional policy among mental health services. Perceived risks to safety affect both clinicians and patients, but differently. **For clinicians, safety risks, such as relapse, present high potential for legal liability, particularly if the risk is accompanied by symptoms potentially related to harm, such as suicidality or homicidality.** For patients, these risks may take the form of reduced quality of life, which might stem from symptom exacerbation, hospitalization, or the psychological or potentially permanent physical side effects of treatment (7). […]<br><br>The recognition of risks in SDM with patients who have serious mental illness calls for a deeper exploration of at least two aspects of the SDM process that have been relatively unrecognized. First, SDM focuses more on presenting medical options and discussing their risks and benefits than on sharing emotions pertaining to risk taking. **We believe that the success of SDM relies on a set of fundamentally intersubjective patient-clinician processes that involve a shared experience of anxiety, worry, and fear when certain decisions are made (11). Patients' and clinicians' expression of both worries and hopes during SDM can serve as a foundation for joint reflection and can lead to a better decision-making process, an informed decision, and a greater commitment and motivation to attain a positive outcome**	Via personal network

CMOC 9 ('Family support positive')

When an individual with SMI trusts family and social network members to believe in them, want the best for them and to provide non-judgemental support (C), they are apt to feel more confident in following through with prescribed medication plans(O) due to a sense of safety (M) among people looking after their well-being.

CMOC based on data extracted from 9 documents.

**Document (author, date)**	**Extracted data**	**Subset of literature**
(Leclerc et al., 2015)	Many studies have addressed the impact of family or social support on adherence, and their results mostly show an association. For example, patients who are nonadherent to medication are less likely to have a family member involved in their treatment.22 In a study of 100 patients, a good level of social and family support at entry was a significant predictor of adherence at 6 months (OR = 3.552, p = 0.03),30 and a large cohort study found a lack of consistent family support to be among the strongest predictors of medication nonadherence at 2 years.45 Likewise, **there is strong evidence that family support and involvement in treatment have a positive impact on engagement.**	Post-2014 4*
(Katz et al., 2019)	**Participants mentioned receiving support during times of crisis from people who believed in them and wanted the best for them. The participants considered this support a crucial component in the medication-discontinuation process.** An analysis of the interviews revealed that **unconditional acceptance and a true belief** in one's potential contributed greatly to the process. **Such dedication and support, which some (but not all) of the participants received, usually came from spouses, professionals, or family members.** […]<br><br>Because of his [her father] belief and support, Sharon could gradually believe in herself, begin to overcome the crisis, and rebuild herself—all without the aid of medication.	Via Citation Search
(King et al., 2022)	Wanting to discontinue was a personal decision and experiences of withdrawal effects were subjective. **When participants shared their withdrawal experiences strategy, symptoms and impact on physical and mental health were discussed. Receiving support formally or informally was an important part of the withdrawal journey for the participants.**	Via personal network
(Larsen-Barr & Seymour, 2021)	All described forming relationships they could turn to for practical and emotional assistance with their efforts to maintain their wellbeing during and after withdrawal. This involved **selectively trusting people to support their mental-health journey and the withdrawal process, connecting with peer support, and building meaningful relationships and valued roles within their families and communities**. […]	Via Citation Search

	**Participants developed an ability to trust people to support them so they were not isolated in their efforts**, and an associated ability to move away from those they did not trust to be helpful. This allowed them to create 'safe spaces with safe people' when needed and involved communicating their experiences and needs to others, accessing the perspectives of others and being selective about who they involved, when and how. There's a thing that comes in there that I think is really important and that's **the ability to trust that there are other people that have got your best interests at heart.** [. . .] This is a very hard process to do alone. – Rebecca [..]<br><br>Several participants discussed having supporters **they trusted to help them observe and reflect on their wellbeing should their own awareness become impaired.** For six this included their prescribers. Four had spoken with family and friends about what objective signs they might notice leading up to a psychotic or manic episode. […]<br>I have a whole lot of [supporters] in my personal life and I have a long-term psychotherapist and a few others in a team that have worked with me for many years that know me well and are able to say, do you think you need to slow down or whatever the prior agreed course of action might be.	
(Lewins et al., 2022)	**…finding friends or relatives, as well as professionals who can provide support during medication reduction is identified as a key facilitating factor by service users (**Geyt et al., 2016; Katz, Goldblatt, Hasson-Ohayon & Roe, 2019), and empirical evidence suggests that support from family, friends or others increases the chances of successful medication discontinuation (Larsen-Barr et al., 2018). […] Decision-making processes that take account of all relevant stakeholders' views, and in which family members are involved can be part of **positive 'triangles of care' that can support the choices and needs of individuals**, and lead to courses of action that are sustainable and to which all are committed (Hannan, 2013; Morant et al., 2016).	Via Citation Search
(Pinfold et al., 2019)	Autonomy, and individual responsibility, did not mean other people and sources of information had no influence. […] The women sought, or received without asking, opinions from clinicians such as GPs, psychiatrists and midwives about medication taking in pregnancy and took on board the opinions of family and friends. These viewpoints influenced the decisions taken by each woman. **They also had to rely on others to help them enact their chosen decision, necessitating negotiation and discussion to achieve an acceptable plan: They were very supportive** because there was a long, there was quite a prelude to this about what to do about my medication. Before I conceived. So there was input from the consultant. Because it took six months, the withdrawal process, from some of my medication. So there was quite a lot of negotiation. (Agatha) […]<br><br>But I think when I had [name of baby], I was in a place where I trusted myself enough to continue my own self-care and was working on it. **I trusted the people that were around me, my family, my other half, you know, they're there as my safeguard. (Lisa) By the time I saw the psychiatrist, my mind was made up and I was just going to continue doing it. (Charlie)**	Post-2014 5*

(Salzmann-Erikson & Sjodin, 2018)	Medication with depot injection enabled the patients to gradually take more responsibility for their own lives and it facilitated the ability to meet family expectations, which were motivations for continuing with medication (Chang et al., 2013). **[…] It was considered important that the social environment supported the participants while they were on medication. Support from the family created a close social network and was described as facilitating motivation to maintain medication.** Participants reported that their family took care of their physical and mental needs and facilitated their continuing to medicate (Chang et al., 2013).	Post-2014 5*
(Watts et al., 2021)	**Participants also highly valued the support received from family members who were non-judgemental towards their decision to come off medication and provided practical support. M**y younger brother is different, we have a more equal relationship... he agrees to an advance directive that I am pushing with the psychiatrist, so I have a little bit more security in that I wouldn't be forcibly medicated.	Via Citation Search
(Zipursky et al., 2020)	Despite being made aware of the risks associated with discontinuing medications, many individuals will choose to undergo a trial off of medications. This should be done gradually with ongoing psychiatric follow-up for at least three years following discontinuation of medication. **Families should be included in discussions about risk and, together with the patient, have a clear understanding of signs of recurrence as well as a safety plan to be initiated should symptoms return.**	Via Personal Networks

CMOC 10 ('Fear and guilt')

When an individual with SMI is aware that their family members are fearful about the consequences from medication changes and want them to maintain medications as prescribed (C), they may continue on the medications against their will or secretly discontinue/change their medications (O) to avoid conflict (M) and/or withdrawal of their family's support (M) for them.

CMOC based on data extracted from 11 documents.

Document (author, date)	Extracted data	Subset of literature
(Delman et al., 2015)	Strong family relationships are associated with much better outcomes for these young adults.34 The literature does show that most parents are in favor of opportunities for young people with SMI to engage in skill development and mentoring relationships. Some parents have doubts about their child's capacity for managing their treatment and/or or understanding the nature of their condiion.35 But regardless of medication preference, young adults' participation in	Post-2014 4*

	treatment decision making can have positive effects.1 Parents can be educated on this matter, often through family psycho-education or in family therapy	
(Zhou et al., 2017)	**Chinese families are extensively involved in patients' lives regardless of subjective burden and that patients are especially responsive to urging of their families to take needed mediation** (Ran et al., 2003; Li and Lewis, 2013). For example, an investigation 606 psychiatric inpatients at Guangzhou Brain Hospital showed that 93.9% of patients lived with caregivers (He et al., 2015); as did 81.5% of patients participating in a multi-center survey in China, conducted in 2002 (Pan et al., 2003). It is also a common expectation among the Chinese that family is the first line of support in any illness and is responsible for any sick family member (Ran et al., 2003).	Post-2014 4*
(Katz et al., 2019)	To stop taking medication, participants had to possess high levels of determination, perseverance, and willpower. **First, it was necessary to deal with various social pressures, which attempted to persuade them to continue taking their medication as prescribed. Reuven described how both his formal and informal support systems (medical system and parents) resisted his intention to quit taking medication**: My parents wanted me to take the pills . . . They are very conventional . . . The word of the establishment is very strong . . . The psychiatrist is a doctor; whatever he says should be done . . . I think they [the psychiatrists] created a lot of fear in them [the parents]	Via Citation Search
(Keogh et al., 2022)	In Roe et al. (2009) study for example, pressure came from professionals, family and friends and in some cases, **participants took medication to maintain the status quo and to please others, especially their families**. In addition, one participant stated that access to a rehabilitation service was contingent on their compliance with medication (Roe et al., 2009)	Via Citation Search
(Geyt et al., 2017)	Many participants predicted that others' views on discontinuation would be discrepant with their own theories of need and costs-benefits analyses. **Where discontinuation was concerned, support from others was often appraised as unavailable or conditional on adherence, which narrowed the scope for supported discontinuation**: If I told my mum, who lives nearly 200 miles away, she'd have panicked and she'd have been like, "oh you can't not take your medication" and du-du-du, "Doctor's know what they're talking about." You know mums. Uh, I'm the one living with it.	Post-2014 5*
(Lewins et al., 2022)	Relapse places substantial strain on family members who often have little support themselves (Guti~errez-Maldonado et al., 2005). In addition, service users identify maintaining family relationships as a significant reason for adhering to their medication (Wade et al.), […]<br><br>With the exception of one respondent, family members' opinions about long-term antipsychotic medication were dominated by a pervasive fear of relapse. Periods of stability were described by family members as precious but fragile. **They expressed fear and uncertainty at the prospect of disturbing this fragile stability by reducing or discontinuing medication**. Many family members described feeling exhausted by their caring role, which ran alongside other responsibilities in their lives, and viewing medication as a tangible tool that could increase stability and prevent relapses and the consequent chaos this caused. I know they think that some people can come off the medication. But with	Via Citation Search

	clozapine… It's a dangerous one to mess around with. My opinion is: once you've stabilised something, you can't play about with it - that's it. From my experience with changing the medication, it can go very wrong – P11.[…]<br><br>**Overall, participants' concerns to maintain a degree of stability resulted in the vast majority expressing cautiousness around medication changes, particularly reductions or discontinuation.** Many indicated that they would rather not "rock the boat" after years of trial and error with medication, reflecting the severity of their relative's condition and the fragile nature of their recovery. When asked how they would feel about their child reducing medication, one parent responded: She's been on it for so long. She'd have to come off it very, very, very slow. It would be worrying the life out of me. – P01 […]<br><br>The day-to-day responsibility family members felt for this, alongside their fears about relapse, meant that participants **found it difficult to contemplate questions about the possibility of reducing or discontinuing antipsychotic medication. This sense of responsibility was such a central focus of their relationship with their relative, and indeed of their lives generally, that for most, it appeared to trump concerns about the negative effects of medication or lack of autonomy**. […]<br><br>A second overall feature of interview narratives was that family members felt deeply ambivalent about antipsychotic medication. […] tension was apparent between valuing the stabilising properties of medication, and anxiety about the harm it might be doing. […]<br><br>**Findings could also support previous literature which has indicated that some service users experience family members' involvement in overseeing their medication use as overly coercive (Rogers et al., 1998), and report that they have low levels of social support when attempting medication withdrawal** (Larsen-Barr et al., 2018). This is a concern given evidence suggesting the importance of social support for successful reduction or discontinuation of antipsychotics (Larsen-Barr & Seymour, 2021). […]<br>**When family members are strongly convinced that medication is necessary or the only way of maintaining stability, it may be difficult for users to make decisions that conflict with this position**, and this may be a significant barrier to making fully informed and autonomous healthcare decisions (Thompson et al., 2020).	
(Martinez-Hernaez et al., 2020)	For the caregivers, unawareness of disorder was a threat to family life and a source of interpersonal conflicts. Their role usually included supervising the patient's compliance with medical prescriptions, a task they understood as a "lesser evil" to ensure a harmonious family atmosphere. They considered that if patients recognise their sickness, it means that they also accept the treatment in all its terms because cessation of medication would entail a clear risk of relapse. **Caregivers experienced this risk with extreme anguish** because past episodes had involved suicide attempts, disruptive behaviours,	Post-2014<br>5*

	and in one case even sexual assault (Female Caregiver 11). They saw time as an ally, understanding that as time passes, patients move away from their position of "rebellion" and "denial" and accept that they have a disease that requires adherence to treatment, but also self-control and self-care, such as attending therapeutic meetings and medical visits, not drinking alcohol or consuming recreational drugs, and following a daily routine (i.e. hygiene, sleep, exercise)	
(Ostrow et al., 2019)	**Our findings preliminarily point to families described as "very unsupportive" reflecting critical or hostile attitudes and those described as "very supportive" reflecting emotional overinvolvement**. Some studies have included psychiatric medication compliance in their research on expressed emotion (Marquez & Ramirez Garcia, 2011;Ruscioetal., 2017;Wang et al., 2017), but none have considered the impact of high expressed emotion on people wishing to discontinue psychiatric medications.	Via Citation search
(Pinfold et al., 2019)	In addition to clinical input, **family opinions over medication were an important factor, particularly where non-congruent views were held. One woman found it hard to stay on medication during pregnancy knowing her family did not support her use of anti-psychotic medication at all**.	Post-2014 5*
(Salzmann-Erikson & Sjodin, 2018)	Patients experienced feelings of pressure to comply with medication from others in their social network (Tranulis et al., 2011; Svedberg et al., 2003; Carrick et al., 2004). In one study (Tranulis et al., 2011), 60% of participants stated that they stopped taking medication as they did not find it necessary. **However, this "non-compliance" was met with strong pressure from family and friends to resume medication, such as continued medication being a condition for a participant to stay with his mother. […] One study emphasized that lack of support from family members was a reason patients stopped taking medication** (Teferra et al., 2013). In that study, one participant reported that a relative who had previously been supportive withdrew that support, even though the participant asked for this person to accompany them.	Post-2014 5*
(Watts et al., 2021)	**Many participants did not believe their family would support decision, so very few consulted with family members.** Of those who did, the most frequent response was one that reinforced the narrative of strictly adhering to medication prescribed by the 'medical experts'. You just have to believe your doctor and you have to take your medication'.. . they [family] just did not understand.	Via Citation Search

CMOC 11 ('Peer support positive')

When individuals with SMI have access to Peer Support Workers (PSWs) with shared lived experiences who talk with them about SMI and life skills management, including medications and side effects (C), they are apt to experience a positive impact on their mental, physical and social-emotional health (O) because they feel validated (M) less stigmatized (M) and reassured (M) that they can have productive, fulfilling lives with SMI.

CMOC based on data extracted from 6 documents.

Document (author, date)	Extracted data	Subset of literature
(Clifford et al., 2020)	Interviewees commonly reported that contact with other consumers or with **peer workers had a positive role in assisting with adherence due to shared experiences of taking antipsychotic medication and mutual understanding of mental illness. Peer workers were described as "valuable resources" representing positive role models for consumers as they were "stable".** […] Interviewees consistently described their experiences with case workers and community centres positively. They supported the role of peer workers in assisting with adherence due to their **shared experiences**. One interviewee reported an enhanced sense of purpose associated with working as a peer worker, consistent with recovery research, which relates consumers' involvement in such programs to empowerment (Vandewalle, Debyser, & Beeckman, 2018).	Post-2014 4*
(Coulthard et al., 2013)	There is an increasing use of peer specialists (PS, defined as individuals with severe mental illness who use their experience to provide services for other people with severe mental illness) in mental health services, particularly in the United States of America [14,15]. It is argued that this development is supported by **social learning theory [16] and that PSs can effectively teach coping and self-management strategies [17] and further that PSs often increase patients' engagement and improve patient satisfaction [14,18]. There is now experience of PSs facilitating groups in mental health settings e.g. [19].** […]<br><br>Overall the treatment groups resulted **in improvements in agency i.e. a greater willingness and confidence to take control of their illness with a view to preventing relapse or reduce its overall impact.**	Additional Search (Peer Support)
(Ehrlich & Dannapfel, 2017)	Participants also valued the opportunity to be with someone who they could talk to: "I just like seeing another person during the day. So doesn't matter what's happening really, just breaks it up a bit for me…" (ID031; man, 31 years). In this way, **support workers assisted participants to become more physically and socially active and motivated them to focus on things other than their mental health, which helped them integrate into the community**. Support workers also had a positive impact destructive behaviours such as alcohol mis-use: "… [named support workers] come around and that helps me stay off alcohol until a certain time in the afternoon" (ID017; woman, 33 years).	Post-2014 5*

(Evans et al., 2021)	PWS=people with schizophrenia<br><br>One promising strategy is services delivered by peers—people who share a lived experience with those they support. Based on the principles of self-help and mutual support (Davidson et al., 2006), peers deliver a range of services, including some traditionally provided by mental health professionals, such as case management, as well as in roles specifically designed for peers (Davidson et al., 2006). […]<br><br>Researchers hypothesize that one of the ways in which peer-delivered services may work is through alleviating stigma (Gillard et al., 2015; Pyle et al., 2018). Five studies (23%) discussed stigma. In two qualitative studies, **participants relayed that their experience with peers helped them gain self-confidence and decrease self-stigma** (Graham et al., 2017; van Langen et al., 2016). Quantitatively, one study found no change in stigma beliefs among consumers as a result of the intervention (Cook, 2014). However, the ability to detect an effect of the intervention was compromised by low initial levels of reported stigma in the sample. […]<br><br>PWS reported that hearing others openly share their experiences with psychosis helped them see their psychotic symptoms as a part of mental illness, not as deviance unique to themselves (van Langen et al., 2016). **They identified that mutual sharing with others who understood their problems made them feel free to make their own decisions, something they found lacking in sessions with professionals** (van Langen et al., 2016). […]<br><br>The lived experience of mental illness may differ significantly depending on the disorder so that having **a shared lived experience of psychosis** might provide more commonality than a shared lived experience of mental illness or substance abuse more broadly. […]<br><br>..-Specific peer interventions may provide a much less stigmatizing environment than interventions delivered to mixed-diagnosis groups.	Additional Search (peer support)
(Hansen et al., 2018)	The **reciprocity and feelings of togetherness** experienced in peer groups were also pointed out as helpful and meaningful in several ways. Simply getting to know other people who struggled with similar issues made many participants feel more positive toward engagement with services. In these groups, participants usually experienced genuine empathy and understanding as well as instillation of hope from others who had dealt successfully with their own symptoms and distress. **Many participants found the groups to be helpful in developing coping strategies and gaining more control over their experience**. Helping others was also perceived as an important part of engaging in these groups. […]	Via Citation Search

	Nevertheless, they strongly underlined how being met and treated as people significantly influenced their recovery; a theme we have named it's about people. Included in this theme, are also peer relationships as well as friends and family relations.	
(King et al., 2022)	Describing withdrawal effects to healthcare providers was challenging and it was common to that only peers appreciated how challenging symptoms could be from peers.<br>"I found hundreds of other people that were saying exactly the same that I was, having exactly the same withdrawal symptoms as I was, and even when I printed that out and took it with me I wasn't believed" – Harriet	Via Personal Networks

REFERENCES

Alguera-Lara, V., Dowsey, M. M., Ride, J., Kinder, S., & Castle, D. (2017). Shared decision making in mental health: the importance for current clinical practice. *Australasian psychiatry : bulletin of Royal Australian and New Zealand College of Psychiatrists*, *25*(6), 578-582. http://ovidsp.ovid.com/ovidweb.cgi?T=JS&PAGE=reference&D=med14&NEWS=N&AN=29017332

Aref-Adib, G., O'Hanlon, P., Fullarton, K., Morant, N., Sommerlad, A., Johnson, S., & Osborn, D. (2016). A qualitative study of online mental health information seeking behaviour by those with psychosis. *BMC Psychiatry*, *16*, 232. http://ovidsp.ovid.com/ovidweb.cgi?T=JS&PAGE=reference&D=med13&NEWS=N&AN=27400874https://www.ncbi.nlm.nih.gov/pmc/articles/PMC4940927/pdf/12888_2016_Article_952.pdf

Bjornestad, J., Lavik, K. O., Davidson, L., Hjeltnes, A., Moltu, C., & Veseth, M. (2020). Antipsychotic treatment – a systematic literature review and meta-analysis of qualitative studies. *Journal of Mental Health*, *29*(5), 513-523. https://search.ebscohost.com/login.aspx?direct=true&db=cin20&AN=146195961&site=ehost-live&authtype=ip,uidhttps://www.tandfonline.com/doi/full/10.1080/09638237.2019.1581352

Bolden, G. B., Angell, B., & Hepburn, A. (2019). How clients solicit medication changes in psychiatry. *Sociology of Health & Illness*, *41*(2), 411-426. https://www.proquest.com/scholarly-journals/how-clients-solicit-medication-changes-psychiatry/docview/2174600299/se-2 http://oxfordsfx.hosted.exlibrisgroup.com/oxford?url_ver=Z39.88-2004&rft_val_fmt=info:ofi/fmt:kev:mtx:journal&genre=article&sid=ProQ:ProQ%3Asocabs&atitle=How+clients+solicit+medication+changes+in+psychiatry&title=Sociology+of+Health+%26+Illness&issn=01419889&date=2019-02-01&volume=41&issue=2&spage=411&au=Bolden%2C+Galina+B%3BAngell%2C+Beth%3BHepburn%2C+Alexa&isbn=&jtitle=Sociology+of+Health+%26+Illness&btitle=&rft_id=info:eric/&rft_id=info:doi/10.1111%2F1467-9566.12843https://ezproxy-prd.bodleian.ox.ac.uk:2152/doi/full/10.1111/1467-9566.12843

Carrick, R., Mitchell, A., Powell, R. A., & Lloyd, K. (2004). The quest for well-being: A qualitative study of the experience of taking antipsychotic medication. *Psychology and Psychotherapy: Theory, Research and Practice*, *77*(1), 19-33.

Clifford, L., Crabb, S., Turnbull, D., Hahn, L., & Galletly, C. (2020). A qualitative study of medication adherence amongst people with schizophrenia. *Archives of psychiatric nursing*, *34*(4), 194-199. http://ovidsp.ovid.com/ovidweb.cgi?T=JS&PAGE=reference&D=med18&NEWS=N&AN=32828348https://www.psychiatricnursing.org/article/S0883-9417(20)30045-5/fulltext

Cooper, R. E., Mason, J. P., Calton, T., Richardson, J., & Moncrieff, J. (2021). Opinion Piece: The case for establishing a minimal medication alternative for psychosis and schizophrenia. *Psychosis*, 1-10. https://doi.org/10.1080/17522439.2021.1930119

Coulthard, K., Patel, D., Brizzolara, C., Morriss, R., & Watson, S. (2013). A feasibility study of expert patient and community mental health team led bipolar psychoeducation groups: implementing an evidence based practice. *BMC Psychiatry*, *13*(1), 1-12.

Crellin, N. E., Priebe, S., Morant, N., Lewis, G., Freemantle, N., Johnson, S., Horne, R., Pinfold, V., Kent, L., & Smith, R. (2022). An analysis of views about supported reduction or discontinuation of antipsychotic treatment among people with schizophrenia and other psychotic disorders. *BMC Psychiatry*, *22*(1), 1-11.

Delman, J., Clark, J. A., Eisen, S. V., & Parker, V. A. (2015). Facilitators and barriers to the active participation of clients with serious mental illnesses in medication decision making: the perceptions of young adult clients. *The journal of behavioral health services & research*, *42*(2), 238-253. http://ovidsp.ovid.com/ovidweb.cgi?T=JS&PAGE=reference&D=med12&NEWS=N&AN=25056768

Ehrlich, C., & Dannapfel, P. (2017). Shared decision making: People with severe mental illness experiences of involvement in the care of their physical health. *Mental Health and Prevention*, *5*, 21-26. http://ovidsp.ovid.com/ovidweb.cgi?T=JS&PAGE=reference&D=psyc14&NEWS=N&AN=2017-11199-004

Evans, M., Barker, H., Peddireddy, S., Zhang, A., Luu, S., Qian, Y., Tang, P. Y., & Fisher, E. B. (2021). Peer-delivered services and peer support reaching people with schizophrenia: A scoping review. *International Journal of Mental Health*, 1-23.

Fiore, G., Bertani, D. E., Marchi, M., Cardoso, G., & Galeazzi, G. M. (2021). Patient subjective experience of treatment with long-acting injectable antipsychotics: a systematic review of qualitative studies. *Jornal Brasileiro de Psiquiatria*, *70*, 68-77.

Fiorillo, A., Barlati, S., Bellomo, A., Corrivetti, G., Nicolo, G., Sampogna, G., Stanga, V., Veltro, F., Maina, G., & Vita, A. (2020). The role of shared decision-making in improving adherence to pharmacological treatments in patients with schizophrenia: a clinical review. *Annals of general psychiatry*, *19*, 43. http://ovidsp.ovid.com/ovidweb.cgi?T=JS&PAGE=reference&D=pmnm5&NEWS=N&AN=32774442https://www.ncbi.nlm.nih.gov/pmc/articles/PMC7409631/pdf/12991_2020_Article_293.pdf

Garcia, S., Martinez-Cengotitabengoa, M., Lopez-Zurbano, S., Zorrilla, I., Lopez, P., Vieta, E., & Gonzalez-Pinto, A. (2016). Adherence to Antipsychotic Medication in Bipolar Disorder and Schizophrenic Patients: A Systematic Review. *Journal of Clinical Psychopharmacology*, *36*(4), 355-371. http://ovidsp.ovid.com/ovidweb.cgi?T=JS&PAGE=reference&D=med13&NEWS=N&AN=27307187https://www.ncbi.nlm.nih.gov/pmc/articles/PMC4932152/pdf/jcp-36-355.pdf

Geyt, G. L., Awenat, Y., Tai, S., & Haddock, G. (2016). Personal Accounts of Discontinuing Neuroleptic Medication for Psychosis. *Qualitative Health Research*, *27*(4), 559-572. https://doi.org/10.1177/1049732316634047

Geyt, G. L., Awenat, Y., Tai, S., & Haddock, G. (2017). Personal Accounts of Discontinuing Neuroleptic Medication for Psychosis. *Qualitative Health Research*, *27*(4), 559-572. https://search.ebscohost.com/login.aspx?direct=true&db=cin20&AN=121334782&site=ehost-live&authtype=ip,uid

Green, C. A., Polen, M. R., Janoff, S. L., Castleton, D. K., Wisdom, J. P., Vuckovic, N., Perrin, N. A., Paulson, R. I., & Oken, S. L. (2008). Understanding how clinician-patient relationships and relational continuity of care affect recovery from serious mental illness: STARS study results. *Psychiatric rehabilitation journal*, *32*(1), 9.

Grunwald, L. M., Duddy, C., Byng, R., Crellin, N., & Moncrieff, J. (2021). The role of trust and hope in antipsychotic medication reviews between GPs and service users a realist review. *BMC Psychiatry*, *21*(1), 390. https://bmcpsychiatry.biomedcentral.com/ http://ovidsp.ovid.com/ovidweb.cgi?T=JS&PAGE=reference&D=emexa&NEWS=N&AN=2013376226https://www.ncbi.nlm.nih.gov/pmc/articles/PMC8340528/pdf/12888_2021_Article_3355.pdf

Grünwald, L. M., & Thompson, J. (2021). Re-starting the conversation: improving shared decision making in antipsychotic prescribing. *Psychosis*, *13*(4), 373-377. https://search.ebscohost.com/login.aspx?direct=true&db=cin20&AN=153842560&site=ehost-live&authtype=ip,uid

Haddad, P. M., Brain, C., & Scott, J. (2014). Nonadherence with antipsychotic medication in schizophrenia: challenges and management strategies. *Patient related outcome measures*, *5*, 43-62. http://ovidsp.ovid.com/ovidweb.cgi?T=JS&PAGE=reference&D=pmnm&NEWS=N&AN=25061342https://www.dovepress.com/getfile.php?fileID=20643

Hansen, H., Stige, S. H., Davidson, L., Moltu, C., & Veseth, M. (2018). How do people experience early intervention services for psychosis? A meta-synthesis. *Qualitative Health Research*, *28*(2), 259-272.

Haugom, E. W., Stensrud, B., Beston, G., Ruud, T., & Landheim, A. S. (2020). Mental health professionals' experiences with shared decision-making for patients with psychotic disorders: a qualitative study. *BMC health services research*, *20*(1), 1093. http://ovidsp.ovid.com/ovidweb.cgi?T=JS&PAGE=reference&D=med18&NEWS=N&AN=33246451https://www.ncbi.nlm.nih.gov/pmc/articles/PMC7694931/pdf/12913_2020_Article_5949.pdf

Jakovljevic, M. (2014). LONG-ACTING INJECTABLE (DEPOT) ANTIPSYCHOTICS AND CHANGING TREATMENT PHILOSOPHY: POSSIBLE CONTRIBUTION TO INTEGRATIVE CARE AND PERSONAL RECOVERY OF SCHIZOPHRENIA. *PSYCHIATRIA DANUBINA*, *26*(4), 304-307.

Jawad, I., Watson, S., Haddad, P. M., Talbot, P. S., & McAllister-Williams, R. H. (2018). Medication nonadherence in bipolar disorder: a narrative review. *Therapeutic Advances in Psychopharmacology*, *8*(12), 349-363. http://ovidsp.ovid.com/ovidweb.cgi?T=JS&PAGE=reference&D=pmnm4&NEWS=N&AN=30524703https://www.ncbi.nlm.nih.gov/pmc/articles/PMC6278745/pdf/10.1177_2045125318804364.pdf

Kaar, S. J., Gobjila, C., Butler, E., Henderson, C., & Howes, O. D. (2019). Making decisions about antipsychotics: a qualitative study of patient experience and the development of a decision aid. *BMC Psychiatry*, *19*(1), 309. http://ovidsp.ovid.com/ovidweb.cgi?T=JS&PAGE=reference&D=med16&NEWS=N&AN=31646985https://www.ncbi.nlm.nih.gov/pmc/articles/PMC6806500/pdf/12888_2019_Article_2304.pdf

Kaminskiy, E., Zisman-Ilani, Y., Morant, N., & Ramon, S. (2021). Barriers and enablers to shared decision making in psychiatric medication management: A qualitative investigation of clinician and service users' views. *Frontiers in psychiatry*, *12*, 678005.

Katz, S., Goldblatt, H., Hasson-Ohayon, I., & Roe, D. (2019). Retrospective accounts of the process of using and discontinuing psychiatric medication. *Qualitative Health Research*, *29*(2), 198-210.

Keogh, B., Murphy, E., Doyle, L., Sheaf, G., Watts, M., & Higgins, A. (2022). Mental health service users experiences of medication discontinuation: a systematic review of qualitative studies. *Journal of Mental Health*, *31*(2), 227-238.

King, S. R., Allan, M., & Lindsey, L. (2022). “I found hundreds of other people… but I still wasn’t believed”–An exploratory study on lived experiences of antipsychotic withdrawal. *Psychosis*, 1-13.

Larsen-Barr, M., & Seymour, F. (2021). Service-user efforts to maintain their wellbeing during and after successful withdrawal from antipsychotic medication. *Therapeutic Advances in Psychopharmacology*, *11*, 2045125321989133.

Larsen-Barr, M., Seymour, F., Read, J., & Gibson, K. (2018). Attempting to discontinue antipsychotic medication: Withdrawal methods, relapse and success. *Psychiatry research*, *270*, 365-374. https://doi.org/https://doi.org/10.1016/j.psychres.2018.10.001

Leclerc, E., Noto, C., Bressan, R. A., & Brietzke, E. (2015). Determinants of adherence to treatment in first-episode psychosis: a comprehensive review. *Revista brasileira de psiquiatria (Sao Paulo, Brazil : 1999)*, *37*(2), 168-176. http://ovidsp.ovid.com/ovidweb.cgi?T=JS&PAGE=reference&D=med12&NEWS=N&AN=25946398https://www.scielo.br/j/rbp/a/cY4L8Wd3KKRB6jLgP7yjTfc/?lang=en&format=pdf

Lewins, A., Morant, N., Akther-Robertson, J., Crellin, N. E., Stansfeld, J. L., Smith, R., & Moncrieff, J. (2022). A qualitative exploration of family members' perspectives on reducing and discontinuing antipsychotic medication. *Journal of Mental Health*, 1-8.

Lim, M., Li, Z., Xie, H., Tan, B. L., & Lee, J. (2021). The Effect of Therapeutic Alliance on Attitudes Toward Psychiatric Medications in Schizophrenia. *Journal of Clinical Psychopharmacology*, *41*(5), 551-560. http://ovidsp.ovid.com/ovidweb.cgi?T=JS&PAGE=reference&D=med19&NEWS=N&AN=34411007

Mahone, I. H., Maphis, C. F., & Snow, D. E. (2016). Effective Strategies for Nurses Empowering Clients With Schizophrenia: Medication Use as a Tool in Recovery. *Issues in mental health nursing*, *37*(5), 372-379. http://ovidsp.ovid.com/ovidweb.cgi?T=JS&PAGE=reference&D=med13&NEWS=N&AN=27111300https://www.ncbi.nlm.nih.gov/pmc/articles/PMC4898146/pdf/imhn-37-372.pdf

Maj, M., van Os, J., De Hert, M., Gaebel, W., Galderisi, S., Green, M. F., Guloksuz, S., Harvey, P. D., Jones, P. B., Malaspina, D., McGorry, P., Miettunen, J., Murray, R. M., Nuechterlein, K. H., Peralta, V., Thornicroft, G., van Winkel, R., & Ventura, J. (2021). The clinical characterization of the patient with primary psychosis aimed at personalization of management. *World psychiatry : official journal of the World Psychiatric Association (WPA)*, *20*(1), 4-33. http://ovidsp.ovid.com/ovidweb.cgi?T=JS&PAGE=reference&D=pmnm5&NEWS=N&AN=33432763https://www.ncbi.nlm.nih.gov/pmc/articles/PMC7801854/pdf/WPS-20-4.pdf

Martinez-Hernaez, A., Pie-Balaguer, A., Serrano-Miguel, M., Morales-Saez, N., Garcia-Santesmases, A., Bekele, D., & Alegre-Agis, E. (2020). The collaborative management of antipsychotic medication and its obstacles: A qualitative study. *Social science & medicine (1982)*, *247*, 112811. http://ovidsp.ovid.com/ovidweb.cgi?T=JS&PAGE=reference&D=medp&NEWS=N&AN=32032839

Matthias, M. S., Salyers, M. P., Rollins, A. L., & Frankel, R. M. (2012). Decision making in recovery-oriented mental health care. *Psychiatric rehabilitation journal*, *35*(4), 305.

Morant, N., Azam, K., Johnson, S., & Moncrieff, J. (2018). The least worst option: user experiences of antipsychotic medication and lack of involvement in medication decisions in a UK community sample. *Journal of mental health (Abingdon, England)*, *27*(4), 322-328. http://ovidsp.ovid.com/ovidweb.cgi?T=JS&PAGE=reference&D=med15&NEWS=N&AN=28857636https://www.tandfonline.com/doi/full/10.1080/09638237.2017.1370637

Oedegaard, C. H., Davidson, L., Stige, B., Veseth, M., Blindheim, A., Garvik, L., Sørensen, J.-M., Søraa, Ø., & Engebretsen, I. M. S. (2020). "It means so much for me to have a choice": a qualitative study providing first-person perspectives on medication-free treatment in mental health care. *BMC Psychiatry*, *20*(1), 399. https://doi.org/10.1186/s12888-020-02770-2

Opler, L. A., Ramirez, P. M., Dominguez, L. M., Fox, M. S., & Johnson, P. B. (2004). Rethinking medication prescribing practices in an inner-city Hispanic mental health clinic. *Journal of Psychiatric Practice®*, *10*(2), 134-140.

Ostrow, L., Croft, B., Weaver, A., & Naeger, S. (2019). An exploratory analysis of the role of social supports in psychiatric medication discontinuation: results related to family involvement. *Psychosis*, *11*(3), 212-222.

Pedley, R., McWilliams, C., Lovell, K., Brooks, H., Rushton, K., Drake, R. J., Rumbold, B., Bell, V., & Bee, P. (2018). Qualitative systematic review of barriers and facilitators to patient-involved antipsychotic prescribing. *BJPsych open*, *4*(1), 5-14. http://ovidsp.ovid.com/ovidweb.cgi?T=JS&PAGE=reference&D=pmnm4&NEWS=N&AN=29388908https://www.cambridge.org/core/services/aop-cambridge-core/content/view/C281C0F17A63E16A825BB9078779E32B/S2056472417000059a.pdf/div-class-title-qualitative-systematic-review-of-barriers-and-facilitators-to-patient-involved-antipsychotic-prescribing-div.pdf

Phan, S. V. (2016). Medication adherence in patients with schizophrenia. *International journal of psychiatry in medicine*, *51*(2), 211-219. http://ovidsp.ovid.com/ovidweb.cgi?T=JS&PAGE=reference&D=med13&NEWS=N&AN=27079779

Pietrini, F., Albert, U., Ballerini, A., Calo, P., Maina, G., Pinna, F., Vaggi, M., Boggian, I., Fontana, M., Moro, C., & Carpiniello, B. (2019). The modern perspective for long-acting injectables antipsychotics in the patient-centered care of schizophrenia. *Neuropsychiatric disease and treatment*, *15*, 1045-1060. http://ovidsp.ovid.com/ovidweb.cgi?T=JS&PAGE=reference&D=pmnm4&NEWS=N&AN=31118640https://www.dovepress.com/getfile.php?fileID=49452

Pinfold, V., Dare, C., Hamilton, S., Kaur, H., Lambley, R., Nicholls, V., Petersen, I., Szymczynska, P., Walker, C., & Stevenson, F. (2019). Anti-psychotic medication decision making during pregnancy: A co-produced research study. *Mental Health Review Journal*, *24*(2), 69-84. http://ovidsp.ovid.com/ovidweb.cgi?T=JS&PAGE=reference&D=psyc16&NEWS=N&AN=2019-38829-001

Quirk, A., Chaplin, R., Hamilton, S., Lelliott, P., & Seale, C. (2013). Communication about adherence to long-term antipsychotic prescribing: an observational study of psychiatric practice. *Social Psychiatry and Psychiatric Epidemiology*, *48*, 639-647.

Roberts, R., Neasham, A., Lambrinudi, C., & Khan, A. (2018). A thematic analysis assessing clinical decision-making in antipsychotic prescribing for schizophrenia. *BMC Psychiatry*, *18*(1), 290. http://ovidsp.ovid.com/ovidweb.cgi?T=JS&PAGE=reference&D=med15&NEWS=N&AN=30200923https://www.ncbi.nlm.nih.gov/pmc/articles/PMC6131851/pdf/12888_2018_Article_1872.pdf

Rungruangsiripan, M., Sitthimongkol, Y., Maneesriwongul, W., Talley, S., & Vorapongsathorn, T. (2011). Mediating role of illness representation among social support, therapeutic alliance, experience of medication side effects, and medication adherence in persons with schizophrenia. *Archives of psychiatric nursing*, *25*(4), 269-283.

Salzmann-Erikson, M., & Sjodin, M. (2018). A narrative meta-synthesis of how people with schizophrenia experience facilitators and barriers in using antipsychotic medication: Implications for healthcare professionals. *International journal of nursing studies*, *85*, 7-18. http://ovidsp.ovid.com/ovidweb.cgi?T=JS&PAGE=reference&D=med15&NEWS=N&AN=29803018

Seale, C., Chaplin, R., Lelliott, P., & Quirk, A. (2006). Sharing decisions in consultations involving anti-psychotic medication: a qualitative study of psychiatrists' experiences. *Social science & medicine*, *62*(11), 2861-2873.

Slade, M. (2017). Implementing shared decision making in routine mental health care. *World Psychiatry*, *16*(2), 146-153. https://doi.org/https://doi.org/10.1002/wps.20412

Sowerby, C., & Taylor, D. (2017). Cross-sector user and provider perceptions on experiences of shared-care clozapine: a qualitative study. *BMJ open*, *7*(9), e017183. http://ovidsp.ovid.com/ovidweb.cgi?T=JS&PAGE=reference&D=med14&NEWS=N&AN=28963298https://www.ncbi.nlm.nih.gov/pmc/articles/PMC5640131/pdf/bmjopen-2017-017183.pdf

Steingard, S. (2018). Five year outcomes of tapering antipsychotic drug doses in a community mental health center. *Community mental health journal*, *54*(8), 1097-1100.

Thompson, J., Stansfeld, J. L., Cooper, R. E., Morant, N., Crellin, N. E., & Moncrieff, J. (2020). Experiences of taking neuroleptic medication and impacts on symptoms, sense of self and agency: a systematic review and thematic synthesis of qualitative data. *Social Psychiatry and Psychiatric Epidemiology*, *55*(2), 151-164.

Velligan, D. I., Sajatovic, M., Hatch, A., Kramata, P., & Docherty, J. P. (2017). Why do psychiatric patients stop antipsychotic medication? A systematic review of reasons for nonadherence to medication in patients with serious mental illness. *Patient Preference and Adherence*, *11*, 449-468. http://ovidsp.ovid.com/ovidweb.cgi?T=JS&PAGE=reference&D=pmnm4&NEWS=N&AN=28424542https://www.dovepress.com/getfile.php?fileID=35301

Watts, M., Murphy, E., Keogh, B., Downes, C., Doyle, L., & Higgins, A. (2021). Deciding to discontinue prescribed psychotropic medication: A qualitative study of service users' experiences. *International journal of mental health nursing*, *30*, 1395-1406.

Weiden, P. J. (2016). Redefining Medication Adherence in the Treatment of Schizophrenia: How Current Approaches to Adherence Lead to Misinformation and Threaten Therapeutic Relationships. *Psychiatric Clinics of North America*, *39*(2), 199-216. http://www.elsevier.com/inca/publications/store/6/2/3/3/7/3/index.htt http://ovidsp.ovid.com/ovidweb.cgi?T=JS&PAGE=reference&D=emed17&NEWS=N&AN=608939348https://www.sciencedirect.com/science/article/abs/pii/S0193953X16000058?via%3Dihub No access via Oxford

White, E., Read, J., & Julo, S. (2021). The role of Facebook groups in the management and raising of awareness of antidepressant withdrawal: is social media filling the void left by health services? *Therapeutic Advances in Psychopharmacology*, *11*, 2045125320981174. https://doi.org/10.1177/2045125320981174

Yeisen, R. A. H., Bjornestad, J., Joa, I., Johannessen, J. O., & Opjordsmoen, S. (2017). Experiences of antipsychotic use in patients with early psychosis: a two-year follow-up study. *BMC Psychiatry*, *17*(1), 299. http://ovidsp.ovid.com/ovidweb.cgi?T=JS&PAGE=reference&D=med14&NEWS=N&AN=28830453https://www.ncbi.nlm.nih.gov/pmc/articles/PMC5567881/pdf/12888_2017_Article_1425.pdf

Younas, M., Bradley, E., Holmes, N., Sud, D., & Maidment, I. D. (2016). Mental health pharmacists views on shared decision-making for antipsychotics in serious mental illness. *International Journal of Clinical Pharmacy*, *38*(5), 1191-1199. http://ovidsp.ovid.com/ovidweb.cgi?T=JS&PAGE=reference&D=med13&NEWS=N&AN=27450504https://www.ncbi.nlm.nih.gov/pmc/articles/PMC5031729/pdf/11096_2016_Article_352.pdf

Zhou, Y., Rosenheck, R., Mohamed, S., Ning, Y., & He, H. (2017). Factors associated with complete discontinuation of medication among patients with schizophrenia in the year after hospital discharge. *Psychiatry research*, *250*, 129-135. http://ovidsp.ovid.com/ovidweb.cgi?T=JS&PAGE=reference&D=med14&NEWS=N&AN=28160655https://www.sciencedirect.com/science/article/abs/pii/S0165178116303262?via%3Dihub

Zipursky, R. B., Odejayi, G., Agid, O., & Remington, G. (2020). You say "schizophrenia" and I say "psychosis": just tell me when I can come off this medication. *Schizophrenia Research*, *225*, 39-46.

Zisman-Ilani, Y., Hurford, I., Bowen, A., Salzer, M., & Thomas, E. C. (2021). Evaluating the feasibility of a decision aid to promote shared decision making among young adults with first-episode psychosis: protocol for a pilot study. *Pilot and feasibility studies*, *7*(1), 22. http://ovidsp.ovid.com/ovidweb.cgi?T=JS&PAGE=reference&D=pmnm5&NEWS=N&AN=33431018https://www.ncbi.nlm.nih.gov/pmc/articles/PMC7798319/pdf/40814_2020_Article_757.pdf
